# Design, synthesis, and mechanistic evaluation of novel pyrazole/thiazole chalcone hybrids as dual tubulin polymerization and COX-2 inhibitors with potent antiproliferative activity

**DOI:** 10.1039/d6ra03557d

**Published:** 2026-06-02

**Authors:** Basima A. A. Saleem, Ashraf A. Qurtam, Abdelrahman R. Shalabi, Mohammed Al-zharani, Kasim Sakran Abass, Stefan Bräse, Ghallab Alotaibi, Abdullah Alkhammash

**Affiliations:** a Department of Chemistry, College of Science, University of Mosul Mosul 41001 Iraq; b Biology Department, College of Science, Imam Mohammad Ibn Saud Islamic University (IMSIU) Riyadh 11623 Saudi Arabia; c Pharmaceutical Chemistry Department, Faculty of Pharmacy, Sinai University North Sinai Egypt; d Department of Physiology, Biochemistry and Pharmacology, College of Veterinary Medicine, University of Kirkuk Kirkuk 36001 Iraq kasim_abass@uokirkuk.edu.iq; e Institute of Biological and Chemical Systems—Functional Molecular Systems (IBCS-FMS), Karlsruhe Institute of Technology (KIT) Kaiserstrasse 12 76131 Karlsruhe Germany; f Department of Pharmacology, College of Pharmacy, Al-Dawadmi Campus, Shaqra University Shaqra 11961 Saudi Arabia

## Abstract

A novel series of pyrazole/thiazole chalcone hybrids (9a–o) was designed, synthesized, and evaluated as dual tubulin/COX-2 inhibitors with anticancer activity. The synthesized compounds were screened for antiproliferative activity against MDA-MB-231, HCA-7, and A549 cancer cell lines. Several derivatives exhibited promising activity, with 9m being the most potent against MDA-MB-231 cells (IC_50_ = 1.96 ± 0.10 µM), while 9l emerged as the most balanced lead compound, showing strong antiproliferative activity against HCA-7, MDA-MB-231, and A549 cells with IC_50_ values of 2.18 ± 0.11, 2.92 ± 0.15, and 4.86 ± 0.25 µM, respectively. Mechanistic studies revealed that the anticancer activity of this series is mediated through a dual mechanism involving tubulin polymerization inhibition and selective COX-2 inhibition. In particular, compound 9l inhibited tubulin polymerization with an IC_50_ of 4.21 ± 0.25 µM and showed potent COX-2 inhibition (IC_50_ = 0.10 ± 0.01 µM) with high selectivity over COX-1 (IC_50_ = 10.92 ± 0.78 µM; selectivity index = 109.20). Further investigation in HCA-7 cells demonstrated that 9l significantly increased Bax level to 438.64 ± 15.72 pg mL^−1^ and reduced Bcl-2 to 6.74 ± 0.19 pg mL^−1^, while markedly elevating caspase-3 and caspase-9 levels to 496.80 ± 14.90 pg mL^−1^ and 47.86 ± 1.18 ng mL^−1^, respectively. Moreover, 9l strongly suppressed PGE-2 production to 0.56 ± 0.04 ng mL^−1^, corresponding to 89.2% inhibition, and induced G2/M cell-cycle arrest. It also showed promising anti-migratory activity in the wound-healing assay, favorable microsomal stability, and acceptable *in silico* ADMET properties. Molecular docking further supported its favorable binding within the active sites of both tubulin and COX-2. Collectively, these findings identify 9l as a promising dual tubulin/COX-2-targeting anticancer candidate.

## Abstract 2

A novel series of pyrazole/thiazole chalcone hybrids (9a–o) was designed, synthesized, and evaluated as dual tubulin/COX-2 inhibitors with anticancer activity.

## Graphical abstract

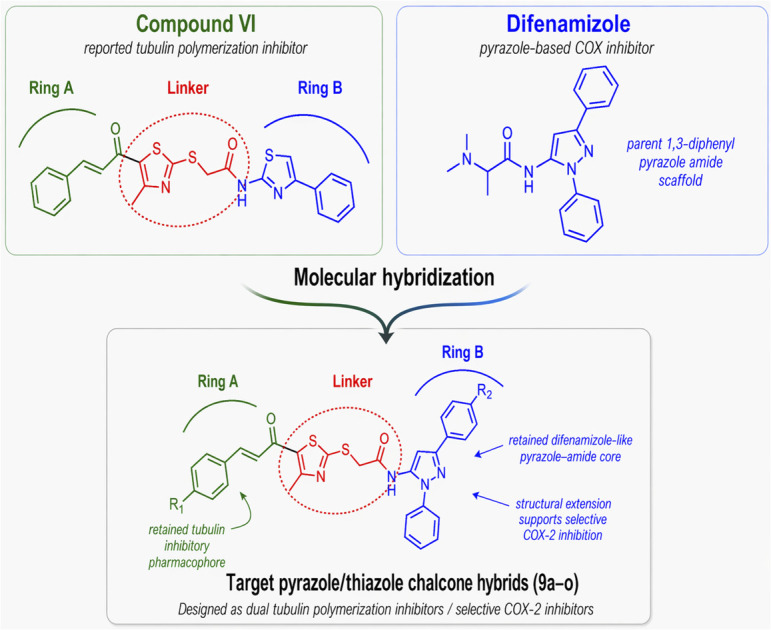

## 1. Introduction

Cancer remains one of the leading causes of death worldwide and continues to impose a major therapeutic burden despite substantial advances in surgery, radiotherapy, and systemic treatment.^1^ Its lethality is driven not only by uncontrolled proliferation but also by invasion, metastasis, and the progressive acquisition of resistance to therapy.^2,3^ According to the World Health Organization, cancer accounted for nearly 10 million deaths in 2020,^4^ and roughly one-third of cancer deaths are linked to modifiable risk factors such as tobacco use, alcohol consumption, obesity, unhealthy diet, and physical inactivity. These realities continue to justify the search for mechanistically innovative small molecules capable of interfering with multiple cancer-relevant pathways simultaneously.^5–8^

Among validated anticancer strategies, disruption of microtubule dynamics remains one of the most successful approaches in medicinal chemistry.^9^ Microtubules are dynamic polymers of α/β-tubulin heterodimers that are essential for cell architecture, intracellular transport, and, most importantly, chromosome segregation during mitosis.^10^ Agents that inhibit tubulin polymerization suppress spindle formation, arrest cells at the G2/M phase, and ultimately promote apoptotic death.^11^ Within this class, ligands that target the colchicine-binding site are especially attractive because they prevent tubulin from adopting the conformation required for microtubule assembly, and combretastatin A-4 (CA-4) remains one of the best-known prototypes.^12^ However, the therapeutic exploitation of CA-4 has been limited by drawbacks associated with its *cis*-stilbene system, especially its tendency to lose the active geometry and related pharmaceutical limitations,^13^ which has encouraged the development of structurally constrained mimics and heterocyclic replacements.

**Fig. 1:**
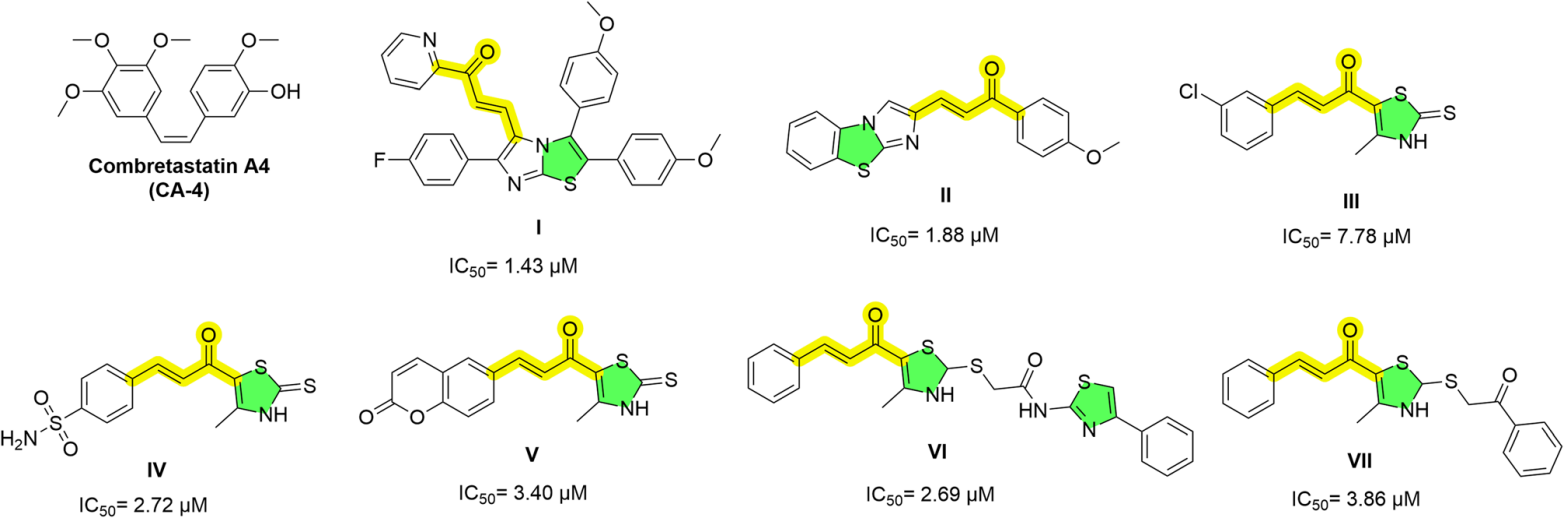
Selected thiazole/chalcone-based tubulin polymerization inhibitors (I–VII) and combretastatin A-4.

In this context, both thiazoles and chalcones have emerged as highly valuable motifs in the design of tubulin polymerization inhibitors.^14^ Thiazole-containing scaffolds are well-established in anticancer drug discovery, where they serve as rigid heteroaromatic frameworks suitable for colchicine-site binding.^15–17^ Likewise, chalcones offer a privileged aryl–enone scaffold capable of occupying hydrophobic regions within the tubulin pocket and promoting antiproliferative activities.^18,19^ The [2,1-*b*]thiazole–chalcone conjugate I illustrates this, exhibiting broad cytotoxicity alongside microtubule disruption, G2/M arrest, and induction of apoptosis.^20^ The benzo[*d*]imidazo[2,1-*b*]thiazole–chalcone hybrid II similarly demonstrated effective inhibition of tubulin assembly with consequent mitotic arrest.^21^ In addition, the thiazole-based chalcone III highlighted the intrinsic potential of this hybrid framework as a tubulin-targeting pharmacophore.^22^ This strategy was further substantiated by compounds IV and V, which combined tubulin interference with carbonic anhydrase IX inhibition.^23,24^ In another study, the bisthiazole chalcone VI exhibited pronounced antitubulin activity, with clear cell-cycle arrest and apoptotic effects in MCF-7 cells.^25^ Similarly, the thiazole-2-acetamide derivative VII displayed potent tubulin-associated antiproliferative activity accompanied by apoptosis induction.^26^ The structures of these representative compounds are shown in Fig. 1. Collectively, these findings reinforce the versatility of thiazole–chalcone hybrids as antimitotic agents.

A second pathway highly relevant to malignant progression is the cyclooxygenase pathway, particularly COX-2.^27^ COX-1 is constitutively expressed and is involved in homeostatic functions such as gastric mucosal protection and platelet physiology, whereas COX-2 is an inducible isoform that becomes markedly upregulated during inflammation and in many tumors.^28^ Beyond its role in inflammation, COX-2 contributes to several hallmarks of cancer by promoting prostaglandin-dependent proliferation, angiogenesis, migration, invasion, immune evasion, and resistance to apoptosis.^29^ These findings explain the continued interest in selective COX-2 inhibitors as anticancer agents.^29,30^ Classical coxibs such as celecoxib, rofecoxib, valdecoxib, and etoricoxib were developed to exploit structural differences between COX-1 and COX-2, particularly the larger secondary side pocket in COX-2. However, safety concerns have limited the use of some members of this class.^31,32^ Even so, the COX-2/PGE_2_ axis remains an attractive target for hybrid anticancer design.

**Fig. 2:**
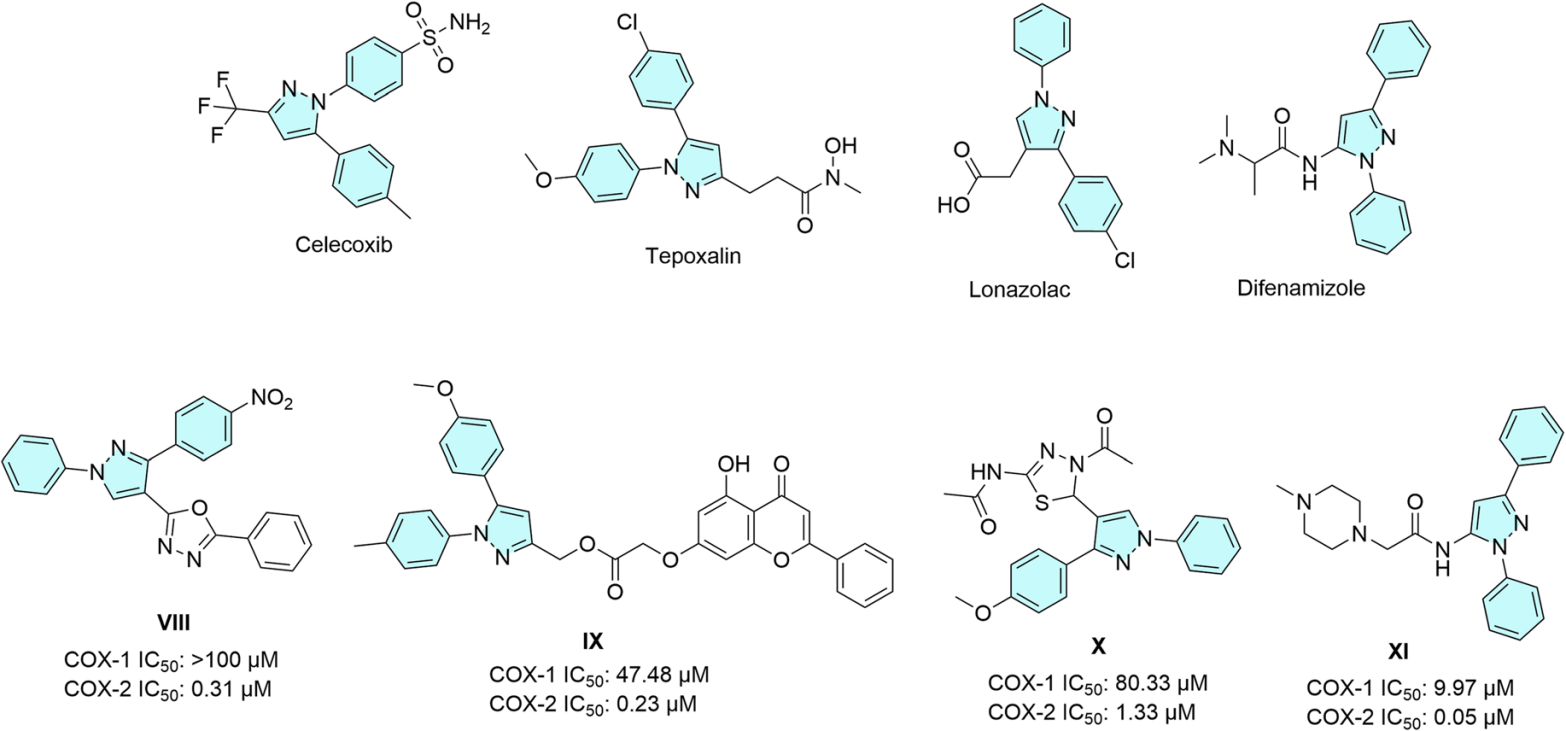
Selected pyrazole-containing drugs and pyrazole-based selective COX-2 inhibitors (VIII–XI).

The pyrazole ring is regarded as a privileged scaffold in medicinal chemistry because of its broad and well-documented spectrum of pharmacological activities, including analgesic, anti-inflammatory, anticancer, antimicrobial, and CNS effects.^33,34^ In particular, pyrazole-based compounds are well-established in NSAID chemistry and have been widely exploited in the design of COX-2-directed agents.^35^ Several marketed NSAID-related drugs also contain a pyrazole core, such as celecoxib, tepoxalin, lonazolac, and difenamizole.^36^ This importance is further underscored by several reports on pyrazole-based selective COX-2 inhibitors (Fig. 2). For example, pyrazole-1,3,4-oxadiazole hybrid VIII exhibited selective COX-2 inhibition, along with anti-inflammatory, analgesic, and gastro-sparing properties.^37^ Similarly, pyrazole–chrysin hybrid IX combined selective COX-2 inhibition with antiproliferative activity.^38^ Likewise, pyrazole–thiadiazole hybrid X emerged as a highly selective COX-2 inhibitor with a favorable drug-likeness profile,^39^ whereas more recent difenamizole-inspired pyrazole XI bearing a bulkier *N*-substituent showed improved COX-2 selectivity together with EGFR inhibition.^40^

### 1.1 Rational design

The simultaneous targeting of tubulin polymerization and COX-2 is biologically and pharmacologically justified. Tubulin inhibition disrupts mitotic spindle formation and promotes apoptotic cell death, whereas COX-2 inhibition is expected to suppress prostaglandin-driven signaling involved in tumor growth, angiogenesis, invasion, and resistance to apoptosis. This concept is supported by both preclinical and clinical evidence, including studies of celecoxib combined with taxane-based regimens.^41–43^ Moreover, the feasibility of integrating both activities within a single scaffold has already been demonstrated through molecular hybridization; notably, the rofecoxib/combretastatin hybrid KSS19 was designed to unite COX-2 inhibition with microtubule-disrupting activity while avoiding the *cis*–*trans* isomerization liability of CA-4, and later coxib–combretastatin hybrids likewise confirmed the potential of combining antiproliferative and anti-inflammatory properties within one molecule.

**Fig. 3:**
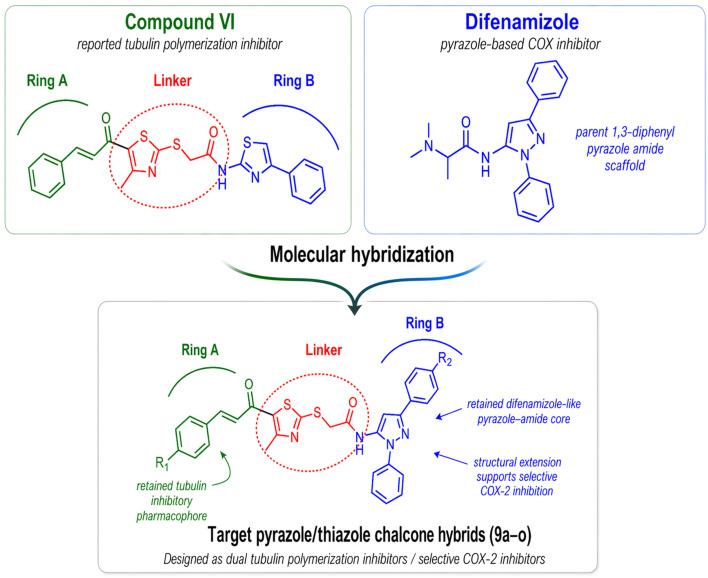
Rational design of the target hybrids 9a–o.

Guided by these considerations, the present design was developed to integrate tubulin polymerization inhibition and COX-2 targeting within a single hybrid molecule (Fig. 3). From the tubulin perspective, it was guided by the classical ring A-linker-ring B organization of CA-4-inspired colchicine-site inhibitors. In the current series, ring A consisted of a thiazole–chalcone fragment, selected as an extended hydrophobic pharmacophore capable of occupying the lipophilic region of the colchicine-binding pocket while also acting as a conformationally restricted replacement for the labile *cis*-stilbene system of CA-4. The linker incorporated a thioacetamide spacer, whereas ring B was introduced as a 1,3-diphenylpyrazole unit, chosen as a heteroaromatic moiety expected to establish favorable hydrophobic interactions within adjacent regions of the colchicine-binding site while also providing a COX-2-relevant structural element within the same framework.

More specifically, ring B was selected as a 1,3-diphenylpyrazole unit because the pyrazole nucleus represents the key heterocyclic motif of difenamizole, whereas the diphenyl substitution was expected to provide the steric expansion required for preferential accommodation within the larger COX-2 secondary pocket relative to COX-1. Applying this principle, the current design retained the 1,3-diphenylpyrazole–amide core while replacing the smaller dimethylamino substituent in difenamizole with a more extended thiazole–chalcone moiety. This modification was expected not only to favor binding to COX-2 but also to introduce a tubulin-directed pharmacophore. In addition, variation at the chalcone aryl ring and the 3-phenyl substituent of the pyrazole nucleus was intended to fine-tune hydrophobic and physicochemical features across both targets, thereby supporting the development of dual-acting anticancer candidates.

## 2. Results and discussion

### 2.1 Chemistry

**Scheme 1:**
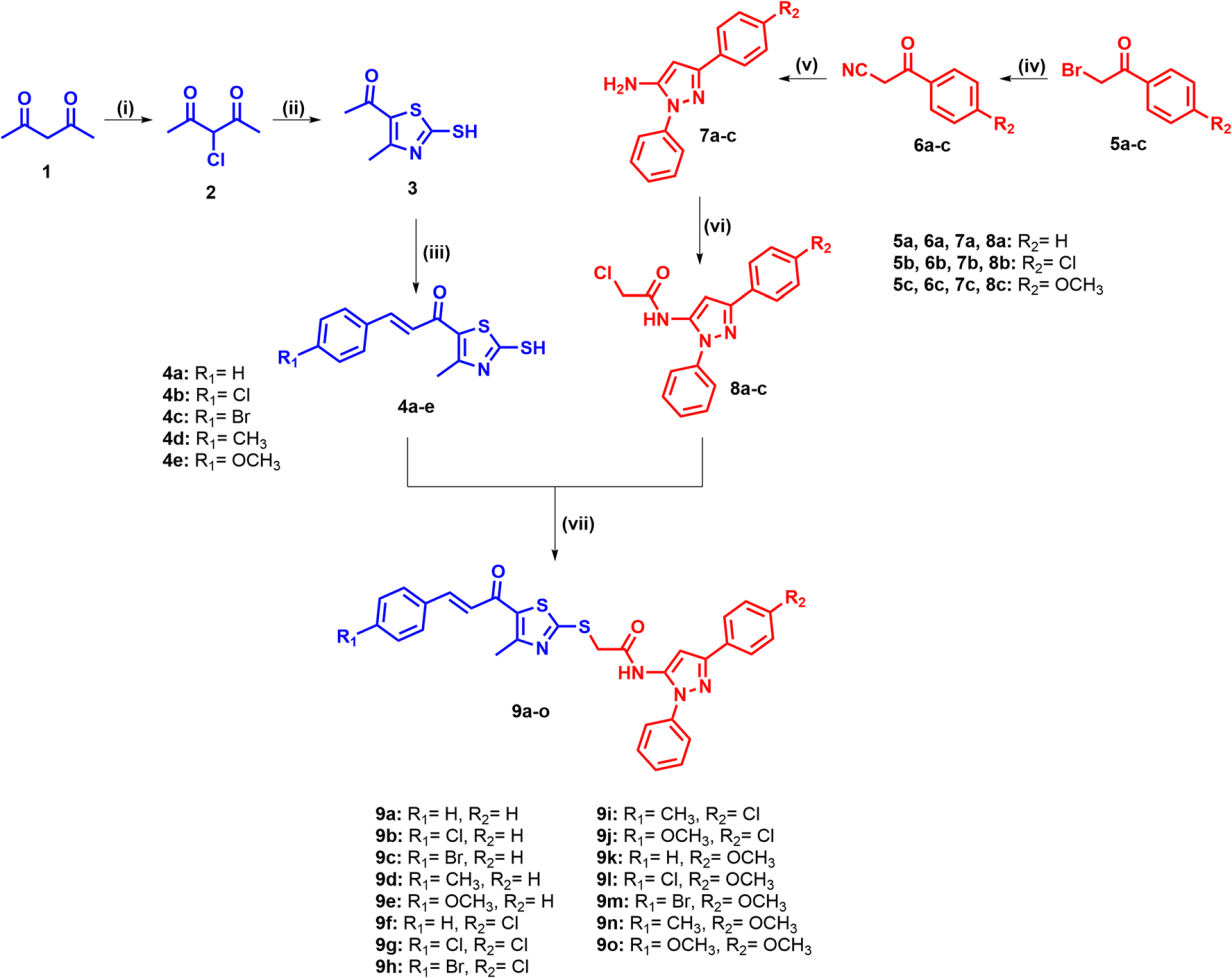
Synthesis of the thiazole chalcone/pyrazole hybrids 9a–o.

The target compounds 9a–o were synthesized as outlined in Scheme 1. Pentane-2,4-dione (1) underwent regioselective chlorination at the activated methylene position upon treatment with sulfuryl chloride in toluene at 0 °C for 12 h, affording 3-chloropentane-2,4-dione (2). Subsequent reaction of 2 with ammonia and carbon disulfide in ethanol at room temperature for 6 h generated the dithiocarbamate *in situ*, which cyclized to afford the 2-mercaptothiazole intermediate (3). Claisen–Schmidt condensation of 3 with the appropriate *para*-substituted benzaldehydes in ethanolic 60% NaOH at 0 °C for 18 h, then yielded the chalcone derivatives 4a–e bearing the free mercapto group at the thiazole C2 position.

In parallel, the pyrazole fragment was prepared from the corresponding phenacyl bromides 5a–c. Nucleophilic substitution of 5a–c with potassium cyanide in ethanol at 50 °C for 2 h afforded the β-ketonitriles 6a–c, which then underwent cyclocondensation with phenylhydrazine hydrochloride under reflux for 8 h to yield the amino pyrazoles 7a–c. *N*-acylation of the amino group in 7a–c was achieved with chloroacetyl chloride in dichloromethane at 0 °C for 12 h, giving the key chloroacetamido pyrazoles 8a–c. The final step involved *S*-alkylation of the mercaptochalcones 4a–e with the α-chloroacetamides 8a–c in acetonitrile containing triethylamine at room temperature for 6 h, affording the target compounds 9a–o.

### 2.2 Reagents and conditions

(i) SO_2_Cl_2_, toluene, 0 °C, 12 h; (ii) NH_3_, CS_2_, EtOH, RT, 6 h; (iii) appropriate benzaldehyde derivative, 60% NaOH, EtOH, 0 °C, 18 h; (iv) KCN/ethanol, 50 °C, 2 h; (v) phenylhydrazine/HCl, reflux, 8 h; (vi) chloroacetyl chloride, DCM, 0 °C, 12 h; (vii) acetonitrile, TEA, RT, 6 h.

The synthesized compounds 9a–o were characterized by ^1^H NMR, ^13^C NMR, and elemental analysis. The ^1^H NMR spectra, recorded in DMSO-*d*_6_, exhibited characteristic signals consistent with the proposed structures. A downfield broad singlet for the amide NH proton was observed at *δ* 10.45–10.56 ppm. The pyrazole C4-H resonated as a singlet between *δ* 6.82 and 6.94 ppm. The aromatic protons of the three phenyl rings appeared in the region *δ* 6.95–7.86 ppm. The *trans* chalcone olefinic protons were observed as two doublets at *δ* 7.62–7.68 ppm and *δ* 7.17–7.35 ppm, confirming the (*E*)-geometry of the enone system. The thioacetamide methylene protons appeared as a sharp singlet at *δ* 4.25–4.26 ppm, while the thiazole methyl group gave a singlet at *δ* 2.62–2.63 ppm. In derivatives containing a *p*-methyl substituent, an additional singlet was observed at *δ* 2.32 ppm. Methoxy groups on the aromatic rings (when present) appeared as singlets in the range of *δ* 3.75–3.79 ppm.

The ^13^C NMR spectra further supported the assigned structures, revealing two distinct carbonyl resonances: the chalcone ketone carbonyl at *δ* 181.71–181.97 ppm and the amide carbonyl at *δ* 168.21–168.98 ppm. The pyrazole C4 carbon appeared characteristically upfield at *δ* 99.31–99.99 ppm. The remaining aromatic carbons of the three phenyl rings and thiazole, along with the two olefinic chalcone carbons, resonated in the region *δ* 114.63–166.45 ppm. The thioacetamide methylene carbon was observed at *δ* 37.60–37.69 ppm, while the thiazole methyl carbon appeared at *δ* 18.72–18.84 ppm. When present, the *p*-methyl carbon resonated at *δ* 21.64–21.65 ppm and the methoxy carbons at *δ* 55.61–55.96 ppm. These spectral data collectively confirmed the successful formation of the target thiazole–pyrazole hybrids.

### 2.3 Biological evaluation

#### 2.3.1. Antiproliferative activity

The antiproliferative activity of the synthesized hybrids 9a–o was evaluated against three human cancer cell lines: MDA-MB-231 (triple-negative breast), HCA-7 (colon), and A549 (lung) carcinoma cell lines, with doxorubicin and combretastatin A-4 included as reference compounds (Table 1).

**Table 1:** Antiproliferative activity of compounds 9a–o against MDA-MB-231, HCA-7, and A549 cancer cell lines after 48 h treatment, expressed as IC_50_ values (µM) ± SEM, with doxorubicin and combretastatin A-4 as reference compounds

Compound	Antiproliferative activity IC_50_ ± SEM (µM)		
	MDA-MB-231	HCA-7	A549
9a	11.92 ± 0.62	15.84 ± 0.82	12.34 ± 0.64
9b	6.38 ± 0.33	8.76 ± 0.45	6.92 ± 0.36
9c	5.21 ± 0.27	7.42 ± 0.38	5.36 ± 0.28
9d	8.42 ± 0.43	10.48 ± 0.53	7.24 ± 0.37
9e	4.86 ± 0.25	3.28 ± 0.17	6.94 ± 0.35
9f	8.08 ± 0.41	6.54 ± 0.33	10.62 ± 0.54
9g	3.88 ± 0.20	4.92 ± 0.25	5.64 ± 0.29
9h	3.41 ± 0.17	4.36 ± 0.22	2.84 ± 0.14
9i	6.91 ± 0.35	5.86 ± 0.30	8.42 ± 0.43
9j	4.11 ± 0.21	3.14 ± 0.16	5.92 ± 0.30
9k	6.84 ± 0.35	5.12 ± 0.26	8.76 ± 0.44
9l	2.92 ± 0.15	2.18 ± 0.11	4.86 ± 0.25
9m	1.96 ± 0.10	2.57 ± 0.13	4.18 ± 0.21
9n	5.42 ± 0.27	4.26 ± 0.21	6.48 ± 0.33
9o	4.26 ± 0.21	3.02 ± 0.15	5.48 ± 0.28
Doxorubicin	1.62 ± 0.18	3.54 ± 0.36	4.96 ± 0.22
Combretastatin A-4	2.08 ± 0.27	1.91 ± 0.22	2.64 ± 0.24

The parent compound 9a, featuring unsubstituted phenyl rings at both positions (R_1_ = H, R_2_ = H), displayed the lowest activity within the series, exhibiting IC50 values of 11.92 ± 0.62, 15.84 ± 0.82, and 12.34 ± 0.64 µM against MDA-MB-231, HCA-7, and A549 cell lines, respectively. All substituted analogs showed improved activity relative to 9a, confirming that modifications at both aromatic positions enhance the anticancer activity. Among monosubstituted compounds (R_2_ = H), the potency order against MDA-MB-231 cells followed Br (9c, 5.21 ± 0.27 µM) > OCH_3_ (9e, 4.86 ± 0.25 µM) > Cl (9b, 6.38 ± 0.33 µM) > CH_3_ (9d, 8.42 ± 0.43 µM) > H (9a, 11.92 ± 0.62 µM). Statistical analysis of mean IC50 values by R_1_ substituent confirmed that bromo substitution yielded the lowest average IC50 (3.53 ± 0.18 µM against MDA-MB-231), followed by chloro (4.39 ± 0.23 µM) and methoxy (4.41 ± 0.24 µM), whereas unsubstituted (8.95 ± 0.48 µM) and methyl (6.92 ± 0.36 µM) derivatives exhibited reduced potency. Notably, compound 9e (R_1_ = OCH_3_, R_2_ = H) demonstrated activity against HCA-7 cells (3.28 ± 0.17 µM) that surpassed doxorubicin (3.54 ± 0.36 µM) against this cell line.

Methoxy substitution at R_2_ yielded the most potent compounds across all three cell lines, with disubstituted analogs showing superior activity to those bearing R_2_ = Cl or single substitutions. Notably, compounds with R_2_ = OCH_3_ exhibited the lowest mean IC50 against MDA-MB-231 (4.28 ± 0.23 µM), compared to R_2_ = Cl (5.28 ± 0.28 µM) and monosubstituted analogs (7.36 ± 0.40 µM). Compound 9m (R_1_ = Br, R_2_ = OCH_3_) emerged as the most potent compound overall in the series, displaying IC50 values of 1.96 ± 0.10, 2.57 ± 0.13, and 4.18 ± 0.21 µM against MDA-MB-231, HCA-7, and A549 cells, respectively. The activity of 9m against MDA-MB-231 cells represented an 83.6% improvement over the parent compound 9a and showed potency comparable to doxorubicin (1.62 ± 0.18 µM) and combretastatin A-4 (2.08 ± 0.27 µM). Compound 9l (R_1_ = Cl, R_2_ = OCH_3_) ranked as the second most potent analog overall, demonstrating IC50 values of 2.92 ± 0.15, 2.18 ± 0.11, and 4.86 ± 0.25 µM against MDA-MB-231, HCA-7, and A549, respectively, with activity against HCA-7 cells exceeding that of doxorubicin (3.54 ± 0.36 µM).

Compound 9h (R_1_ = Br, R_2_ = Cl) showed exceptional activity against A549 lung carcinoma cells (2.84 ± 0.14 µM) that exceeded its activity against the other two cell lines, indicating cell line-specific responses within the disubstituted series containing R_2_ = Cl. MDA-MB-231 triple-negative breast cancer cells generally demonstrated the highest sensitivity to the compound series, exhibiting the lowest mean IC50 values across most substitution patterns. HCA-7 colon cancer cells showed intermediate sensitivity, while A549 lung carcinoma cells displayed the most variable responses, with certain compounds (particularly 9h) showing exceptional activity while others demonstrated reduced potency.

#### 2.3.2. Cytotoxicity against normal MCF-10A breast epithelial cells

The cytotoxicity of the target compounds (9a–o) against normal MCF-10A breast epithelial cells was evaluated to assess selectivity toward cancer cells. Selectivity indices (SI) were calculated as the ratio of IC50 values for MCF-10A cells to those for each cancer cell line, with doxorubicin and combretastatin A-4 included as reference compounds (Table 2).

**Table 2:** Cytotoxicity of compounds 9a–o against normal MCF-10A breast epithelial cells after 48 h treatment, expressed as IC_50_ values (µM) ± SEM, together with their selectivity indices relative to MDA-MB-231, HCA-7, and A549 cancer cell lines; doxorubicin and combretastatin A-4 were included as reference compounds

Compound	MCF-10A IC_50_ ± SEM (µM)	Selectivity index (SI)		
		MDA-MB-231	HCA-7	A549
9a	24.24 ± 1.06	2.03	1.53	1.96
9b	19.31 ± 0.80	3.03	2.20	2.79
9c	16.94 ± 0.67	3.25	2.28	3.16
9d	20.34 ± 0.85	2.42	1.94	2.81
9e	18.92 ± 0.78	3.89	5.77	2.73
9f	21.86 ± 0.93	2.71	3.34	2.06
9g	15.43 ± 0.60	3.98	3.14	2.74
9h	13.78 ± 0.51	4.04	3.16	4.85
9i	17.94 ± 0.73	2.60	3.06	2.13
9j	16.74 ± 0.66	4.07	5.33	2.83
9k	20.88 ± 0.88	3.05	4.08	2.38
9l	14.92 ± 0.57	5.11	6.84	3.07
9m	12.76 ± 0.46	6.51	4.96	3.05
9n	18.08 ± 0.73	3.34	4.24	2.79
9o	16.02 ± 0.63	3.76	5.30	2.92
Doxorubicin	2.80 ± 0.25	1.73	0.79	0.56
Combretastatin A-4	4.80 ± 0.40	2.31	2.51	1.82

The target compounds exhibited substantially lower cytotoxicity against MCF-10A normal cells than against cancer cell lines, with IC50 values ranging from 12.76 ± 0.46 µM (9m) to 24.24 ± 1.06 µM (9a). Doxorubicin showed an IC50 of 2.80 ± 0.25 µM against MCF-10A cells, indicating considerable toxicity toward normal cells. The selectivity indices for compounds 9a–9o against MDA-MB-231 cells ranged from 2.03 (9a) to 6.51 (9m), with most analogs exceeding an SI value of 3.0. Doxorubicin demonstrated an SI of 1.73 against MDA-MB-231, while combretastatin A-4 showed an SI of 2.31. Against HCA-7 cells, doxorubicin exhibited an SI below 1 (0.79), indicating greater toxicity toward normal cells than this cancer cell line. All target compounds maintained SI values above 1.53, with compounds 9l (6.84) and 9e (5.77) showing favorable selectivity.

Compounds 9m and 9l, which demonstrated the highest antiproliferative potency against cancer cells, also exhibited the most favorable selectivity profiles. Compound 9m showed SI values of 6.51, 4.96, and 3.05 against MDA-MB-231, HCA-7, and A549 cells, respectively, while compound 9l displayed SI values of 5.11, 6.84, and 3.07 against the same cell lines. These values exceed those of doxorubicin across all three cancer cell lines and surpass those of combretastatin A-4 in MDA-MB-231 and A549 cells. Compound 9h (R_1_ = Br, R_2_ = Cl) also demonstrated strong selectivity, with an SI of 4.85 against A549 cells, consistent with its activity against this cell line observed in the antiproliferative assay.

The selectivity indices against A549 lung carcinoma cells were generally lower than those observed for MDA-MB-231 and HCA-7 cells across the compound series, with values ranging from 1.96 (9a) to 4.85 (9h) compared to combretastatin A-4 (1.82) and doxorubicin (0.56). Compounds 9m and 9l achieved SI values above 3.0 against A549 cells, indicating acceptable selectivity margins for this cell line.

#### 2.3.3. *In vitro* tubulin polymerization inhibitory assay

**Fig. 4:**
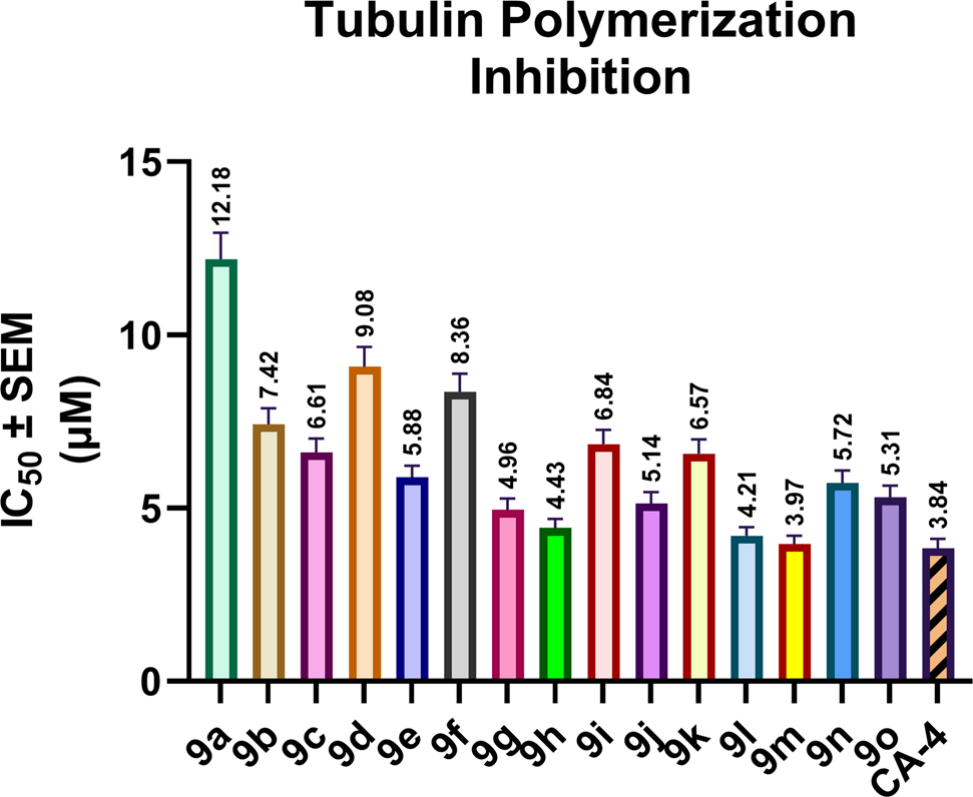
Tubulin polymerization inhibitory activity of compounds 9a–o and the reference inhibitor CA-4, expressed as IC_50_ values (µM), as determined from a fluorescence-based polymerization assay monitored kinetically for 60 min at 37 °C. Data are presented as mean ± SEM.

The effects of the synthesized hybrids 9a–o on tubulin polymerization, using combretastatin A-4 (CA-4) as the reference inhibitor, are illustrated in Fig. 4. Compounds 9a–o exhibited clear inhibitory activity against tubulin polymerization, with IC_50_ values ranging from 3.97 to 12.18 µM, compared to CA-4 (IC_50_ = 3.84 ± 0.28 µM). Among the tested derivatives, compounds 9m, 9l, 9h, and 9g were the most active, with IC_50_ values of 3.97 ± 0.24, 4.21 ± 0.25, 4.43 ± 0.27, and 4.96 ± 0.31 µM, respectively. In particular, 9m exhibited the highest tubulin polymerization-inhibitory activity in the series and was only 1.03-fold less potent than CA-4, whereas 9l and 9h were 1.10-fold and 1.15-fold less potent than the reference compound, respectively.

The anti-tubulin activity of compounds 9a–o was markedly affected by the substitution pattern at both R_1_ and R_2_. When R_2_ was kept unsubstituted, modification of the chalcone phenyl ring (R_1_) produced a substantial enhancement in activity relative to the parent derivative 9a (IC_50_ = 12.18 ± 0.76 µM). Within this subset, compound 9e (R_1_ = OCH_3_, R_2_ = H) was the most active analogue, with an IC_50_ value of 5.88 ± 0.34 µM, followed by 9c (R_1_ = Br, R_2_ = H; 6.61 ± 0.39 µM), 9b (R_1_ = Cl, R_2_ = H; 7.42 ± 0.46 µM), and 9d (R_1_ = CH_3_, R_2_ = H; 9.08 ± 0.57 µM). Thus, when the pyrazole-linked phenyl ring remained unsubstituted, activity increased in the order H < CH_3_ < Cl < Br < OCH_3_.

A different but related trend was observed upon substitution at R_2_ with chlorine. Compounds 9f–j, bearing R_2_ = Cl, showed improved activity relative to their corresponding R_2_ = H analogs. For example, the introduction of a chlorine substituent reduced the IC_50_ value from 12.18 to 8.36 µM for 9a/9f, from 7.42 to 4.96 µM for 9b/9g, from 6.61 to 4.43 µM for 9c/9h, from 9.08 to 6.84 µM for 9d/9i, and from 5.88 to 5.14 µM for 9e/9j. Within this subgroup, activity followed the order H < CH_3_ < OCH_3_ < Cl < Br, with 9h (R_1_ = Br) and 9g (R_1_ = Cl) representing the most active derivatives.

The most favorable profile was obtained when R_2_ carried a methoxy group. Compounds 9k–o, bearing R_2_ = OCH_3_, constituted the most active subgroup overall, with IC_50_ values ranging from 3.97 to 6.57 µM. Within this subset, compound 9m (R_1_ = Br) was the most potent derivative in the entire series, followed by 9l (R_1_ = Cl), 9o (R_1_ = OCH_3_), 9n (R_1_ = CH_3_), and 9k (R_1_ = H). Direct comparison with the corresponding R_2_ = H analogs further demonstrated the favorable contribution of methoxy substitution at R_2_. Thus, 9k was 1.85-fold more potent than 9a, 9l was 1.76-fold more potent than 9b, 9m was 1.67-fold more potent than 9c, 9n was 1.59-fold more potent than 9d, and 9o was slightly more active than 9e.

The tubulin polymerization results were also in good agreement with the antiproliferative data obtained against MDA-MB-231, HCA-7, and A549 cells. Compounds 9m and 9l, which displayed the highest tubulin polymerization inhibitory activity, were likewise the most potent antiproliferative derivatives in the cellular assay. In contrast, the weaker tubulin polymerization inhibitor 9a displayed the lowest antiproliferative activity within the series. This parallel behavior supports the conclusion that inhibition of tubulin polymerization is a major contributor to the cytotoxic activity of these hybrids.

#### 2.3.4. *In vitro* COX inhibition assay

**Fig. 5:**
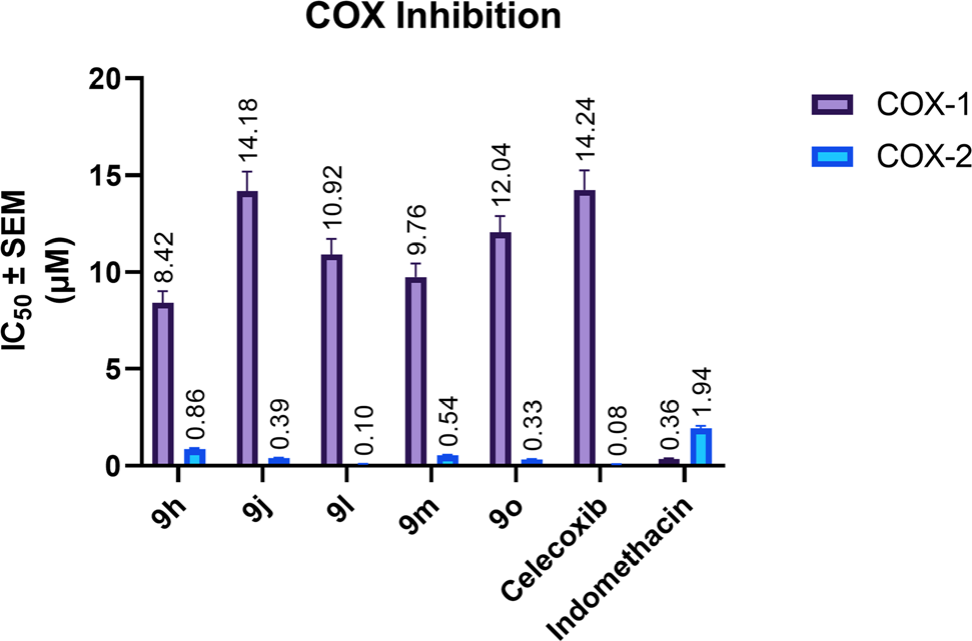
COX-1 and COX-2 inhibitory activities of compounds 9h, 9j, 9l, 9m, and 9o, compared with celecoxib and indomethacin, expressed as IC_50_ values (µM), obtained after 10 min compound/enzyme preincubation at 37 °C, 2 min arachidonic-acid reaction, and 15 min post-SnCl_2_ incubation. Data are presented as mean ± SEM.

The five most promising compounds, namely 9h, 9j, 9l, 9m, and 9o, were selected for further evaluation of their inhibitory activity against COX-1 and COX-2, based on their superior antiproliferative activity and potent inhibition of tubulin polymerization. The obtained COX-1/COX-2 inhibitory profiles are illustrated in Fig. 5, while the corresponding selectivity indices are presented in Table 3. All the tested hybrids demonstrated preferential inhibition of COX-2 over COX-1, with COX-2 IC_50_ values spanning a submicromolar range (0.10–0.86 µM) and selectivity indices ranging from 9.79 to 109.20. In contrast, indomethacin exhibited a COX-2 SI of only 0.19, reflecting its well-established preferential inhibition of the constitutive COX-1 isoform.

**Table 3:** COX-2 selectivity indices of selected compounds (9h, 9j, 9l, 9m, and 9o) compared with reference drugs (celecoxib and indomethacin), calculated from IC_50_ values obtained after 10 min compound/enzyme preincubation at 37 °C, 2 min arachidonic-acid reaction, and 15 min post-SnCl_2_ incubation

Compound	COX-2 selectivity index
9h	9.79
9j	36.36
9l	109.20
9m	18.07
9o	36.48
Celecoxib	178.00
Indomethacin	0.19

Among the tested derivatives, compound 9l (R_1_ = Cl, R_2_ = OCH_3_) exhibited the most remarkable COX-2 inhibitory profile, with an IC_50_ value of 0.10 ± 0.01 µM that was comparable to that of the clinically approved selective COX-2 inhibitor celecoxib (IC_50_ = 0.08 ± 0.01 µM), being only 1.25-fold less potent than the reference drug. Furthermore, 9l displayed a COX-2 selectivity index of 109.20, which represents approximately 61% of celecoxib's SI (178.00) and is notably exceptional for a non-coxib scaffold devoid of the sulfonamide or methylsulfonyl pharmacophore that is classically considered essential for high COX-2 selectivity. This observation suggests that the chloro substituent on the chalcone phenyl ring, in conjunction with the methoxy group on the pyrazole-linked phenyl ring, provides a highly favorable substitution pattern for selective COX-2 inhibition. The relatively high COX-1 IC_50_ of 9l (10.92 ± 0.78 µM) further contributes to its pronounced selectivity profile.

The remaining derivatives also demonstrated appreciable COX-2 inhibitory activity, with IC_50_ values of 0.33 ± 0.03, 0.39 ± 0.03, 0.54 ± 0.04, and 0.86 ± 0.06 µM for compounds 9o, 9j, 9m, and 9h, respectively. The overall COX-2 potency within the evaluated subset followed the order 9l > 9o > 9j > 9m > 9h, while the corresponding selectivity indices decreased in the same order: 109.20, 36.48, 36.36, 18.07, and 9.79. All five compounds surpassed the COX-2 potency of indomethacin (IC_50_ = 1.94 ± 0.14 µM) by margins ranging from 2.3-fold (9h) to 19.4-fold (9l), and all exhibited substantially superior selectivity profiles relative to the non-selective reference.

#### 2.3.5. Cell cycle analysis and apoptosis

**Fig. 6:**
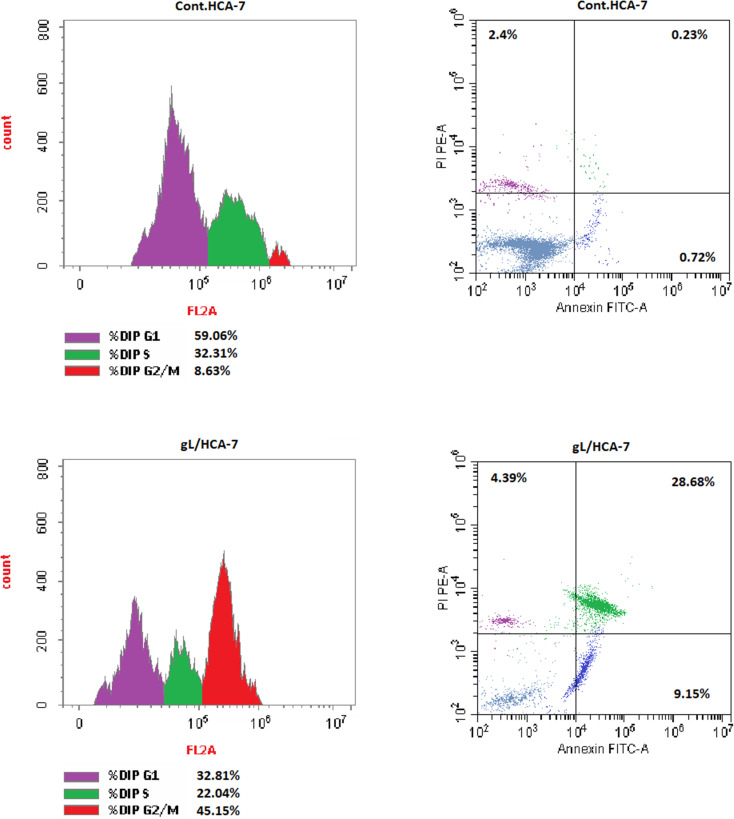
Effect of compound 9l on cell-cycle progression and apoptosis in HCA-7 cells after 48 h treatment at its IC_50_ concentration. Left panels show PI-based cell-cycle distribution, while right panels represent Annexin V-FITC/PI analysis of apoptotic populations.

Flow-cytometric analysis of PI-stained HCA-7 cells showed that treatment with 9l markedly altered cell-cycle distribution. The cell-cycle data are illustrated in Fig. 6 and summarized in Table 4. Compared with the untreated control, 9l reduced the G0/G1 population from 59.06% to 32.81% and the S-phase fraction from 32.31% to 22.04%, while markedly increasing the G2/M population from 8.63% to 45.15%. This pronounced accumulation of cells at the G2/M phase indicates that 9l induced cell-cycle arrest at this stage. Such a profile is consistent with the anti-mitotic behavior expected for tubulin-targeting agents. It also supports the tubulin polymerization data, indicating that disruption of mitotic progression is a major contributor to the antiproliferative effect of 9l in HCA-7 cells.

**Table 4:** Effect of compound 9l on cell-cycle distribution in HCA-7 cells after 48 h treatment at its IC_50_ concentration, expressed as percentages of cells in G0/G1, S, and G2/M phases

Compound no.	DNA content		
	% G0–G1	% S	% G2/M
Compound 9l	32.81	22.04	45.15
DMSO (control)	59.06	32.31	8.63

Annexin V-FITC/PI double staining further demonstrated that the growth-inhibitory effect of 9l was primarily mediated through induction of apoptotic cell death. The apoptotic distributions of treated and untreated cells are shown in Fig. 6 and summarized in Table 5. Treatment with 9l increased the early and late apoptotic populations to 9.15% and 28.68%, respectively, whereas the necrotic population remained limited at 4.39%. In contrast, the untreated control showed only 0.72% early apoptosis, 0.23% late apoptosis, and 2.40% necrosis. The marked elevation in the apoptotic fractions, together with the predominance of late apoptosis over early apoptosis, indicates efficient progression of HCA-7 cells toward irreversible apoptosis rather than nonspecific cytotoxic injury.

**Table 5:** Effect of compound 9l on apoptosis in HCA-7 cells after 48 h treatment at its IC_50_ concentration, expressed as percentages of total apoptotic, early apoptotic, late apoptotic, and necrotic cell populations

Compound no.	Apoptosis			Necrosis
	Total	Early	Late	
Compound 9l	42.22	9.15	28.68	4.39
DMSO (control)	3.35	0.72	0.23	2.4

#### 2.3.6. Effect of compound 9l on Bax and Bcl-2 protein expression

The effect of compound 9l on the expression levels of the pro-apoptotic protein Bax and the anti-apoptotic protein Bcl-2 was assessed in HCA-7 cells (Table 6). Bax and Bcl-2 are key regulators of apoptosis, with Bax promoting and Bcl-2 inhibiting cell death. Accordingly, the Bax/Bcl-2 ratio represents a critical determinant of cellular fate, particularly in cancer cells where apoptotic pathways are frequently dysregulated.

**Table 6:** Effect of compound 9l on Bax and Bcl-2 protein expression levels in HCA-7 cells after 48 h treatment at its IC_50_ concentration, relative to the DMSO-treated control

Compound	Bax (pg µL^−1^) ± SEM	Fold change	Bcl-2 (pg µL^−1^) ± SEM	Fold change
Compound 9l	438.64 ± 15.72	5.63	6.74 ± 0.19	0.332
DMSO (control)	77.94 ± 2.08	1.00	20.32 ± 0.46	1.00

The results indicated a significant increase in Bax protein levels following treatment with compound 9l, with a concentration of 438.64 ± 15.72 pg mL^−1^, representing a 5.63-fold increase compared to the DMSO control. This finding aligns with Bax's role as a pro-apoptotic factor that triggers the intrinsic apoptosis pathway by promoting mitochondrial cytochrome c release, ultimately leading to caspase activation and cell death. The upregulation of Bax suggests that compound 9l may induce apoptosis *via* mitochondrial pathways, a desirable feature for anticancer agents aiming to selectively eliminate tumor cells.

Conversely, compound 9l caused a marked reduction in Bcl-2 levels, with a concentration of 6.74 ± 0.19 pg mL^−1^, indicating a 0.332-fold change relative to the control. Bcl-2 is known to prevent apoptosis by inhibiting the release of cytochrome c from mitochondria and blocking caspase activation. The observed downregulation of Bcl-2 by compound 9l could further enhance the pro-apoptotic environment in treated cells, as decreased Bcl-2 levels would diminish its antagonistic effect on Bax, thereby facilitating apoptosis.

#### 2.3.7. Effect of compound 9l on caspase-3 and caspase-9 levels

The impact of compound 9l on apoptotic signaling was assessed through caspase-3 and caspase-9 assays, providing further evidence of its potential as a dual tubulin/COX-2 inhibitor with pronounced pro-apoptotic activity (Table 7). Caspase-9 acts as an initiator caspase, while caspase-3 functions as an executioner within the apoptotic cascade, and the marked elevation of both enzymes reflects activation of the intrinsic apoptotic pathway.

**Table 7:** Effect of compound 9l on caspase-3 and caspase-9 levels in HCA-7 cells after 48 h treatment at its IC_50_ concentration, relative to the DMSO-treated control

Compound	Caspase-3 (pg mL^−1^) ± SEM	Fold change	Caspase-9 (ng mL^−1^) ± SEM	Fold change
Compound 9l	496.80 ± 14.90	8.54	47.86 ± 1.18	9.08
DMSO (control)	58.16 ± 1.62	1.00	5.27 ± 0.23	1.00

In HCA-7 cells, treatment with compound 9l resulted in a substantial increase in caspase-3 levels to 496.80 ± 14.90 pg mL^−1^, corresponding to an 8.54-fold rise relative to the DMSO control. This elevation confirms progression toward the execution phase of apoptosis and highlights the compound's ability to promote programmed cell death in cancer cells.

Consistently, caspase-9 levels increased to 47.86 ± 1.18 ng mL^−1^ (9.08-fold *vs.* control), indicating activation of the mitochondrial apoptotic pathway. This upregulation is associated with mitochondrial perturbation, leading to cytochrome c release, apoptosome formation, and subsequent activation of the caspase cascade. Collectively, these findings confirm that compound 9l effectively induces apoptosis *via* the intrinsic mitochondrial pathway.

#### 2.3.8. Effect of compound 9l on PGE-2 production

The effect of compound 9l on prostaglandin E2 (PGE-2) production was evaluated by quantifying its extracellular levels in HCA-7 cell culture supernatants using a competitive ELISA assay. PGE-2 is a key downstream mediator of cyclooxygenase-2 activity and plays a central role in tumor progression through its involvement in proliferation, angiogenesis, and resistance to apoptosis. The corresponding results are presented in Table 8.

**Table 8:** Effect of compound 9l on extracellular PGE-2 production in HCA-7 cells after 24 h treatment with compound 9l (1.0 µM), relative to the untreated control

Compound	PGE-2 level (ng mL^−1^)	Fold change	Relative PGE-2 production	Inhibition of PGE-2 release (%)
Untreated control	5.16 ± 0.18	1.00	100.0	0.0
Compound 9l	0.56 ± 0.04	0.11	10.8	89.2

Treatment with compound 9l at 1.0 µM resulted in a pronounced suppression of PGE-2 release relative to the untreated control. In HCA-7 cells, 9l reduced extracellular PGE-2 levels to 0.56 ± 0.04 ng mL^−1^, corresponding to a 0.11-fold change and an 89.2% decrease compared to the control (5.16 ± 0.18 ng mL^−1^). This substantial reduction demonstrates the ability of 9l to effectively attenuate prostaglandin biosynthesis in intact cancer cells. This pronounced suppression is consistent with the previously established selective COX-2 inhibitory profile of 9l and indicates effective inhibition of COX-2-mediated signaling in a cellular context. Moreover, the marked reduction in PGE-2 complements the observed antiproliferative and pro-apoptotic effects, further supporting the multitarget anticancer potential of compound 9l.

#### 2.3.9. Effect of compound 9l on cell migration

The migratory response of HCA-7 cells to 9l was further examined using the CytoSelect™ wound-healing assay, in which removal of the insert generates a defined 0.9 mm wound field that enables direct monitoring of cell migration into the gap over time. This assay is particularly relevant because reduced wound closure reflects impaired collective cell migration, a process closely linked to tumor invasion and metastatic dissemination.

**Fig. 7:**
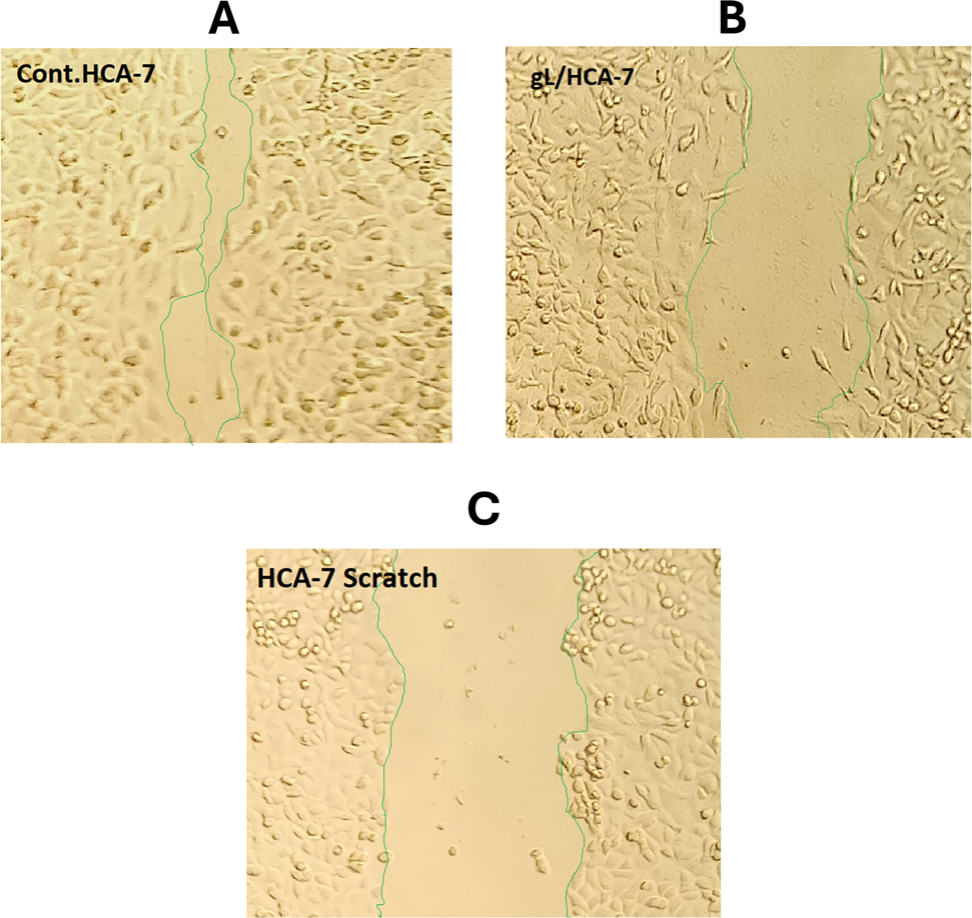
Wound-healing images of HCA-7 cells after 72 h: (A) control, (B) 9l-treated, and (C) initial wound field (0 h). Green lines indicate wound edges.

**Fig. 8:**
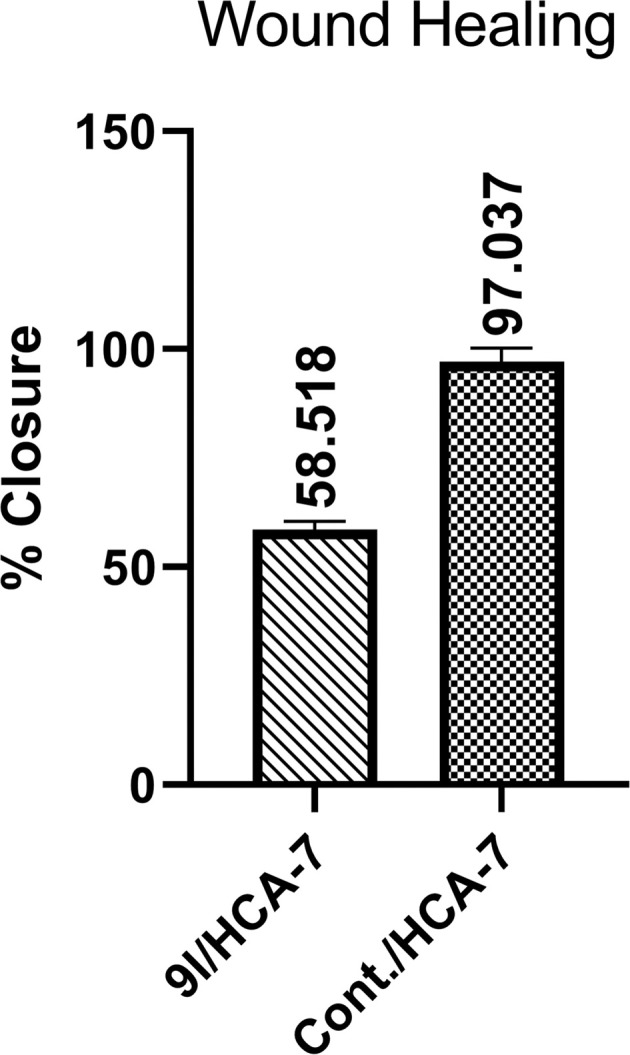
Wound closure (%) of HCA-7 cells after 72 h treatment with 9l*versus* control.

As shown in Fig. 7, the results further demonstrated that 9l significantly impaired the migratory capacity of HCA-7 cells. Compared with the untreated control, which showed almost complete wound closure after 72 h (97.04 ± 3.15%), treatment with 9l reduced wound closure to 58.52 ± 1.88% (Fig. 8). This reduction was also reflected in the migrated cell area, which decreased from 0.786 in the control to 0.474 in the treated group, together with a clear reduction in migration length (Δ*L* = 0.4367 mm for control *vs.* 0.2633 mm for 9l-treated cells). These findings indicate that 9l markedly suppresses HCA-7 cell migration, further extending its anticancer profile beyond growth inhibition and apoptosis induction.

#### 2.3.10. Metabolic stability of compound 9l in human liver microsomes

To assess its metabolic liability, the microsomal stability of compound 9l was evaluated using pooled human liver microsomes under NADPH-supported oxidative conditions at substrate concentrations of 0.8, 4.0, and 20.0 µM. The obtained data are summarized in Table 9.

**Table 9:** Microsomal stability parameters of compound 9l in pooled human liver microsomes at different starting concentrations, expressed as *t*_1/2_ (h), intrinsic clearance (CLint; µL min^−1^ mg^−1^), and percentage of parent compound remaining at 60 min

Start conc. (µM)	*t*_1/2_ (h)	CLint (µL min^−1^ mg^−1^)	% Remaining at 60 min
0.8	0.542 ± 0.032	42.69 ± 2.55	28.0 ± 0.7
4.0	0.873 ± 0.013	26.46 ± 0.40	50.3 ± 1.2
20.0	1.321 ± 0.013	17.49 ± 0.18	61.0 ± 0.2

The results revealed a clear concentration-dependent improvement in apparent stability across the tested range. Increasing the starting concentration from 0.8 to 4.0 and 20.0 µM was associated with progressive prolongation of the half-life from 0.542 ± 0.032 h (32.5 ± 1.9 min) to 0.873 ± 0.013 h (52.4 ± 0.8 min) and 1.321 ± 0.013 h (79.3 ± 0.8 min), respectively. In parallel, the apparent intrinsic clearance decreased from 42.69 ± 2.55 to 26.46 ± 0.40 and 17.49 ± 0.18 µL min^−1^ mg^−1^, while the percentage of parent compound remaining at 60 min increased from 28.0 ± 0.7% to 50.3 ± 1.2% and 61.0 ± 0.2%. This moderate-to-good profile, which improves at higher substrate levels consistent with possible enzyme saturation, falls within acceptable drug-like ranges and supports its suitability as a promising anticancer lead with acceptable metabolic stability under the tested *in vitro* conditions.

### 2.4 Computational studies

#### 2.4.1. Molecular docking

##### 2.4.1.1. Docking into the colchicine-binding site of tubulin

**Fig. 9:**
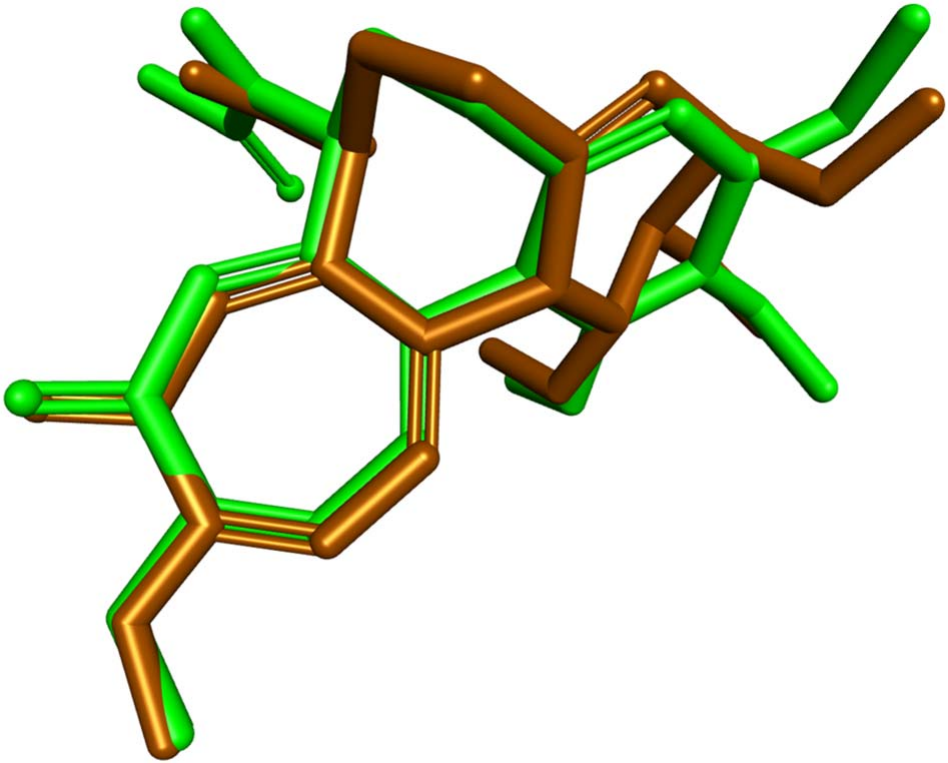
Superimposition of the co-crystallized (green) and redocked (brown) colchicine poses within the colchicine-binding site of tubulin (PDB ID: 4O2B).

To clarify the molecular basis of the potent anti-tubulin activity of compound 9l, docking studies were performed within the colchicine-binding site of tubulin (PDB ID: 4O2B).^44^ The docking protocol was first validated by redocking the co-crystallized ligand colchicine into its native binding pocket. Superimposition of the redocked and crystallographic poses gave an RMSD value of 0.7508 Å, confirming the reliability of the adopted docking procedure. The predicted binding affinity of colchicine was −8.4 kcal mol^−1^. The validation result is shown in Fig. 9.

Docking of compound 9l into the same binding cavity afforded a predicted binding affinity of −9.6 kcal mol^−1^, indicating a theoretically more favorable interaction than colchicine. Examination of the best-ranked pose revealed that 9l was well accommodated within the colchicine pocket and formed multiple stabilizing interactions, predominantly hydrophobic in nature. The pyrazole ring established π–sigma interaction with Leu255, π–alkyl interaction with Leu242, and π–lone pair interaction with Val238. The two pyrazole-linked phenyl rings further reinforced binding through several hydrophobic contacts with Ala250, Cys241, Leu242, Leu255, Val238, Thr239, and Leu252, indicating efficient occupation of the hydrophobic region of the binding site.

**Fig. 10:**
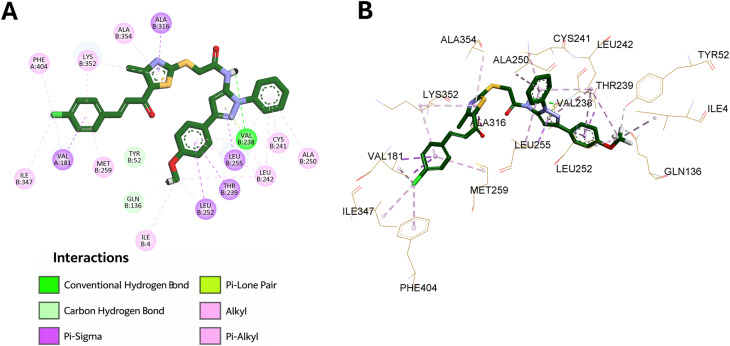
Binding interactions of compound 9l within the colchicine-binding site of tubulin (PDB ID: 4O2B): (A) 2D interaction diagram and (B) 3D binding mode.

Additional stabilization was provided by the methoxy substituent on the pyrazole-linked phenyl ring, which formed hydrophobic contacts with Leu242, Leu252, and Ile4, together with carbon–hydrogen bond interactions with Tyr52 and Gln136. Notably, the amidic NH formed a classical hydrogen bond with Val238, which may play an important role in anchoring the ligand within the pocket. The thiazole ring also contributed to binding through hydrophobic interactions with Ala316, Ala354, and Lys352, while the chalcone phenyl ring interacted with Lys352, Met259, and Val181. Moreover, the chloro substituent on the chalcone phenyl ring formed additional lipophilic contacts with Phe404, Ile347, and Val181. These results suggest that 9l binds favorably within the colchicine site, consistent with its potent tubulin polymerization inhibitory and antiproliferative activities. The detailed binding interactions are shown in Fig. 10.

##### 2.4.1.2. Docking into COX-2 enzyme

**Fig. 11:**
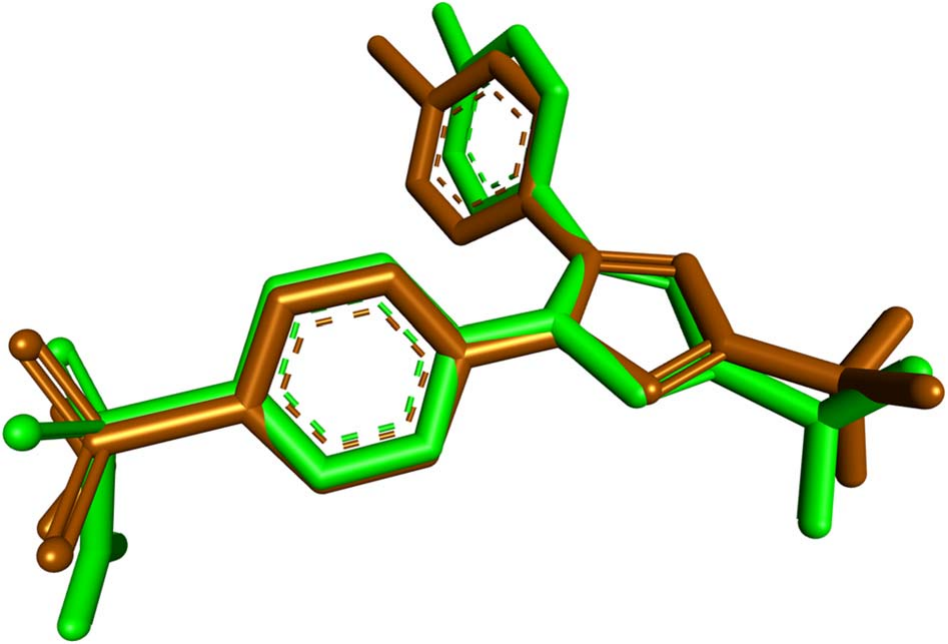
Superimposition of the co-crystallized (green) and redocked (brown) SC-558 poses within the active site of COX-2 (PDB ID: 1CX2).

To investigate the second intended pharmacological target, compound 9l was docked into the active site of COX-2 (PDB ID: 1CX2).^31^ The docking protocol was validated by redocking the co-crystallized ligand SC-558 into its native binding site. Superimposition of the redocked and crystallographic poses gave an RMSD value of 0.8631 Å, confirming the validity of the protocol for this target. The predicted binding affinity of SC-558 was −10.3 kcal mol^−1^. The superimposition result is presented in Fig. 11.

Compound 9l exhibited a predicted binding affinity of −11.0 kcal mol^−1^, suggesting a theoretically favorable interaction within the COX-2 active site. Analysis of the docked pose revealed that the pyrazole ring formed π–sigma interaction with Val349 and π–alkyl interaction with Ala527, while the two pyrazole-linked phenyl rings established multiple hydrophobic contacts with Val523, Ala527, Val349, Val116, Leu359, and Leu531. Additional stabilization arose from an amide–π stacked interaction with Gly526 and a π-donor non-classical hydrogen bond with Arg120.

**Fig. 12:**
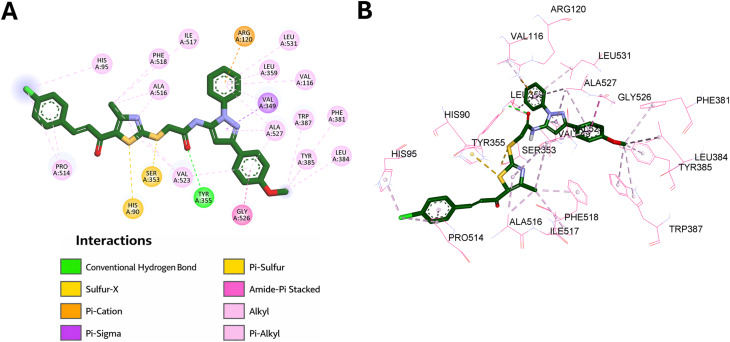
Binding interactions of compound 9l within the active site of COX-2 (PDB ID: 1CX2): (A) 2D interaction diagram and (B) 3D binding mode.

The methoxy substituent on the pyrazole-linked phenyl ring further enhanced binding through hydrophobic interactions with Leu384, Tyr385, Phe381, and Trp387. In addition, the amidic carbonyl formed a classical hydrogen bond with Tyr355, which likely contributes to stabilization of the docked complex. The sulfur atom of the linker showed a sulfur–X interaction with Ser353, whereas the thiazole ring and its methyl substituent participated in several hydrophobic interactions with Ala516, Val523, Ile517, and Phe518. Moreover, the thiazole sulfur formed a π–sulfur interaction with His90. At the distal end, the chalcone phenyl ring interacted with Pro514, while its chloro substituent formed additional hydrophobic contacts with His95 and Pro514. These findings indicate that 9l is well accommodated within the COX-2 active site, in agreement with its strong and selective COX-2 inhibitory activity. The interaction pattern of 9l in the COX-2 active site is presented in Fig. 12.

##### 2.4.1.3. *In Silico* ADMET prediction

The pharmacokinetic and safety-related properties of compound 9l were preliminarily evaluated using the ADMETlab 3.0 platform.^45^ The predicted profile revealed several favorable features supporting its potential as a bioactive lead. Compound 9l showed a molecular weight of 599.11, TPSA of 74.08 Å^2^, 6 hydrogen-bond acceptors, no hydrogen-bond donors, and 11 rotatable bonds, indicating acceptable polarity and flexibility. Although its lipophilicity was relatively high (log *P* = 6.03) and aqueous solubility was low (log *S* = −7.38), these features are consistent with the highly aromatic scaffold and may favor hydrophobic target binding.

**Fig. 13:**
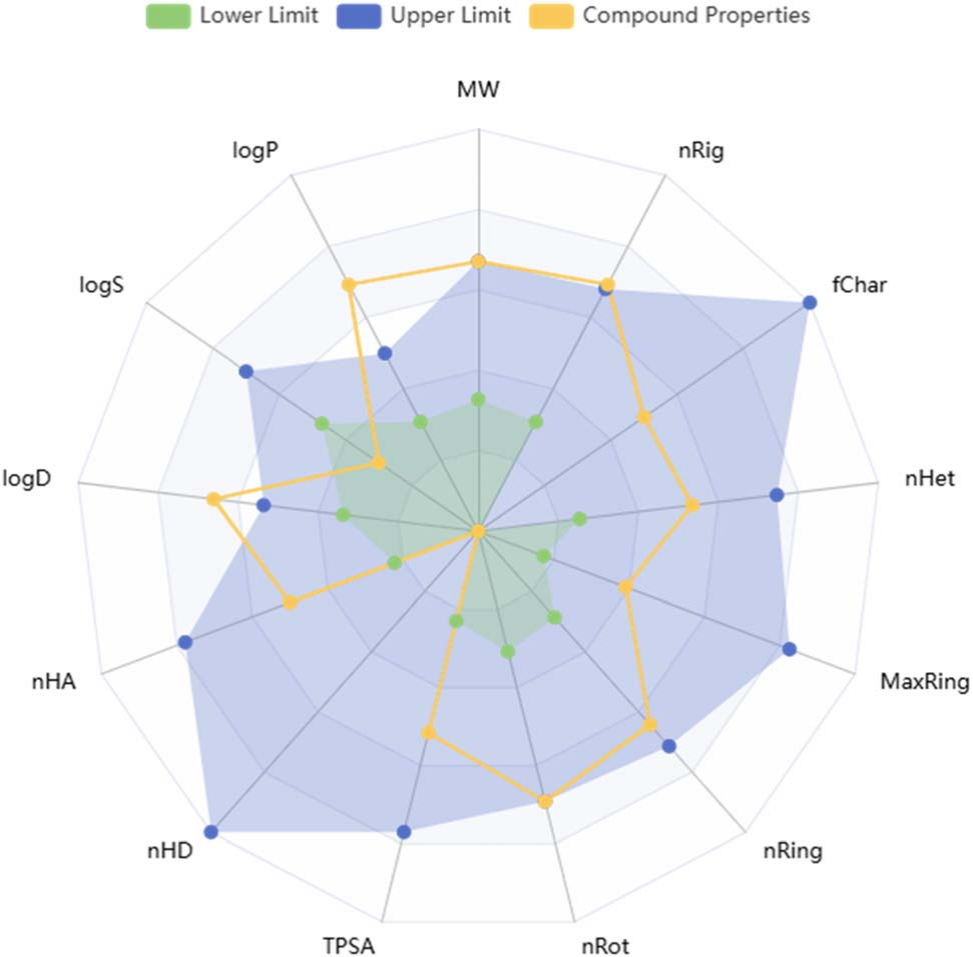
ADMET radar plot of compound 9l generated using ADMETlab 3.0, showing key physicochemical properties relative to the optimal drug-likeness space.

This trend was also reflected in the ADMET radar plot (Fig. 13), where most properties were positioned within or close to the recommended physicochemical space, with the main deviations related to lipophilicity and distribution coefficient. Compound 9l also showed no PAINS, BMS, or chelator alerts, together with very low probabilities of reactivity (0.02) and promiscuity (0.006), and remained synthetically accessible (Synth = 2).

Absorption-related prediction was encouraging, with a low probability for poor intestinal absorption (HIA = 0.00), favorable oral bioavailability-related outputs (F20% = 0.00, F30% = 0.037, and F50% = 0.064), and non-substrate behavior toward *P*-glycoprotein (*P*-gp substrate = 0.00), although compound 9l was predicted to act as a *P*-gp inhibitor (1.00). In addition, compound 9l showed very low predicted BBB penetration (0.067), acceptable VDss (0.174 L kg^−1^), a relatively high unbound fraction (Fu = 0.438), low probability of microsomal instability (0.256), and low predicted plasma clearance (2.571 mL min^−1^ kg^−1^), despite a short predicted half-life (0.914 h).

On the toxicity side, several favorable predictions were obtained, including low probabilities for Ames mutagenicity (0.215), rat oral acute toxicity (0.163), eye corrosion (0.00), eye irritation (0.031), hematotoxicity (0.187), RPMI-8226 immunotoxicity (0.029), and A549 cytotoxicity (0.154). Although alerts were predicted for hERG liability and hepatotoxicity, these findings require experimental confirmation. Overall, the ADMETlab 3.0 results support compound 9l as a synthetically feasible and pharmacologically promising lead.

## 3. Conclusion

In conclusion, a new series of thiazole–chalcone/pyrazole hybrids (9a–o) was successfully designed, synthesized, and biologically evaluated as potential dual-acting anticancer agents targeting tubulin polymerization and COX-2. Several derivatives exhibited promising antiproliferative activity against MDA-MB-231, HCA-7, and A549 cancer cell lines, with improved selectivity over normal MCF-10A cells. Compounds 9m and 9l emerged as the most active members of the series. Among them, compound 9l showed the most balanced biological profile, displaying potent tubulin polymerization inhibition, strong and selective COX-2 inhibition, marked suppression of PGE-2 production, induction of G2/M cell-cycle arrest, and pronounced apoptotic effects associated with Bax upregulation, Bcl-2 downregulation, and activation of caspase-3 and caspase-9. In addition, 9l significantly inhibited HCA-7 cell migration, further supporting its anticancer potential. These experimental findings were consistent with the docking results, which demonstrated favorable accommodation of 9l within both the colchicine-binding site of tubulin and the active site of COX-2. At the same time, ADMET prediction and human liver microsomal stability studies suggested an acceptable drug-like and metabolic profile. Overall, compound 9l represents a promising lead for further optimization as a multitarget anticancer agent.

## 4. Experimental

### 4.1 Chemistry

#### 4.1.1 General details

Refer to SI.

3-Chloroacetylacetone (2),^46^ 1-(2-mercapto-4-methylthiazol-5-yl)ethan-1-one (3) and chalcones (4a–e),^22^ amino pyrazoles (7a–c) and chloroacetamides (8a–c)^40^ were prepared according to reported methods.

#### 4.1.2 General procedures for the synthesis of compounds 9a–o

To a well-stirred solution of the appropriate chalcones (4a–e) (1 mmol) and triethylamine (1.5 mmol, 0.152 mg) in acetonitrile, the pyrazole derivative (8a–c) (1 mmol) was added. The resulting clear mixture was stirred at room temperature for 6 hours. The formed precipitate was filtered off, washed with cold acetonitrile and distilled water, and dried. The crude product was recrystallized from acetonitrile/water.

#### 4.1.3 2-((5-Cinnamoyl-4-methylthiazol-2-yl)thio)-*N*-(1,3-diphenyl-1*H*-pyrazol-5-yl)acetamide (9a)

Yellow powder (396 mg, 74% yield), m.p. 219–221 °C; ^1^H NMR (500 MHz, DMSO-*d*_6_) *δ* (ppm): 10.51 (s, 1H), 7.85 (d, *J* = 8.5 Hz, 2H), 7.79–7.74 (m, 2H), 7.67 (d, *J* = 15.5 Hz, 1H), 7.56 (d, *J* = 7.6 Hz, 2H), 7.49–7.41 (m, 7H), 7.40–7.31 (m, 3H), 6.94 (s, 1H), 4.26 (s, 2H), 2.63 (s, 3H); ^13^C NMR (120 MHz, DMSO-*d*_6_) *δ* (ppm): 181.97, 168.77, 166.43, 158.48, 149.66, 144.23, 138.77, 137.48, 134.66, 133.14, 132.57, 132.10, 131.43, 129.75, 129.55, 129.31, 128.39, 128.32, 127.42, 124.86, 124.41, 99.91, 37.61, 18.79. Anal. Calcd. For C_32_H_28_N_4_O_4_S_2_: C, 64.41; H, 4.73; N, 9.39. Found: C, 64.58; H, 4.59; N, 9.58. MS (ESI) calcd for C_32_H_28_N_4_O_4_S_2_: 536.13, found [M]^+^: 536.38.

#### 4.1.4 (*E*)-2-((5-(3-(4-chlorophenyl)acryloyl)-4-methylthiazol-2-yl)thio)-*N*-(1,3-diphenyl-1*H*-pyrazol-5-yl)acetamide (9b)

Yellow powder (445 mg, 78% yield), m.p. 227–230 °C; ^1^H NMR (500 MHz, DMSO-*d*_6_) *δ* (ppm): 10.56 (s, 1H), 7.85 (d, *J* = 7.1 Hz, 2H), 7.81 (d, *J* = 6.9 Hz, 2H), 7.65 (d, *J* = 15.7 Hz, 1H), 7.56 (d, *J* = 6.9 Hz, 2H), 7.52–7.42 (m, 6H), 7.40–7.31 (m, 3H), 6.93 (s, 1H), 4.26 (s, 2H), 2.62 (s, 3H); ^13^C NMR (120 MHz, DMSO-*d*_6_) *δ* (ppm): 181.85, 168.91, 166.45, 158.62, 149.65, 142.75, 138.77, 137.47, 135.92, 133.61, 133.13, 132.49, 132.09, 131.00, 129.74, 129.57, 129.29, 128.30, 127.42, 125.55, 124.38, 99.95, 37.61, 18.79. Anal. Calcd. for C_30_H_23_ClN_4_O_2_S_2_: C, 63.09; H, 4.06; N, 9.81. Found: C, 62.95; H, 4.17; N, 9.63. MS (ESI) calcd for C_30_H_23_ClN_4_O_2_S_2_: 570.10, found [M]^+^: 571.07.

#### 4.1.5 (*E*)-2-((5-(3-(4-bromophenyl)acryloyl)-4-methylthiazol-2-yl)thio)-*N*-(1,3-diphenyl-1*H*-pyrazol-5-yl)acetamide (9c)

Yellow powder (473 mg, 77% yield), m.p. 226–228 °C; ^1^H NMR (500 MHz, DMSO-*d*_6_) *δ* (ppm): 10.54 (s, 1H, NH), 7.86 (d, *J* = 8.4 Hz, 2H), 7.74 (d, *J* = 7.9 Hz, 2H), 7.67–7.60 (m, 4H), 7.56 (d, *J* = 7.7 Hz, 2H), 7.49–7.43 (m, 4H), 7.40–7.34 (m, 2H), 6.94 (s, 1H), 4.26 (s, 2H), 2.63 (s, 3H); ^13^C NMR (120 MHz, DMSO-*d*_6_) *δ* (ppm): 181.90, 168.97, 166.40, 158.63, 149.62, 142.87, 138.77, 137.47, 133.98, 133.11, 132.52, 132.10, 131.25, 129.76, 129.30, 128.31, 127.43, 125.77, 125.62, 124.83, 124.40, 99.94, 37.60, 18.84. Anal. Calcd. for C_30_H_23_BrN_4_O_2_S_2_: C, 58.54; H, 3.77; N, 9.10. Found: C, 58.72; H, 3.65; N, 9.01. MS (ESI) calcd for C_30_H_23_BrN_4_O_2_S_2_: 614.04, found [M]^+^: 614.30.

#### 4.1.6 (*E*)-*N*-(1,3-diphenyl-1*H*-pyrazol-5-yl)-2-((4-methyl-5-(3-(*p*-tolyl)acryloyl)thiazol-2-yl)thio)acetamide (9d)

Yellow powder (462 mg, 84% yield), m.p. 241–243 °C; ^1^H NMR (500 MHz, DMSO-*d*_6_) *δ* (ppm): 10.50 (s, 1H), 7.85 (d, *J* = 8.3 Hz, 2H), 7.66 (d, *J* = 7.5 Hz, 3H), 7.64–7.53 (m, 3H), 7.51–7.43 (m, 4H), 7.40–7.34 (m, 1H), 7.31–7.21 (m, 3H), 6.94 (s, 1H), 4.26 (s, 2H), 2.62 (s, 3H), 2.31 (s, 3H); ^13^C NMR (120 MHz, DMSO-*d*_6_) *δ* (ppm): 181.86, 168.52, 166.40, 158.30, 149.66, 144.32, 141.59, 138.81, 137.50, 133.13, 132.62, 132.12, 131.94, 130.16, 129.75, 129.35, 129.27, 128.29, 127.42, 124.42, 123.76, 99.85, 37.68, 21.65, 18.76. Anal. Calcd. for C_31_H_26_N_4_O_2_S_2_: C, 67.61; H, 4.76; N, 10.17. Found: C, 67.45; H, 4.89; N, 10.37. MS (ESI) calcd for C_31_H_26_N_4_O_2_S_2_: 550.15, found [M]^+^: 550.69.

#### 4.1.7 (*E*)-*N*-(1,3-diphenyl-1*H*-pyrazol-5-yl)-2-((5-(3-(4-methoxyphenyl)acryloyl)-4-methylthiazol-2-yl)thio)acetamide (9e)

Yellow powder (475 mg, 84% yield), m.p. 236–238 °C; ^1^H NMR (500 MHz, DMSO-*d*_6_) *δ* (ppm): 10.50 (s, 1H), 7.85 (d, *J* = 5.9 Hz, 2H), 7.74 (d, *J* = 5.5 Hz, 2H), 7.64 (d, *J* = 16.0 Hz, 1H), 7.55 (d, *J* = 6.2 Hz, 2H), 7.49–7.34 (m, 6H), 7.19 (d, *J* = 14.4 Hz, 1H), 7.01–6.91 (m, 3H), 4.25 (s, 2H), 3.79 (s, 3H), 2.62 (s, 3H); ^13^C NMR (120 MHz, DMSO-*d*_6_) *δ* (ppm): 181.77, 168.21, 166.42, 162.14, 158.03, 149.66, 144.30, 138.82, 137.51, 133.14, 132.74, 132.12, 131.28, 129.74, 129.26, 128.28, 127.41, 127.27, 124.41, 122.26, 115.03, 99.82, 55.89, 37.67, 18.73. Anal. Calcd. for C_31_H_26_N_4_O_3_S_2_: C, 65.70; H, 4.62; N, 9.89. Found: C, 65.81; H, 4.45; N, 10.03. MS (ESI) calcd for C_31_H_26_N_4_O_3_S_2_: 566.14, found [M]^+^: 566.56.

#### 4.1.8 *N*-(3-(4-chlorophenyl)-1-phenyl-1*H*-pyrazol-5-yl)-2-((5-cinnamoyl-4-methylthiazol-2-yl)thio)acetamide (9f)

Yellow powder (450 mg, 79% yield), m.p. 231–233 °C; ^1^H NMR (500 MHz, DMSO-*d*_6_) *δ* (ppm): 10.50 (s, 1H), 7.85 (d, *J* = 8.5 Hz, 2H), 7.80–7.75 (m, 2H), 7.67 (d, *J* = 15.6 Hz, 1H), 7.56 (d, *J* = 7.6 Hz, 2H), 7.48–7.41 (m, 6H), 7.39–7.31 (m, 3H), 6.94 (s, 1H), 4.26 (s, 2H), 2.63 (s, 3H); ^13^C NMR (120 MHz, DMSO-*d*_6_) *δ* (ppm): 181.82, 168.76, 166.38, 158.45, 149.66, 144.26, 138.81, 137.82, 134.69, 133.23, 133.12, 132.11, 131.47, 129.74, 129.55, 129.32, 128.30, 127.71, 127.44, 124.94, 124.43, 99.91, 37.64, 18.76. Anal. Calcd. for C_30_H_23_ClN_4_O_2_S_2_: C, 63.09; H, 4.06; N, 9.81. Found: C, 62.88; H, 4.15; N, 9.68. MS (ESI) calcd for C_30_H_23_ClN_4_O_2_S_2_: 572.10, found [M+2H]^+^: 572.29.

#### 4.1.9 (*E*)-*N*-(3-(4-chlorophenyl)-1-phenyl-1*H*-pyrazol-5-yl)-2-((5-(3-(4-chlorophenyl)acryloyl)-4-methylthiazol-2-yl)thio)acetamide (9g)

Brown powder (466 mg, 77% yield), m.p. 205–207 °C; ^1^H NMR (500 MHz, DMSO-*d*_6_) *δ* (ppm): 10.56 (s, 1H), 7.85 (d, *J* = 8.4 Hz, 2H), 7.81 (d, *J* = 8.3 Hz, 2H), 7.65 (d, *J* = 15.5 Hz, 1H), 7.56 (d, *J* = 7.7 Hz, 2H), 7.50–7.43 (m, 6H), 7.38–7.33 (m, 2H), 6.93 (s, 1H), 4.26 (s, 2H), 2.63 (s, 3H); ^13^C NMR (120 MHz, DMSO-*d*_6_) *δ* (ppm): 181.88, 168.91, 166.47, 158.64, 149.62, 142.75, 138.78, 137.72, 137.51, 135.92, 131.08, 130.10, 129.74, 129.55, 129.27, 128.31, 128.27, 127.45, 125.60, 124.59, 124.39, 100.03, 37.67, 18.87. Anal. Calcd. for C_30_H_22_Cl_2_N_4_O_2_S_2_: C, 59.50; H, 3.66; N, 9.25. Found: C, 59.65; H, 3.56; N, 9.47. MS (ESI) calcd for C_30_H_22_Cl_2_N_4_O_2_S_2_: 604.06, found [M]^+^: 604.44.

#### 4.1.10 (*E*)-2-((5-(3-(4-bromophenyl)acryloyl)-4-methylthiazol-2-yl)thio)-*N*-(3-(4-chlorophenyl)-1-phenyl-1*H*-pyrazol-5-yl)acetamide (9h)

Brown powder (500 mg, 77% yield), m.p. 216–219 °C; ^1^H NMR (500 MHz, DMSO-*d*_6_) *δ* (ppm): 10.50 (s, 1H), 7.86 (d, *J* = 6.9 Hz, 2H), 7.74 (d, *J* = 6.9 Hz, 2H), 7.68–7.59 (m, 3H), 7.56 (d, *J* = 7.0 Hz, 2H), 7.50–7.43 (m, 4H), 7.36 (d, *J* = 11.8 Hz, 2H), 6.94 (s, 1H), 4.26 (s, 2H), 2.63 (s, 3H); ^13^C NMR (120 MHz, DMSO-*d*_6_) *δ* (ppm): 181.83, 168.92, 166.42, 158.65, 149.65, 142.84, 138.76, 137.47, 133.93, 133.13, 132.50, 132.07, 131.19, 129.75, 129.29, 128.31, 127.41, 125.57, 124.82, 124.39, 124.11, 99.87, 37.61, 18.77. Anal. Calcd. for C_30_H_22_BrClN_4_O_2_S_2_: C, 55.43; H, 3.41; N, 8.62. Found: C, 55.31; H, 3.60; N, 8.51. MS (ESI) calcd for C_30_H_22_BrClN_4_O_2_S_2_: 651.01, found [M+3H]^+^: 651.58.

#### 4.1.11 (*E*)-*N*-(3-(4-chlorophenyl)-1-phenyl-1*H*-pyrazol-5-yl)-2-((4-methyl-5-(3-(*p*-tolyl)acryloyl)thiazol-2-yl)thio)acetamide (9i)

Brown powder (520 mg, 89% yield), m.p. 196–199 °C; ^1^H NMR (500 MHz, DMSO-*d*_6_) *δ* (ppm): 10.50 (s, 1H), 7.85 (d, *J* = 8.5 Hz, 2H), 7.65 (t, *J* = 11.9 Hz, 3H), 7.55 (d, *J* = 7.5 Hz, 2H), 7.48–7.42 (m, 4H), 7.39–7.34 (m, 1H), 7.28 (d, *J* = 15.5 Hz, 1H), 7.24 (d, *J* = 7.8 Hz, 2H), 6.94 (s, 1H), 4.26 (s, 2H), 2.63 (s, 3H), 2.32 (s, 3H); ^13^C NMR (120 MHz, DMSO-*d*_6_) *δ* (ppm): 181.95, 168.52, 166.41, 158.28, 149.65, 144.36, 141.62, 138.80, 137.49, 133.14, 132.64, 132.13, 131.97, 130.19, 129.76, 129.38, 129.30, 128.31, 127.44, 124.41, 123.86, 99.99, 37.69, 21.65, 18.78. Anal. Calcd. for C_31_H_25_ClN_4_O_2_S_2_: C, 63.63; H, 4.31; N, 9.58. Found: C, 63.83; H, 4.18; N, 9.67. MS (ESI) calcd for C_31_H_25_ClN_4_O_2_S_2_: 584.11, found [M]^+^: 584.47.

#### 4.1.12 (*E*)-*N*-(3-(4-chlorophenyl)-1-phenyl-1*H*-pyrazol-5-yl)-2-((5-(3-(4-methoxyphenyl)acryloyl)-4-methylthiazol-2-yl)thio)acetamide (9j)

Yellow powder (444 mg, 74% yield), m.p. 216–219 °C; ^1^H NMR (500 MHz, DMSO-*d*_6_) *δ* (ppm): 10.50 (s, 1H), 7.85 (d, *J* = 8.5 Hz, 2H), 7.74 (d, *J* = 8.6 Hz, 2H), 7.64 (d, *J* = 15.4 Hz, 1H), 7.55 (d, *J* = 7.6 Hz, 2H), 7.50–7.41 (m, 4H), 7.39–7.34 (m, 1H), 7.19 (d, *J* = 15.4 Hz, 1H), 7.00–6.92 (m, 3H), 4.25 (s, 2H), 3.79 (s, 3H), 2.62 (s, 3H); ^13^C NMR (120 MHz, DMSO-*d*_6_) *δ* (ppm): 181.83, 168.26, 166.44, 162.15, 158.02, 149.64, 144.33, 138.79, 137.49, 133.12, 132.75, 132.11, 131.32, 129.76, 129.29, 128.30, 127.42, 127.27, 124.40, 122.30, 115.06, 99.92, 55.96, 37.63, 18.75. Anal. Calcd. for C_31_H_25_ClN_4_O_3_S_2_: C, 61.94; H, 4.19; N, 9.32. Found: C, 61.75; H, 4.35; N, 9.10. MS (ESI) calcd for C_31_H_25_ClN_4_O_3_S_2_: 601.11, found [M + H]^+^: 601.50.

#### 4.1.13 2-((5-Cinnamoyl-4-methylthiazol-2-yl)thio)-*N*-(3-(4-methoxyphenyl)-1-phenyl-1*H*-pyrazol-5-yl)acetamide (9k)

Yellow powder (453 mg, 80% yield), m.p. 190–191 °C; ^1^H NMR (500 MHz, DMSO-*d*_6_) *δ* (ppm): 10.46 (s, 1H), 7.81–7.73 (m, 4H), 7.67 (d, *J* = 15.4 Hz, 1H), 7.55 (d, *J* = 8.0 Hz, 2H), 7.49–7.41 (m, 5H), 7.38–7.30 (m, 2H), 6.95 (d, *J* = 8.4 Hz, 2H), 6.82 (s, 1H), 4.26 (s, 2H), 3.75 (s, 3H), 2.64 (s, 3H); ^13^C NMR (120 MHz, DMSO-*d*_6_) *δ* (ppm): 181.90, 168.80, 166.38, 159.77, 158.51, 150.71, 144.21, 138.97, 137.13, 134.65, 132.55, 131.41, 129.70, 129.54, 129.30, 128.00, 127.06, 125.86, 124.81, 124.27, 114.63, 99.32, 55.61, 37.66, 18.78. Anal. Calcd. for C_31_H_26_N_4_O_3_S_2_: C, 65.70; H, 4.62; N, 9.89. Found: C, 65.80; H, 4.41; N, 10.05. MS (ESI) calcd for C_31_H_26_N_4_O_3_S_2_: 566.14, found [M]^+^: 566.29.

#### 4.1.14 (*E*)-2-((5-(3-(4-chlorophenyl)acryloyl)-4-methylthiazol-2-yl)thio)-*N*-(3-(4-methoxyphenyl)-1-phenyl-1*H*-pyrazol-5-yl)acetamide (9l)

Yellow powder (456 mg, 76% yield), m.p. 214–216 °C; ^1^H NMR (500 MHz, DMSO-*d*_6_) *δ* (ppm): 10.51 (s, 1H), 7.81 (d, *J* = 7.9 Hz, 2H), 7.75 (d, *J* = 7.8 Hz, 2H), 7.65 (d, *J* = 15.5 Hz, 1H), 7.55 (d, *J* = 7.6 Hz, 2H), 7.50–7.42 (m, 4H), 7.39–7.30 (m, 2H), 6.95 (d, *J* = 7.8 Hz, 2H), 6.82 (s, 1H), 4.26 (s, 2H), 3.75 (s, 3H), 2.63 (s, 3H); ^13^C NMR (120 MHz, DMSO-*d*_6_) *δ* (ppm): 181.81, 168.92, 166.41, 159.78, 158.64, 150.69, 142.72, 138.97, 137.12, 135.92, 133.61, 132.45, 130.98, 129.69, 129.56, 127.99, 127.05, 125.87, 125.54, 124.24, 114.64, 99.45, 55.67, 37.67, 18.79. Anal. Calcd. for C_31_H_25_ClN_4_O_3_S_2_: C, 61.94; H, 4.19; N, 9.32. Found: C, 61.81; H, 4.31; N, 9.15. MS (ESI) calcd for C_31_H_25_ClN_4_O_3_S_2_: 600.11, found [M]^+^: 600.81.

#### 4.1.15 (*E*)-2-((5-(3-(4-bromophenyl)acryloyl)-4-methylthiazol-2-yl)thio)-*N*-(3-(4-methoxyphenyl)-1-phenyl-1*H*-pyrazol-5-yl)acetamide (9m)

Yellow powder (477 mg, 74% yield), m.p. 206–208 °C; ^1^H NMR (500 MHz, DMSO-*d*_6_) *δ* (ppm): 10.46 (s, 1H), 7.77–7.71 (m, 4H), 7.66–7.60 (m, 3H), 7.55 (d, *J* = 7.6 Hz, 2H), 7.49–7.42 (m, 2H), 7.39–7.32 (m, 2H), 6.95 (d, *J* = 8.5 Hz, 2H), 6.82 (s, 1H), 4.26 (s, 2H), 3.76 (s, 3H), 2.63 (s, 3H); ^13^C NMR (120 MHz, DMSO-*d*_6_) *δ* (ppm): 181.71, 168.90, 166.36, 159.77, 158.68, 150.71, 143.11, 142.79, 138.96, 137.13, 133.89, 132.47, 131.12, 129.70, 128.00, 127.05, 125.86, 125.49, 124.82, 124.26, 114.63, 99.31, 55.61, 37.70, 18.79. Anal. Calcd. for C_31_H_25_BrN_4_O_3_S_2_: C, 57.67; H, 3.90; N, 8.68. Found: C, 57.89; H, 3.81; N, 8.81. MS (ESI) calcd for C_31_H_25_BrN_4_O_3_S_2_: 644.06, found [M]^+^: 643.88.

#### 4.1.16 (*E*)-*N*-(3-(4-methoxyphenyl)-1-phenyl-1*H*-pyrazol-5-yl)-2-((4-methyl-5-(3-(*p*-tolyl)acryloyl)thiazol-2-yl)thio)acetamide (9n)

Brown powder (435 mg, 75% yield), m.p. 225–228 °C; ^1^H NMR (500 MHz, DMSO-*d*_6_) *δ* (ppm): 10.45 (s, 1H), 7.75 (d, *J* = 8.7 Hz, 2H), 7.65 (t, *J* = 11.9 Hz, 3H), 7.54 (d, *J* = 7.6 Hz, 2H), 7.45 (t, *J* = 7.7 Hz, 2H), 7.37–7.32 (m, 1H), 7.28 (d, *J* = 15.5 Hz, 1H), 7.24 (d, *J* = 7.8 Hz, 2H), 6.95 (d, *J* = 8.7 Hz, 2H), 6.82 (s, 1H), 4.25 (s, 2H), 3.76 (s, 3H), 2.63 (s, 3H), 2.32 (s, 3H); ^13^C NMR (120 MHz, DMSO-*d*_6_) *δ* (ppm): 181.92, 168.59, 166.41, 159.77, 158.34, 150.69, 144.36, 141.65, 138.94, 137.11, 132.61, 131.92, 130.19, 129.71, 129.37, 128.02, 127.06, 125.84, 124.25, 123.75, 114.64, 99.37, 55.62, 37.62, 21.64, 18.76. Anal. Calcd. for C_32_H_28_N_4_O_3_S_2_: C, 66.19; H, 4.86; N, 9.65. Found: C, 66.08; H, 5.09; N, 9.50. MS (ESI) calcd for C_32_H_28_N_4_O_3_S_2_: 580.16, found [M]^+^: 580.44.

#### 4.1.17 (*E*)-*N*-(3-(4-methoxyphenyl)-1-phenyl-1*H*-pyrazol-5-yl)-2-((5-(3-(4-methoxyphenyl)acryloyl)-4-methylthiazol-2-yl)thio)acetamide (9o)

Yellow powder (477 mg, 80% yield), m.p. 220–221 °C; ^1^H NMR (500 MHz, DMSO-*d*_6_) *δ* (ppm): 10.46 (s, 1H), 7.78–7.71 (m, 4H), 7.64 (d, *J* = 15.4 Hz, 1H), 7.55 (d, *J* = 7.8 Hz, 2H), 7.49–7.41 (m, 2H), 7.38–7.31 (m, 1H), 7.19 (d, *J* = 15.4 Hz, 1H), 6.97 (dd, *J* = 11.6, 8.7 Hz, 4H), 6.82 (s, 1H), 4.25 (s, 2H), 3.78 (s, 3H), 3.75 (s, 3H), 2.62 (s, 3H); ^13^C NMR (120 MHz, DMSO-*d*_6_) *δ* (ppm): 181.81, 168.25, 166.40, 162.15, 159.79, 158.04, 150.70, 144.30, 138.98, 137.14, 132.74, 131.29, 129.70, 128.00, 127.27, 127.06, 125.89, 124.26, 122.30, 115.05, 114.64, 99.36, 55.95, 55.67, 37.66, 18.72. Anal. Calcd. for C_32_H_28_N_4_O_4_S_2_: C, 64.41; H, 4.73; N, 9.39. Found: C, 64.58; H, 4.59; N, 9.58. MS (ESI) calcd for C_32_H_28_N_4_O_4_S_2_: 596.16, found [M]^+^: 596.99.

### 4.2 Biology

#### 4.2.1. Antiproliferative activity

The antiproliferative activities of compounds 9a–o were evaluated against MDA-MB-231, HCA-7, and A549 cancer cell lines, as well as MCF-10A normal mammary epithelial cells, using the MTT assay (Sigma-Aldrich *In Vitro* Toxicology Assay Kit, MTT-based, TOX-1), with combretastatin A-4 and doxorubicin as reference drugs. IC_50_ values were determined from the corresponding dose–response curves. Further experimental details are provided in the SI.

#### 4.2.2. Tubulin polymerization inhibitory assay

The effects of compounds 9a–o on tubulin polymerization were assessed using a fluorescence-based tubulin polymerization assay (Cytoskeleton, Inc., Cat. No. BK011P), with combretastatin A-4 as the reference inhibitor. The inhibitory activities were expressed as IC_50_ values. Additional assay details are provided in the SI.

#### 4.2.3. COX inhibition assay

The inhibitory activities of compounds 9h, 9j, 9l, 9m, and 9o against COX-1 and COX-2 were evaluated using the Cayman COX (ovine/human) Inhibitor Screening Assay Kit (Item No. 560131). The inhibitory activities were expressed as IC_50_ values, and the selectivity index (SI) was calculated as IC_50_(COX-1)/IC_50_(COX-2). Further methodological details are given in the SI.

#### 4.2.4. Cell cycle analysis and apoptosis

The effects of compound 9l on cell-cycle progression and apoptosis in HCA-7 cells were analyzed by flow cytometry using propidium iodide staining and Annexin V-FITC/PI dual staining, respectively. The detailed experimental procedures are described in the SI.

#### 4.2.5. Effect on Bax and Bcl-2 protein expression

The effect of compound 9l on Bax and Bcl-2 protein expression in HCA-7 cells was evaluated using ELISA kits (DRG Human BAX ELISA Kit, Cat. No. EIA-4487; Zymed Bcl-2 ELISA Kit, Cat. no. 99-0042). Full assay details are provided in the SI.

#### 4.2.6. Effect on Caspase-3 and Caspase-9 levels

The effects of compound 9l on caspase-3 and caspase-9 levels in HCA-7 cells were determined using ELISA-based assays (Invitrogen Human Active Caspase-3 ELISA Kit, Cat. No. KHO1091; Thermo Fisher Human Caspase-9 ELISA Kit, Cat. No. BMS2025). Additional methodological details are given in the SI.

#### 4.2.7. Effect on PGE-2 production

The effect of compound 9l on PGE-2 production in HCA-7 cells was assessed using a competitive ELISA assay (R&D Systems Parameter™ PGE-2 Immunoassay, Cat. No. KGE004B). Further experimental details are provided in the SI.

#### 4.2.8. Wound healing assay

The effect of compound 9l on the migratory behavior of HCA-7 cells was evaluated using the CytoSelect™ 24-Well Wound Healing Assay (Cell Biolabs, Inc., Cat. No. CBA-120). The detailed experimental procedure is described in the SI.

#### 4.2.9. Metabolic stability studies

The *in vitro* metabolic stability of compound 9l was evaluated using pooled human liver microsomes under NADPH-supported oxidative conditions. Detailed incubation conditions, sample processing, and data analysis are provided in the SI.

### 4.3 Computational studies

The computational studies of compound 9l comprised molecular docking and ADMET prediction. Docking against tubulin and COX-2 was performed using AutoDock Vina, with interaction analysis performed in Discovery Studio Visualizer, while ADMETlab 3.0 was used for *in silico* ADMET assessment. Full details are given in the SI.

## Author contributions

conceptualization, B. A. A. S., K. S. A. and S. B.; methodology, B. A. A. S., A. A. Q., A. R. S., G. A. and A. A.; validation, K. S. A. and S. B.; formal analysis, A. A. Q., A. R. S., M. A.-Z., G. A. and A. A.; investigation, B. A. A. S., A. R. S., M. A.-Z., G. A. and A. A.; resources, A. A. Q., M. A.-Z. and S. B.; data curation, B. A. A. S. and M. A.-Z.; writing—original draft, B. A. A. S. and A. R. S.; writing—review and editing, K. S. A. and S. B.; supervision, K. S. A. and S. B.; project administration, A. A. Q.; funding acquisition, A. A. Q. All authors have read and agreed to the published version of the manuscript.

## Conflicts of interest

The authors declare no conflicts of interest.

## Supplementary Material

RA-016-D6RA03557D-s001

## Data Availability

The original contributions presented in this study are included in the article; further inquiries can be directed to the corresponding author. Supplementary information (SI) is available. See DOI: https://doi.org/10.1039/d6ra03557d.
