# Supplementary material for: Design, synthesis, and mechanistic evaluation of novel pyrazole/thiazole chalcone hybrids as dual tubulin polymerization and COX-2 inhibitors with potent antiproliferative activity
Source: RSC Adv. 2026 Jun 2;16(33):30052–69. doi: 10.1039/d6ra03557d (PMC13231365; doi:10.1039/d6ra03557d)
Supplement: RA-016-D6RA03557D-s001 [file RA-016-D6RA03557D-s001.pdf]

Supplementary Information (SI) for RSC Advances.
This journal is © The Royal Society of Chemistry 2026

Design, Synthesis, and Mechanistic Evaluation of Novel Pyrazole/Thiazole Chalcone Hybrids as Dual Tubulin Polymerization and COX-2 Inhibitors with Potent Antiproliferative Activity

Basima A. A. Saleem[1], Ashraf A. Qurtam[2], Abdelrahman R Shalabi[3], Mohammed Al-zharani[2], Kasim Sakran Abass[4,*], Stefan Bräse[5,*], Ghallab Alotaibi[6], Abdullah Alkhammash[6]

[1]Department of Chemistry, College of Science, University of Mosul, Mosul 41001, Iraq

[2]Biology Department, College of Science, Imam Mohammad Ibn Saud Islamic University (IMSIU), Riyadh 11623, Saudi Arabia

[3]Pharmaceutical Chemistry Department, Faculty of Pharmacy, Sinai University, North Sinai, Egypt

[4]Department of Physiology, Biochemistry and Pharmacology, College of Veterinary Medicine, University of Kirkuk, Kirkuk 36001, Iraq

[5]Institute of Biological and Chemical Systems—Functional Molecular Systems (IBCS-FMS), Karlsruhe Institute of Technology (KIT), Kaiserstrasse 12, 76131 Karlsruhe, Germany

[6]Department of Pharmacology, College of Pharmacy, Al-Dawadmi Campus, Shaqra University, Shaqra 11961, Saudi Arabia

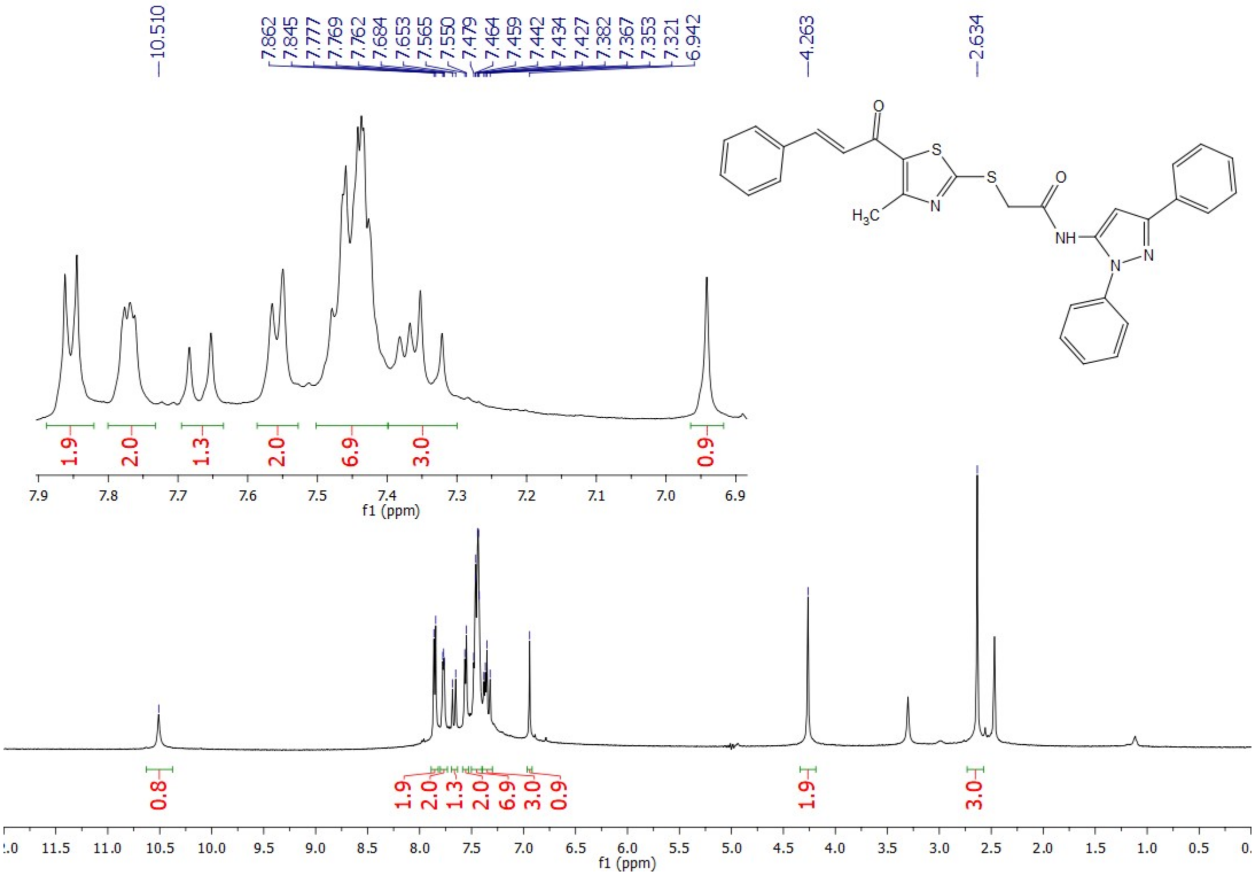

Figure S1. ^{1}H NMR spectrum of compound **9a**

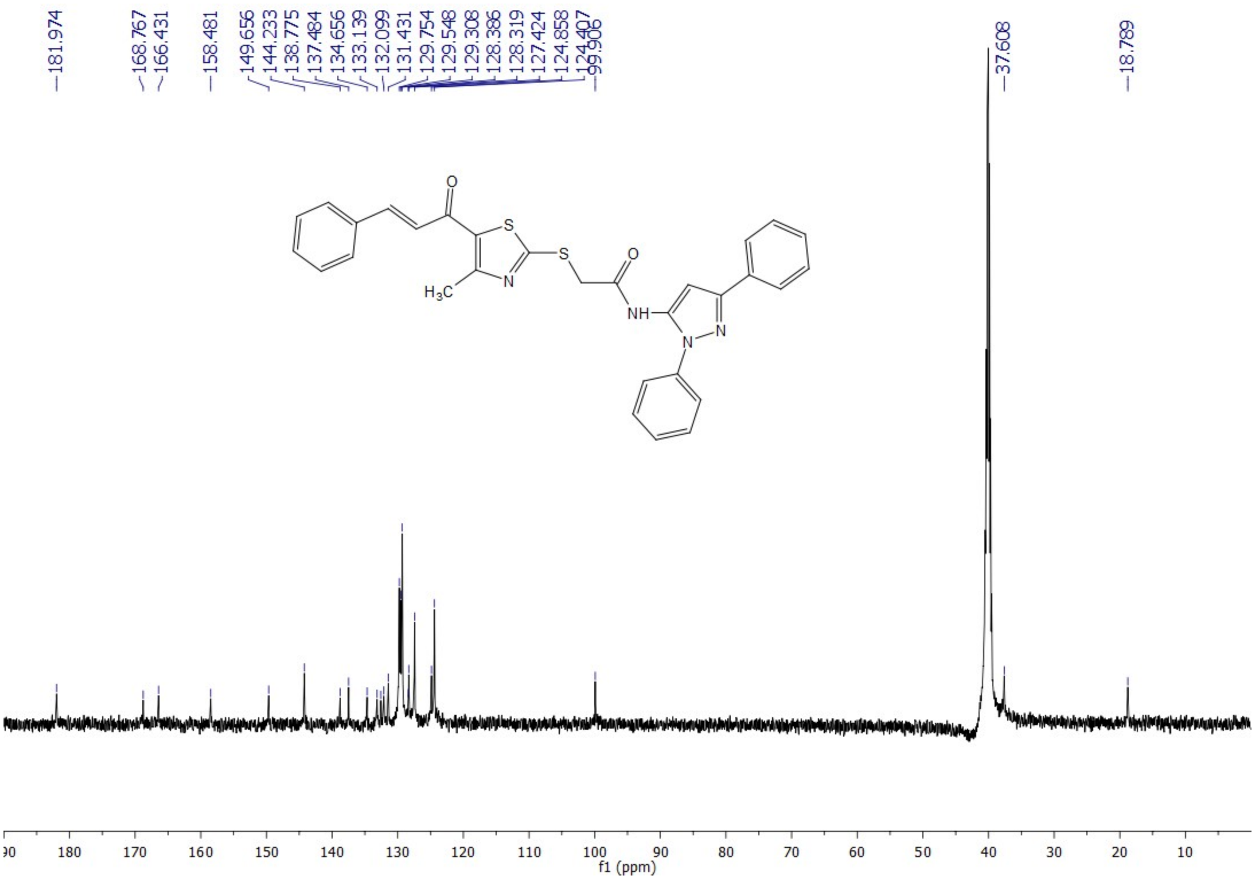

Figure S2. ^{13}C NMR spectrum of compound **9a**

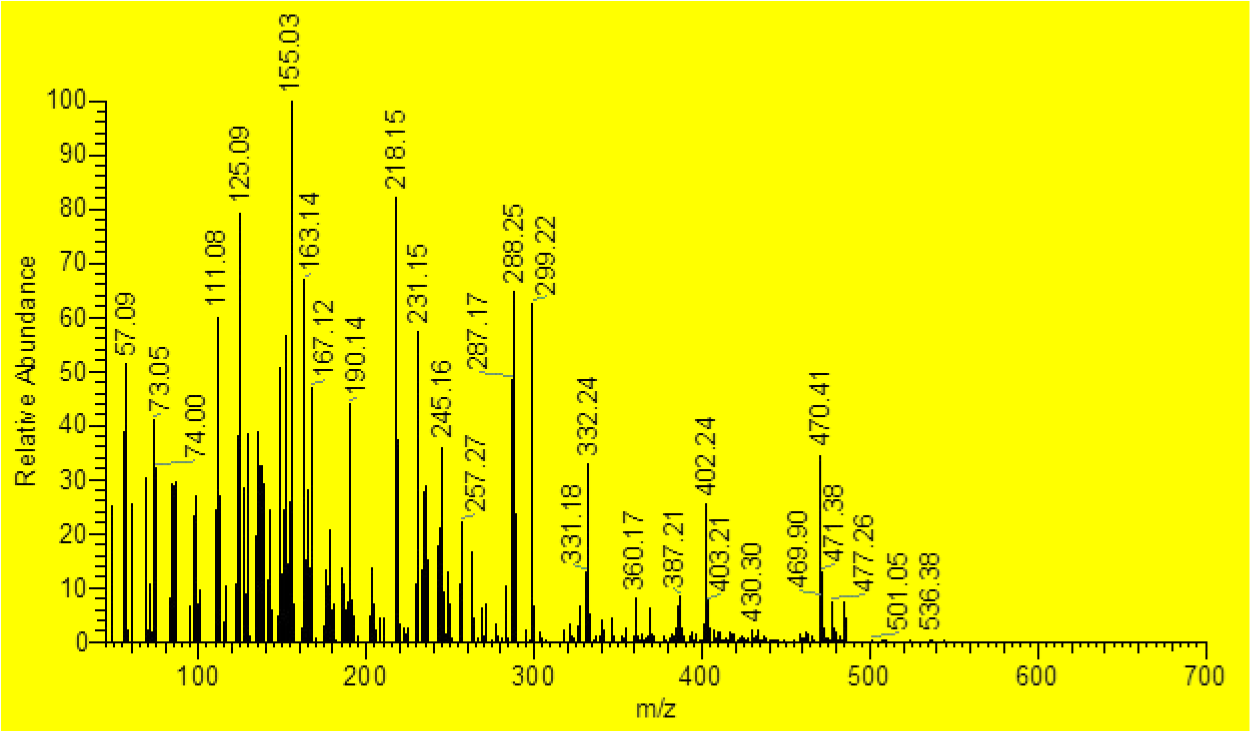

Figure S3. ESI-MS spectrum of compound **9a**

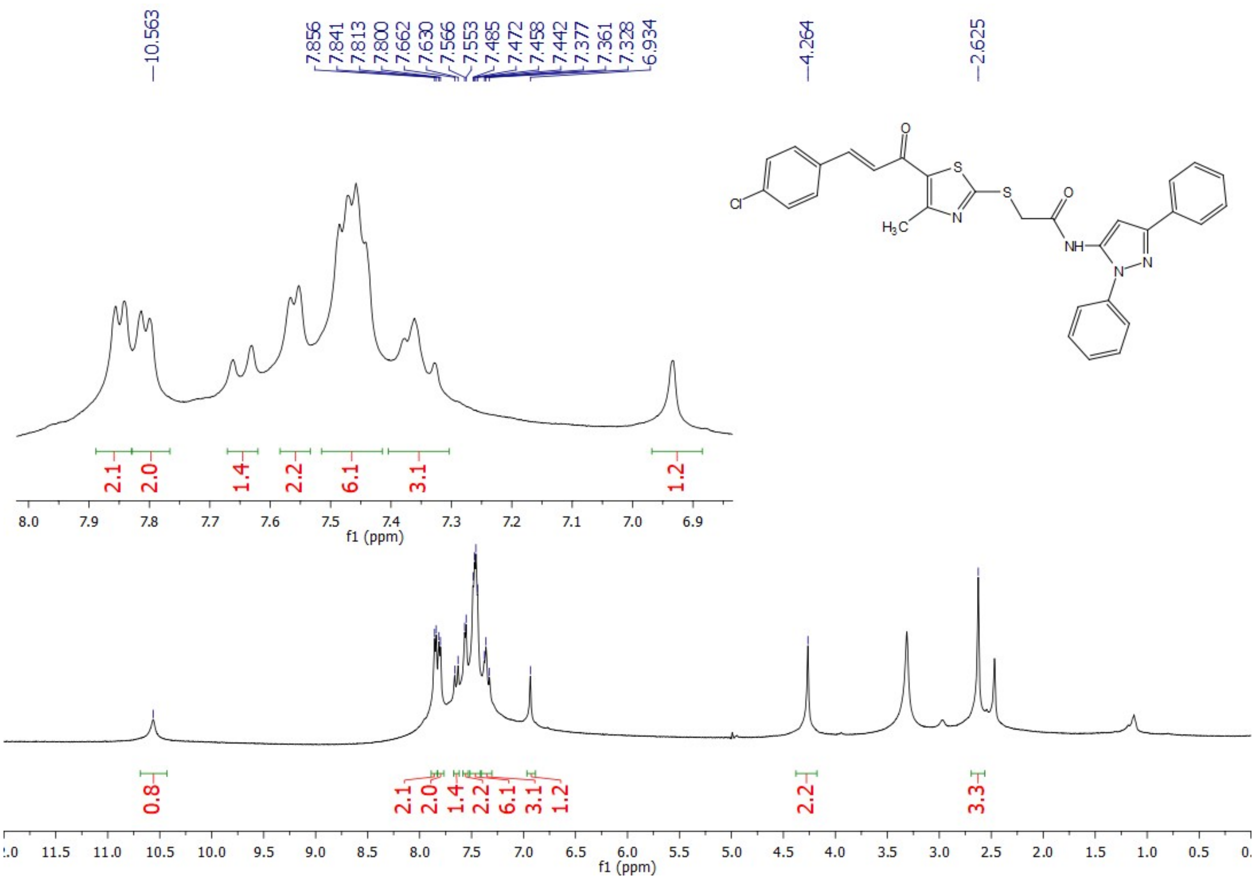

Figure S4. ^{1}H NMR spectrum of compound **9b**

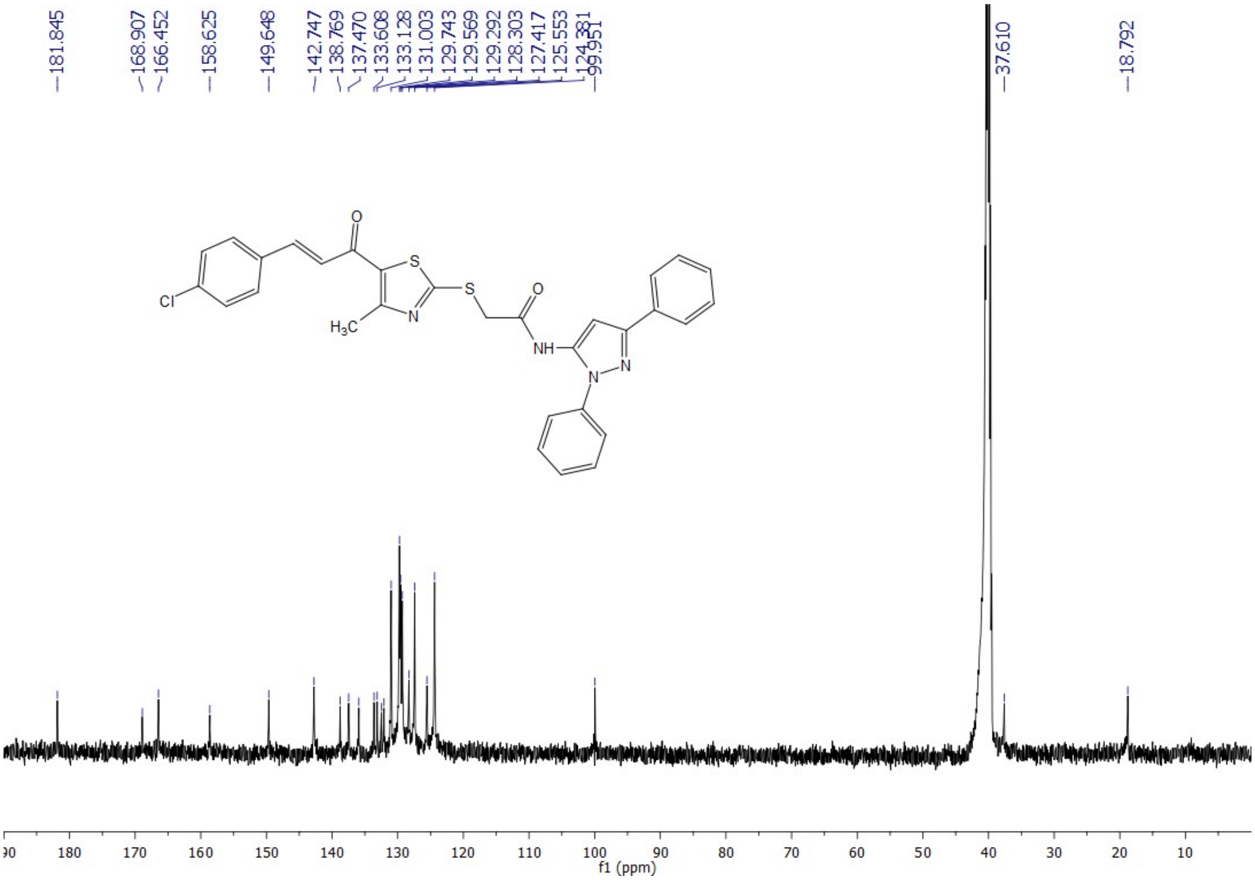

Figure S5. ^{13}C NMR spectrum of compound **9b**

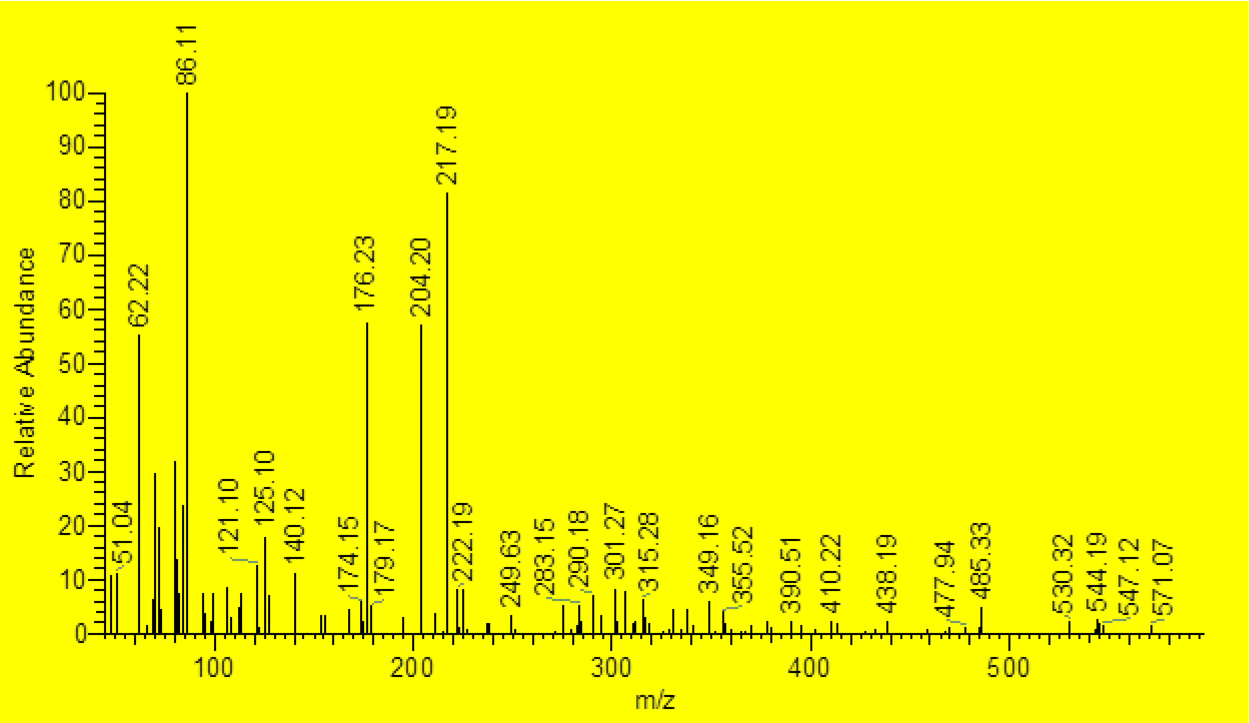

Figure S6. ESI-MS spectrum of compound **9b**

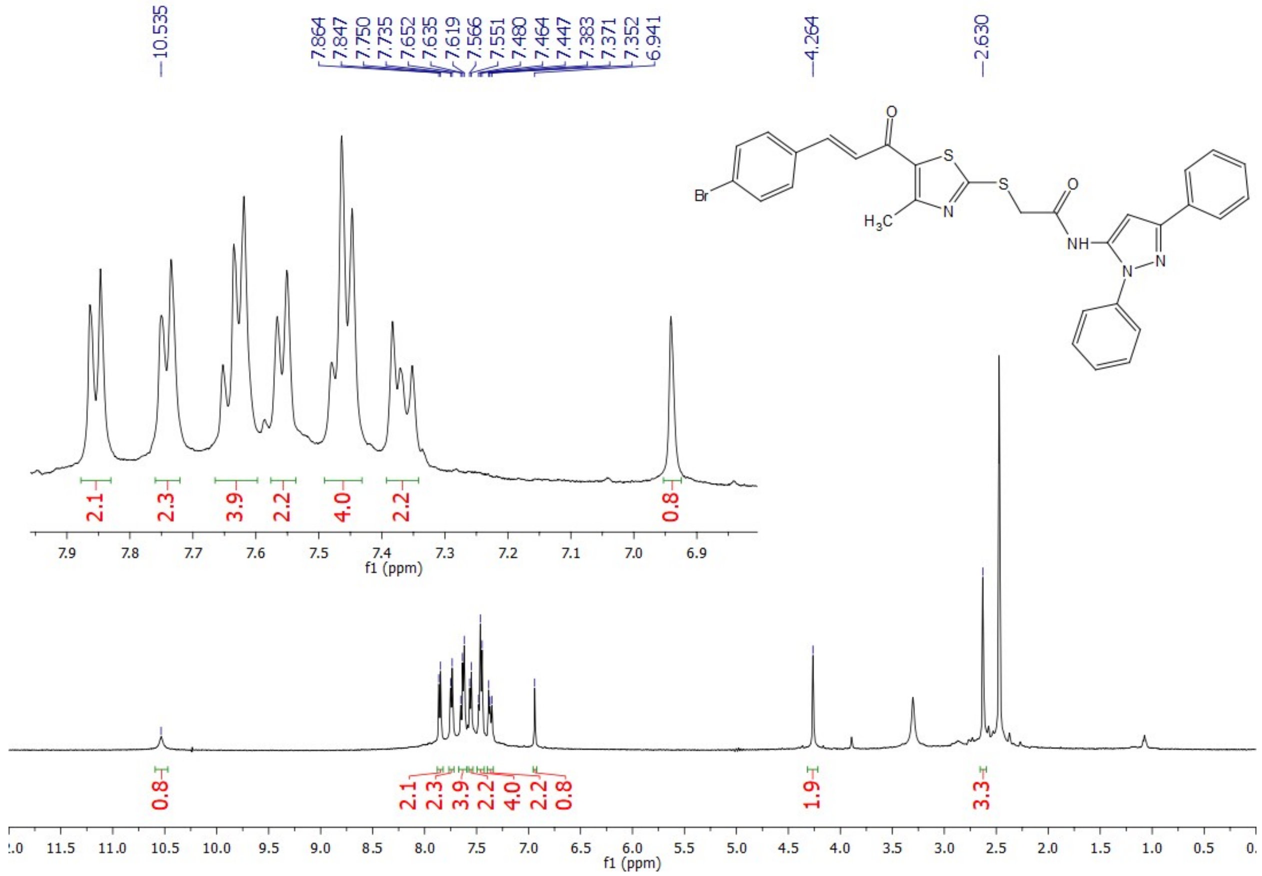

Figure S7. ^{1}H NMR spectrum of compound **9c**

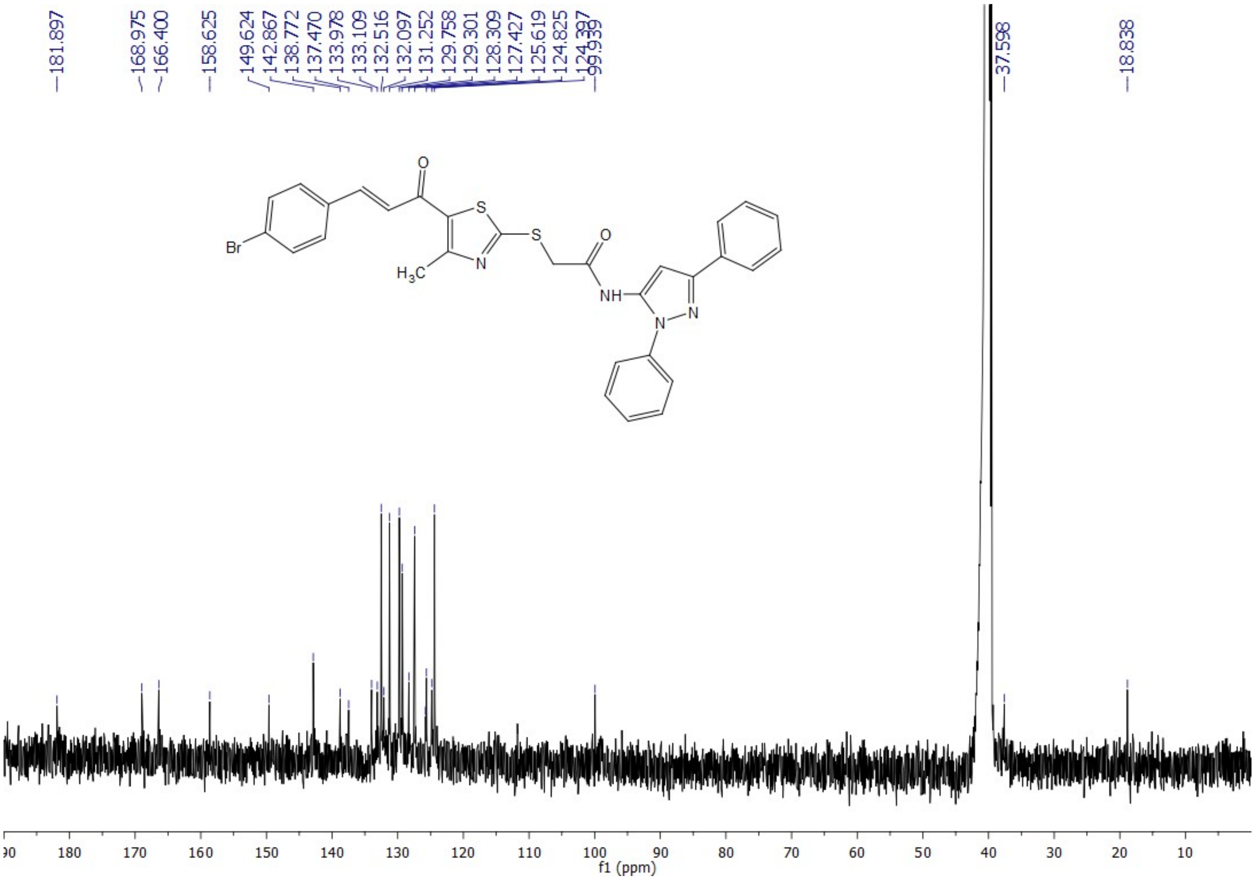

Figure S8. ^{13}C NMR spectrum of compound **9c**

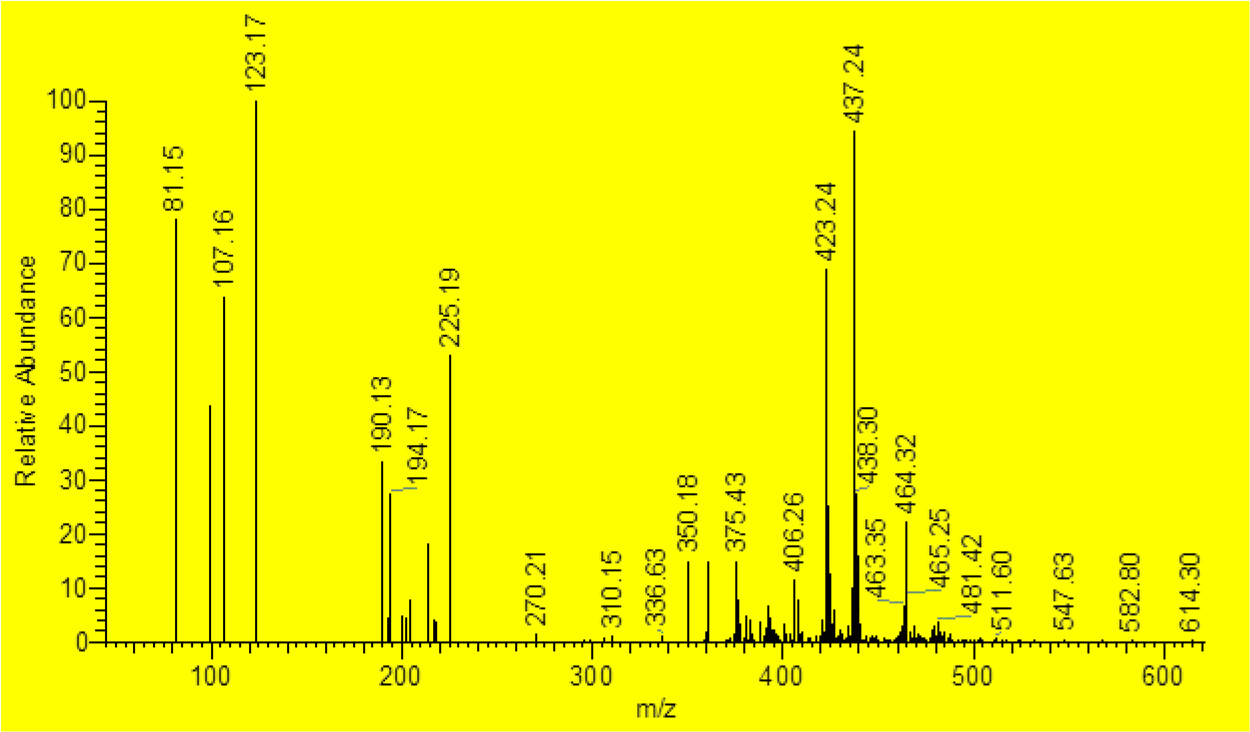

Figure S9. ESI-MS spectrum of compound **9c**

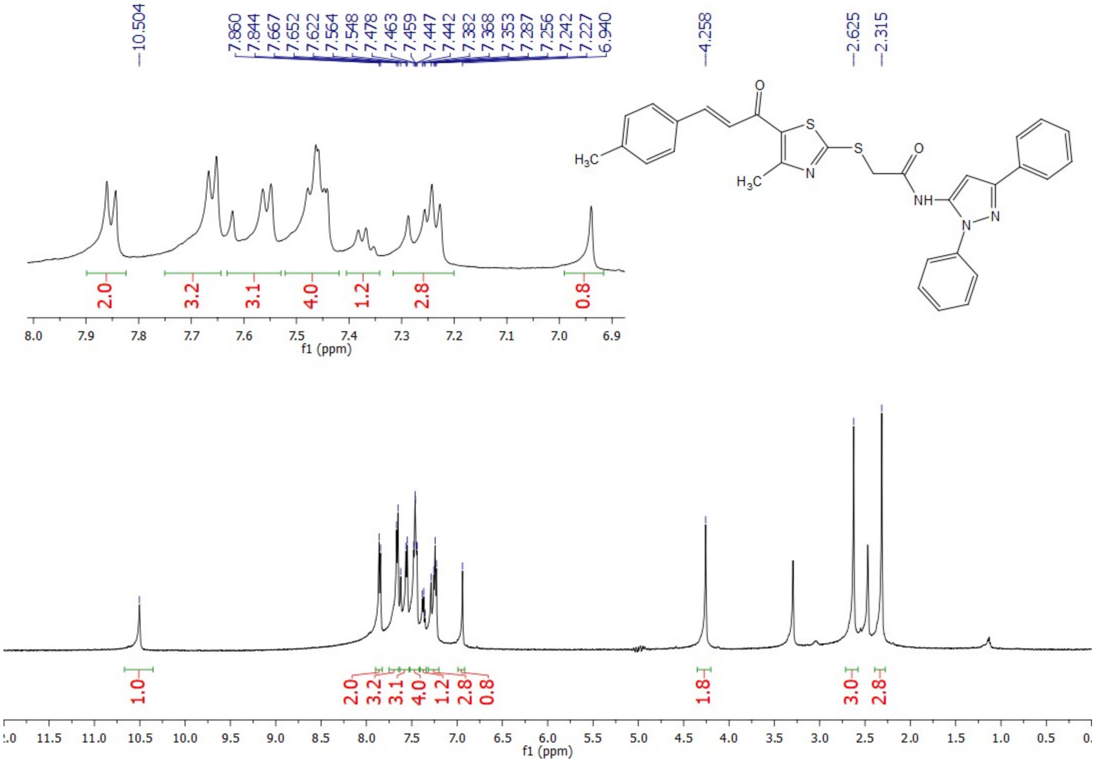

Figure S10. ^{1}H NMR spectrum of compound **9d**

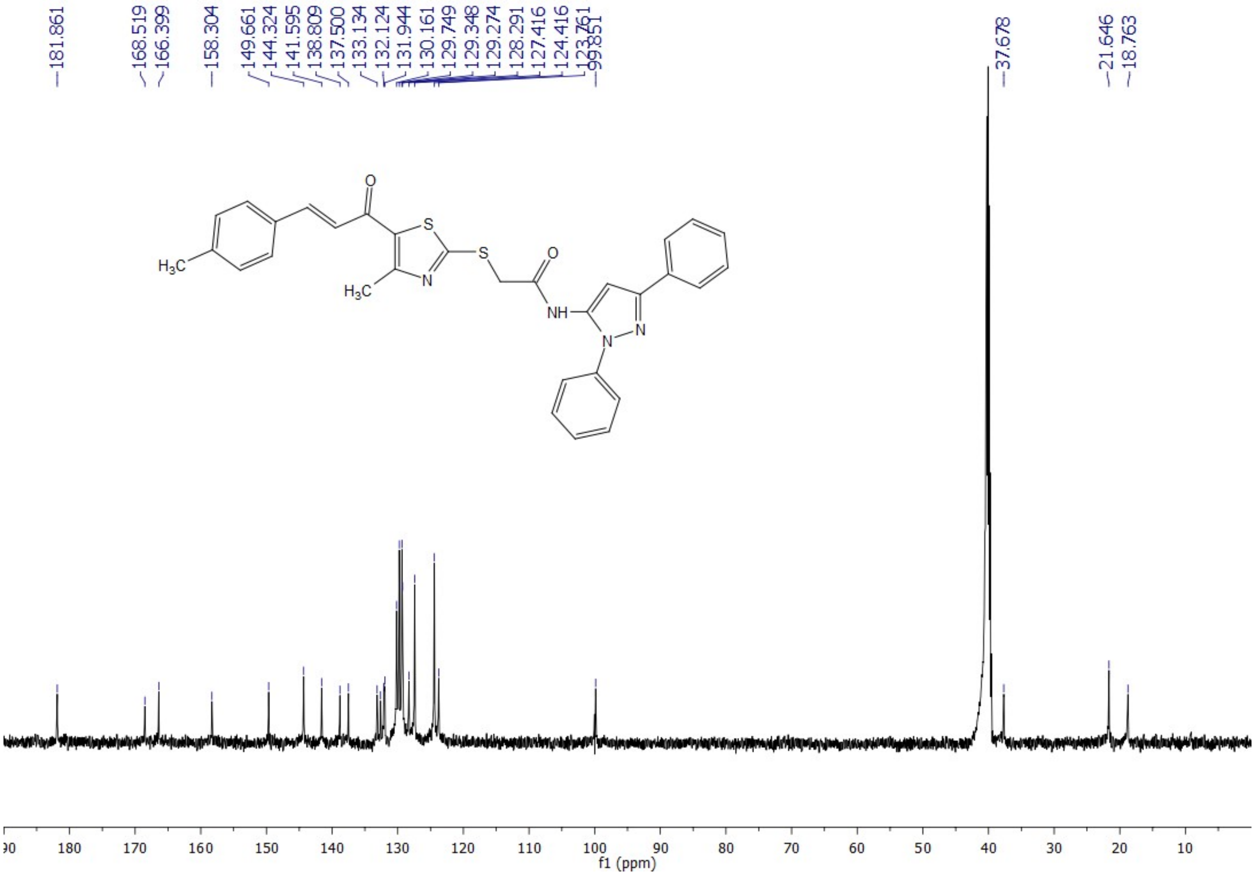

Figure S11. ^{13}C NMR spectrum of compound **9d**

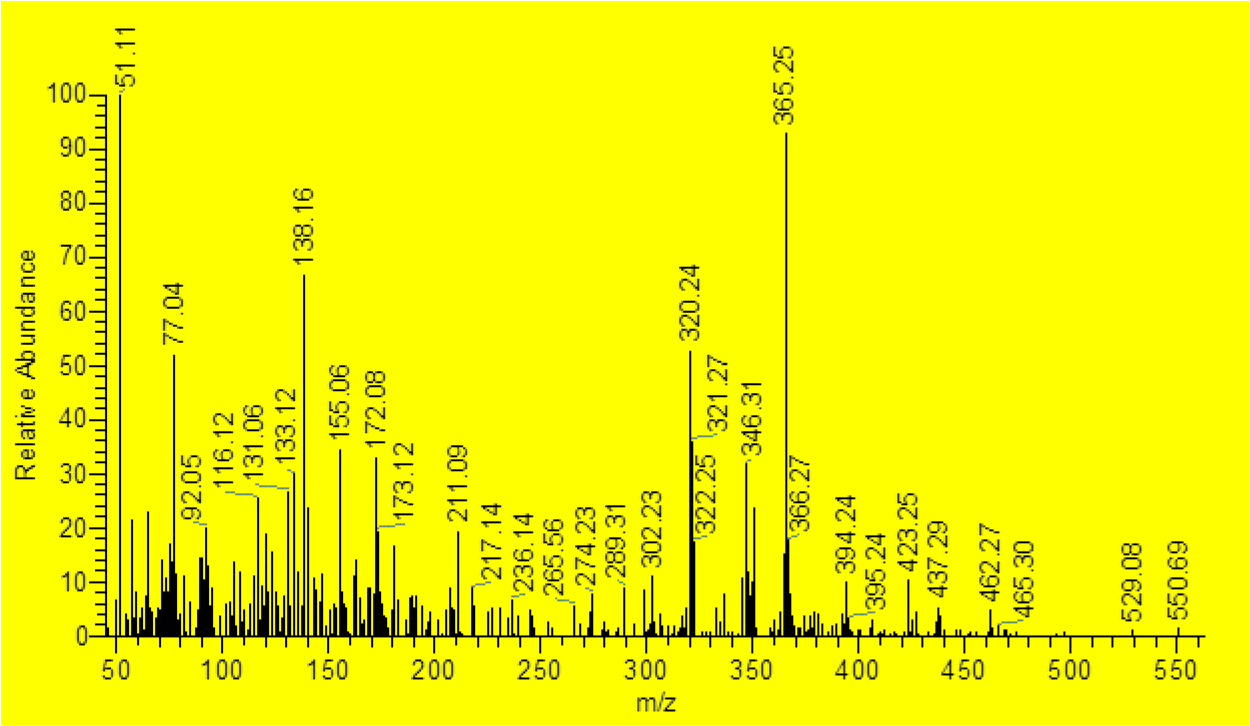

Figure S12. ESI-MS spectrum of compound **9d**

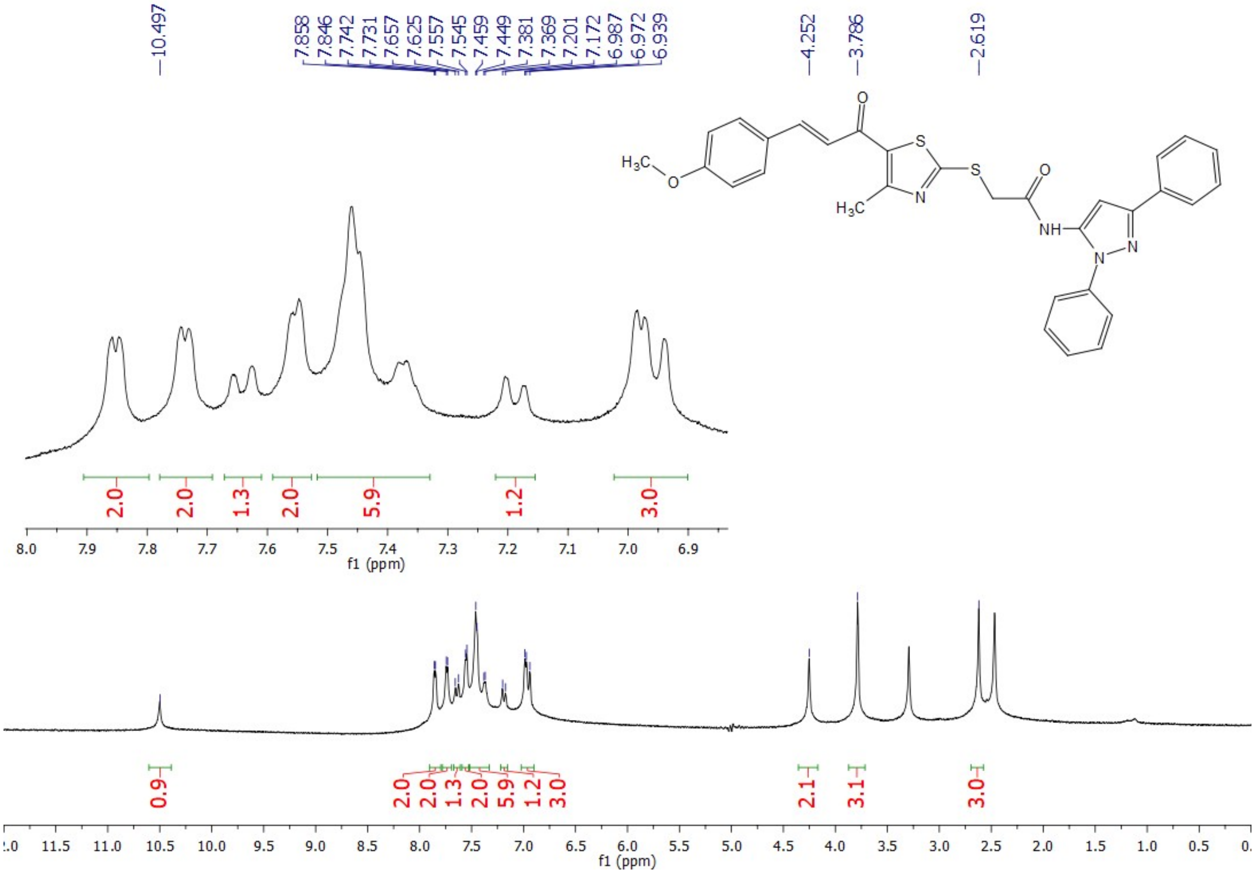

Figure S13. ^{1}H NMR spectrum of compound **9e**

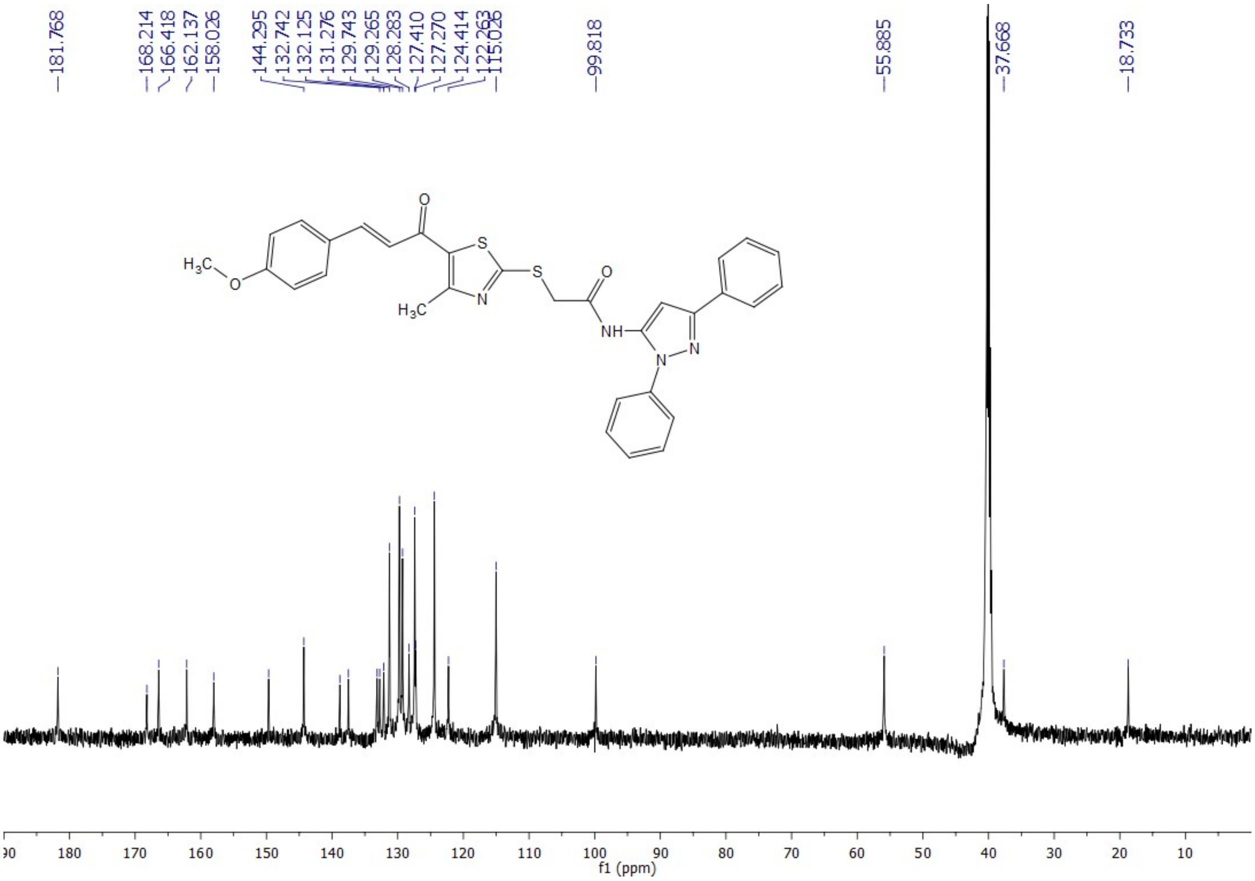

Figure S14. ^{13}C NMR spectrum of compound **9e**

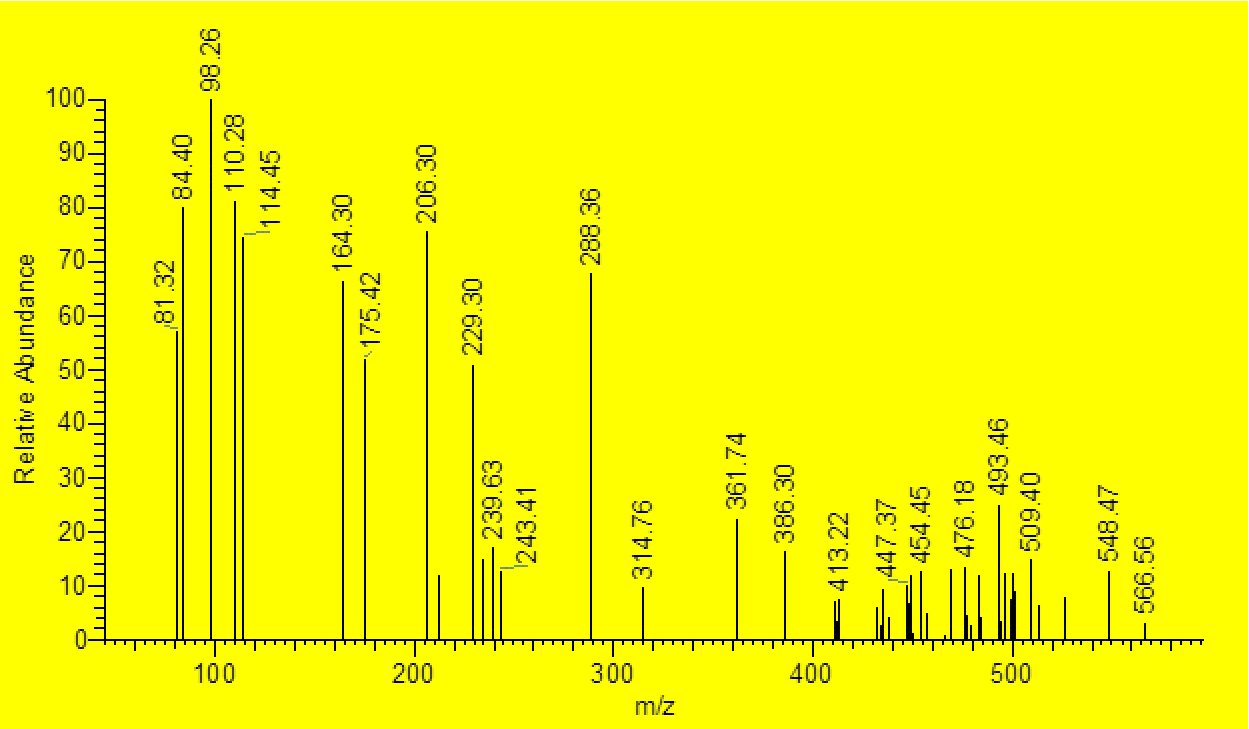

Figure S15. ESI-MS spectrum of compound **9e**

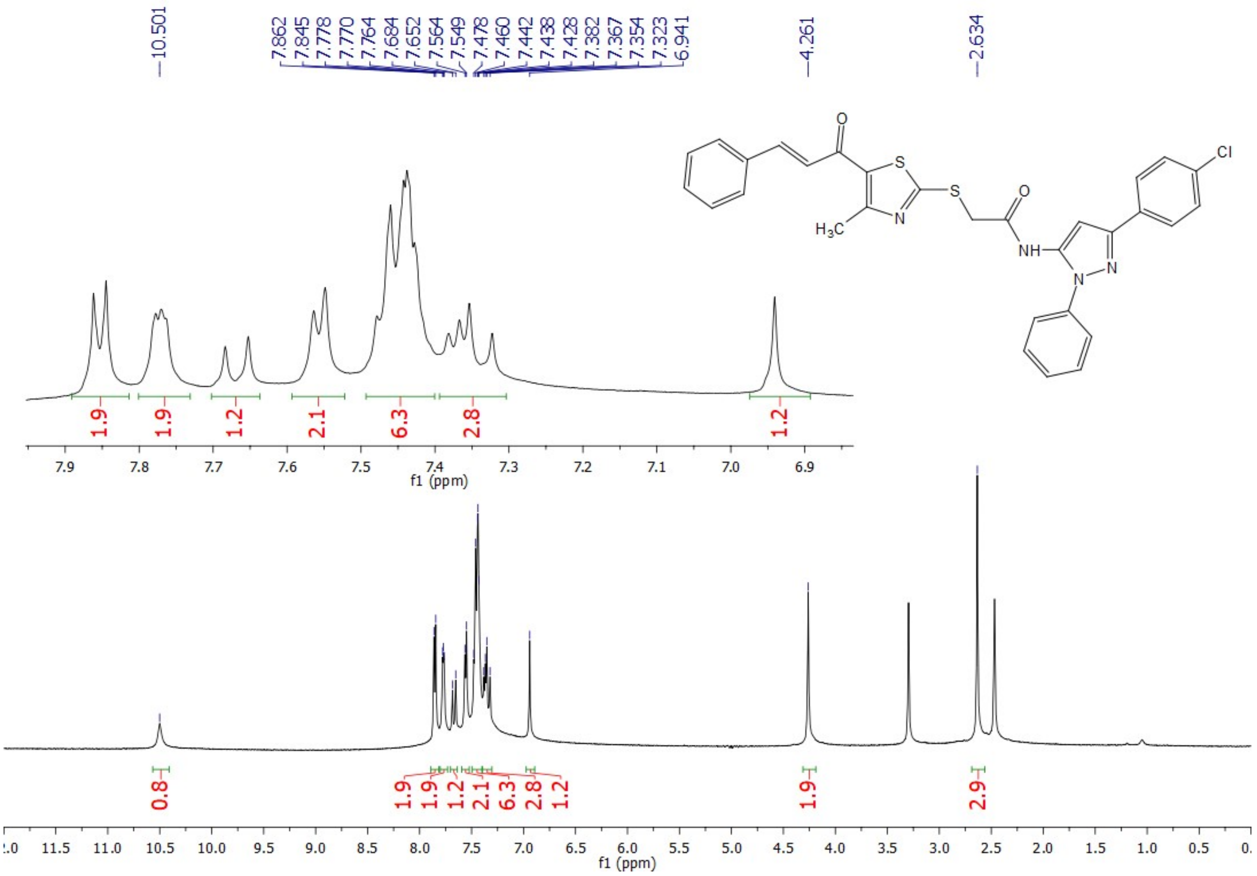

Figure S16. ^{1}H NMR spectrum of compound **9f**

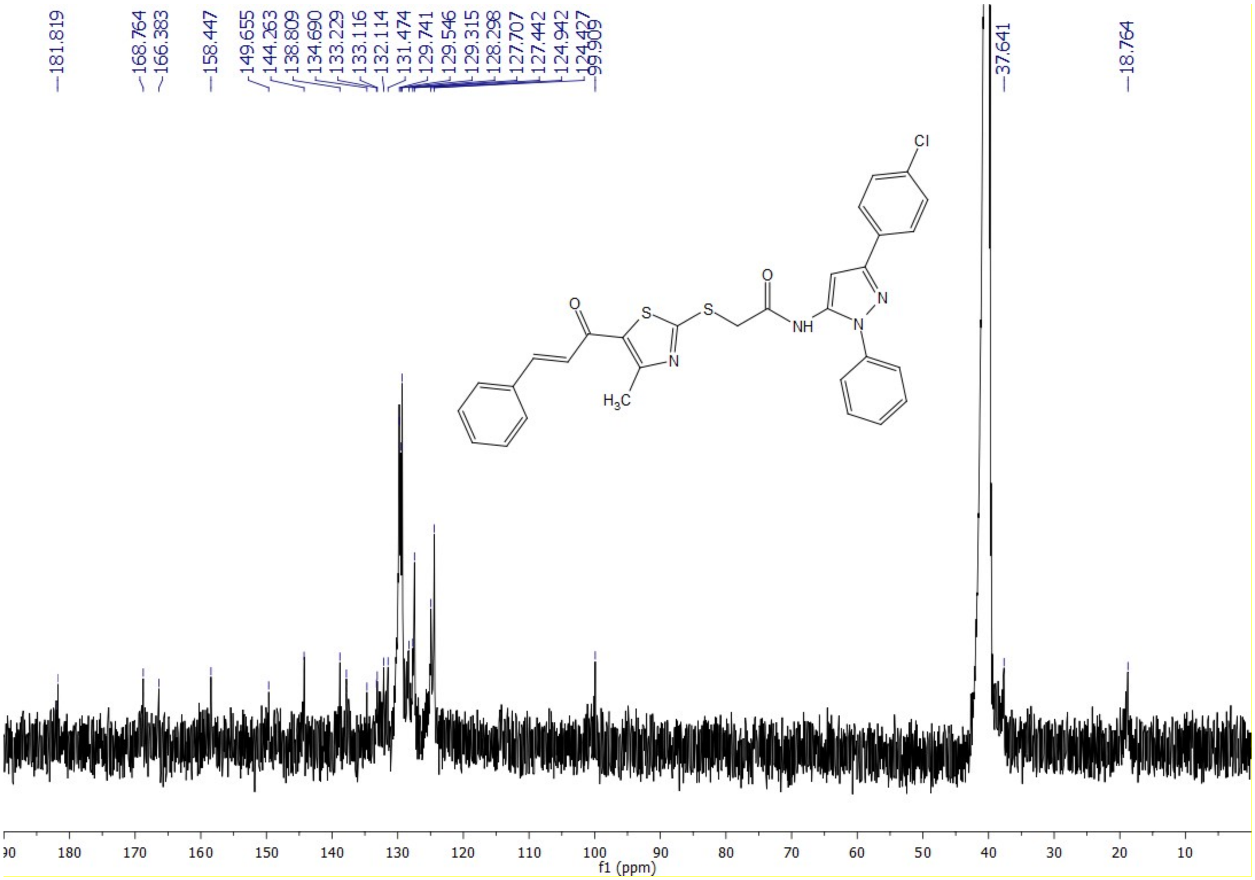

Figure S17. ^{13}C NMR spectrum of compound **9f**

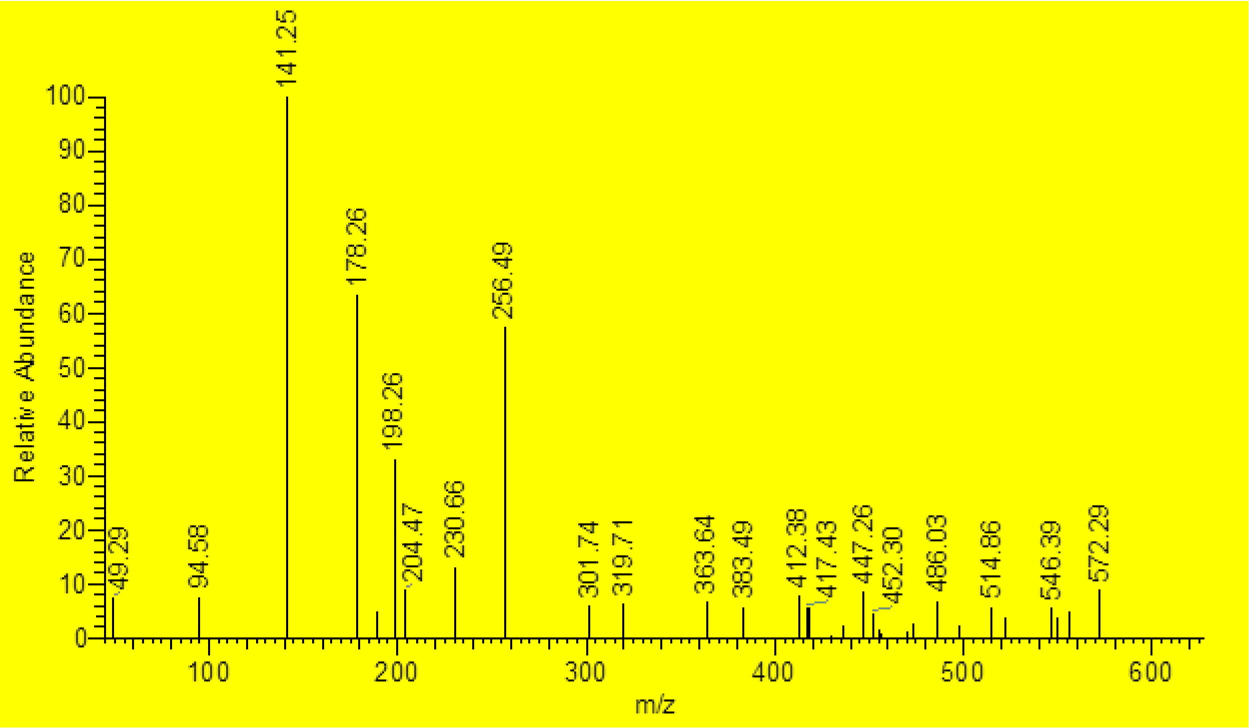

Figure S18. ESI-MS spectrum of compound **9f**

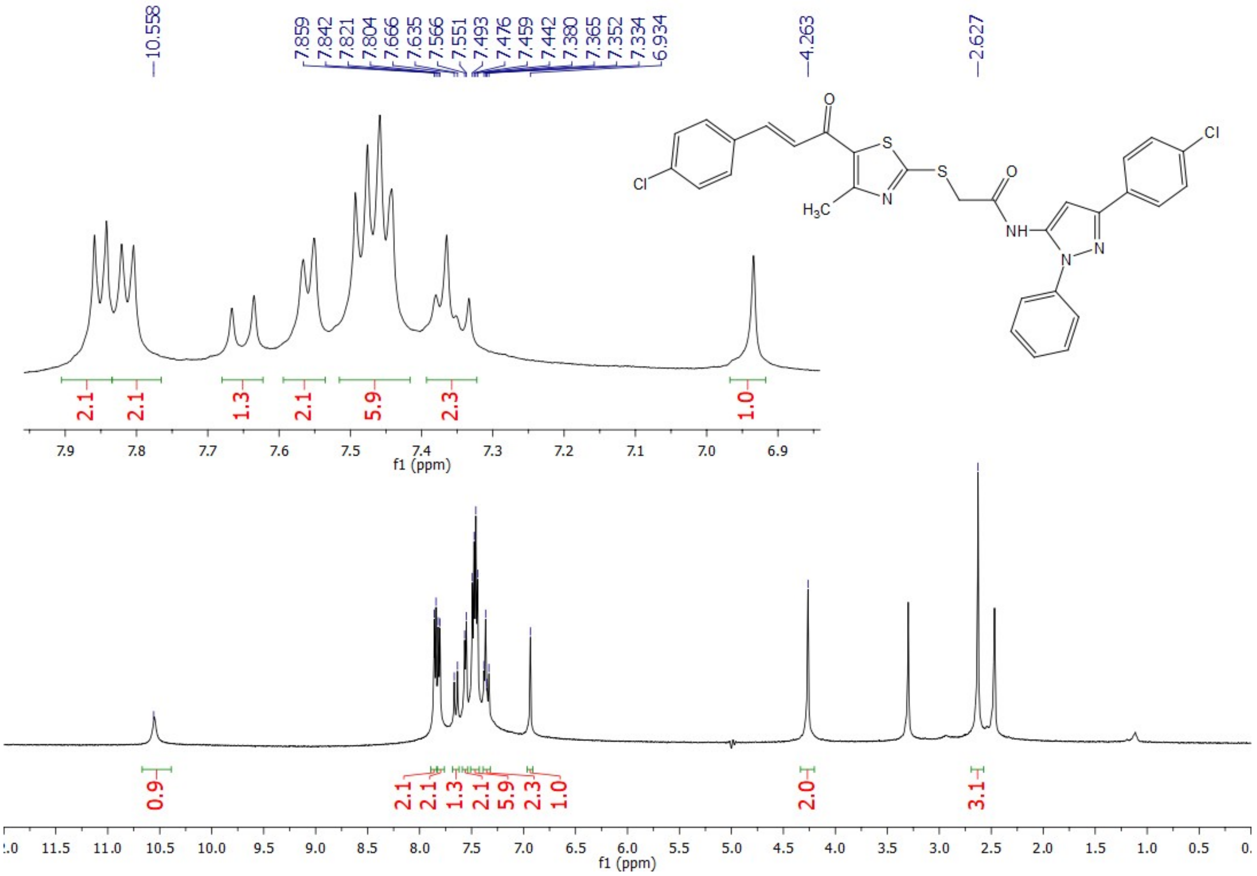

Figure S19. ^{1}H NMR spectrum of compound **9g**

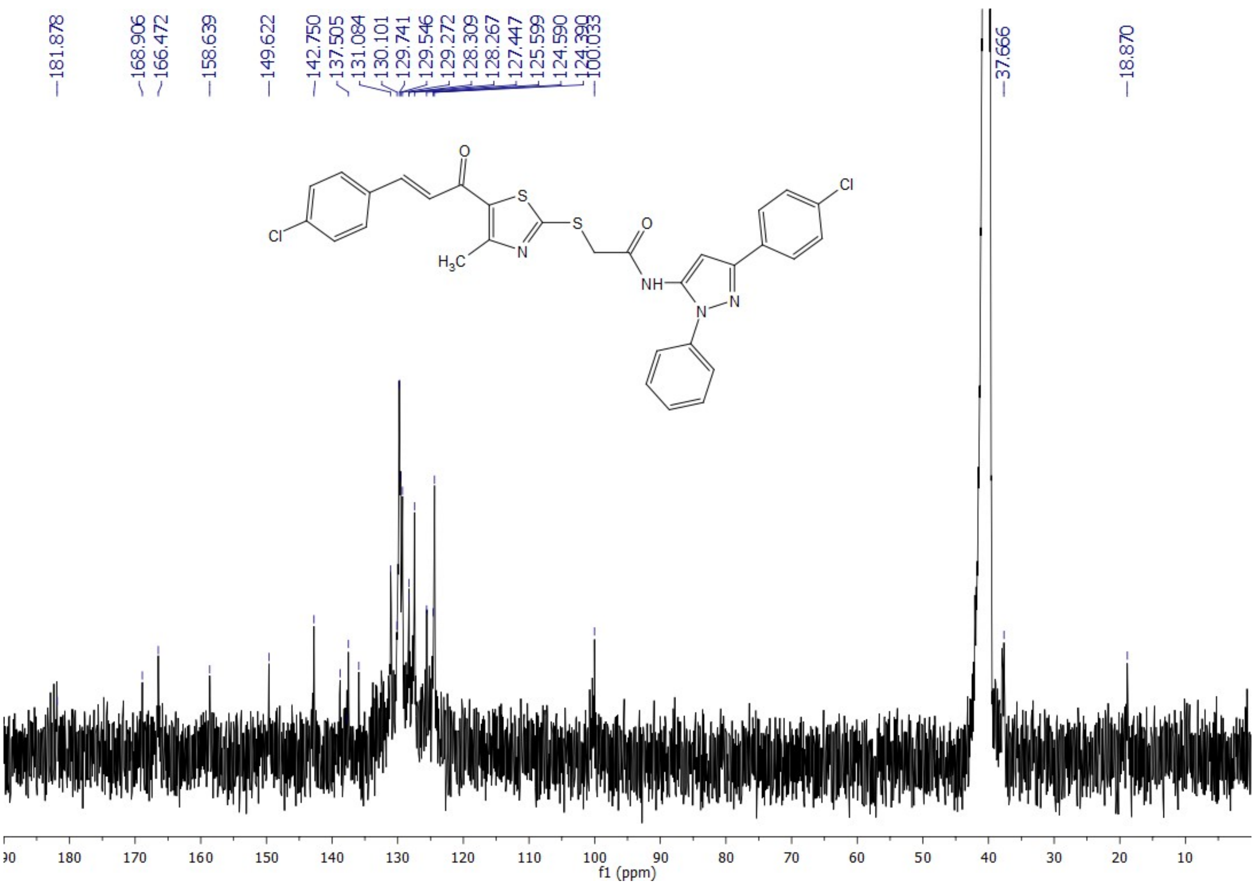

Figure S20. ^{13}C NMR spectrum of compound **9g**

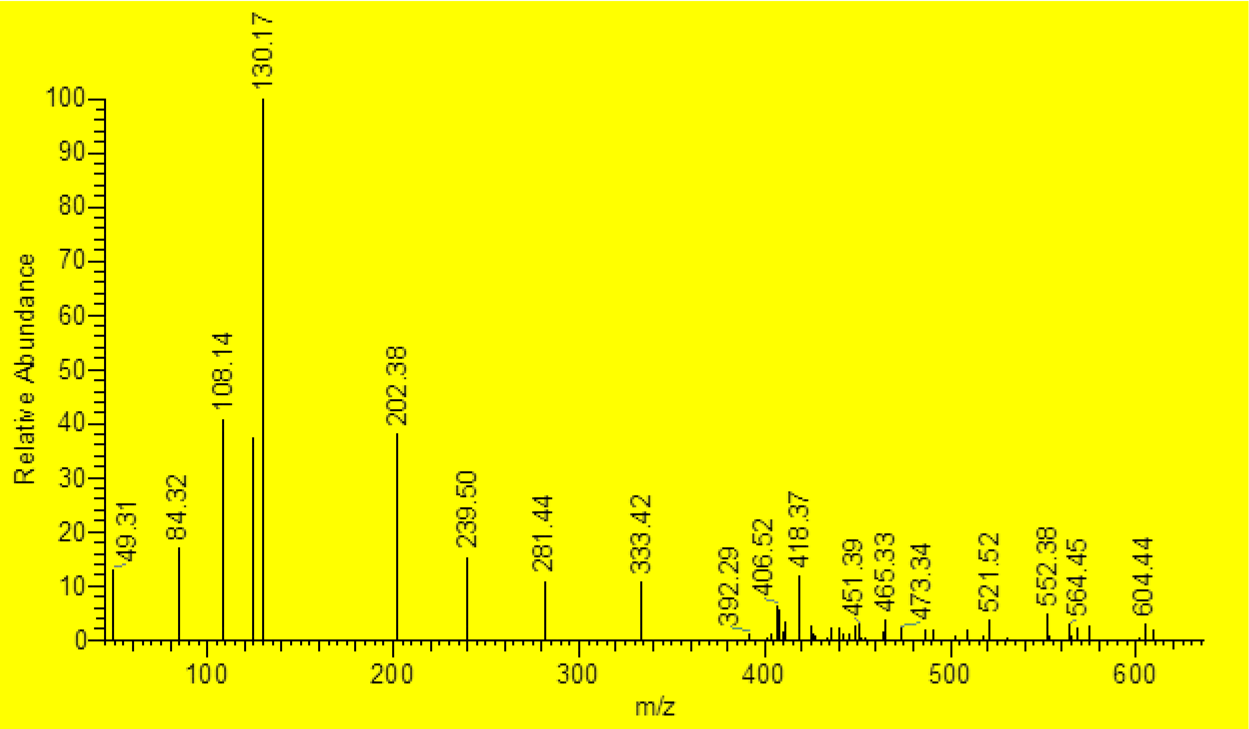

Figure S21. ESI-MS spectrum of compound **9g**

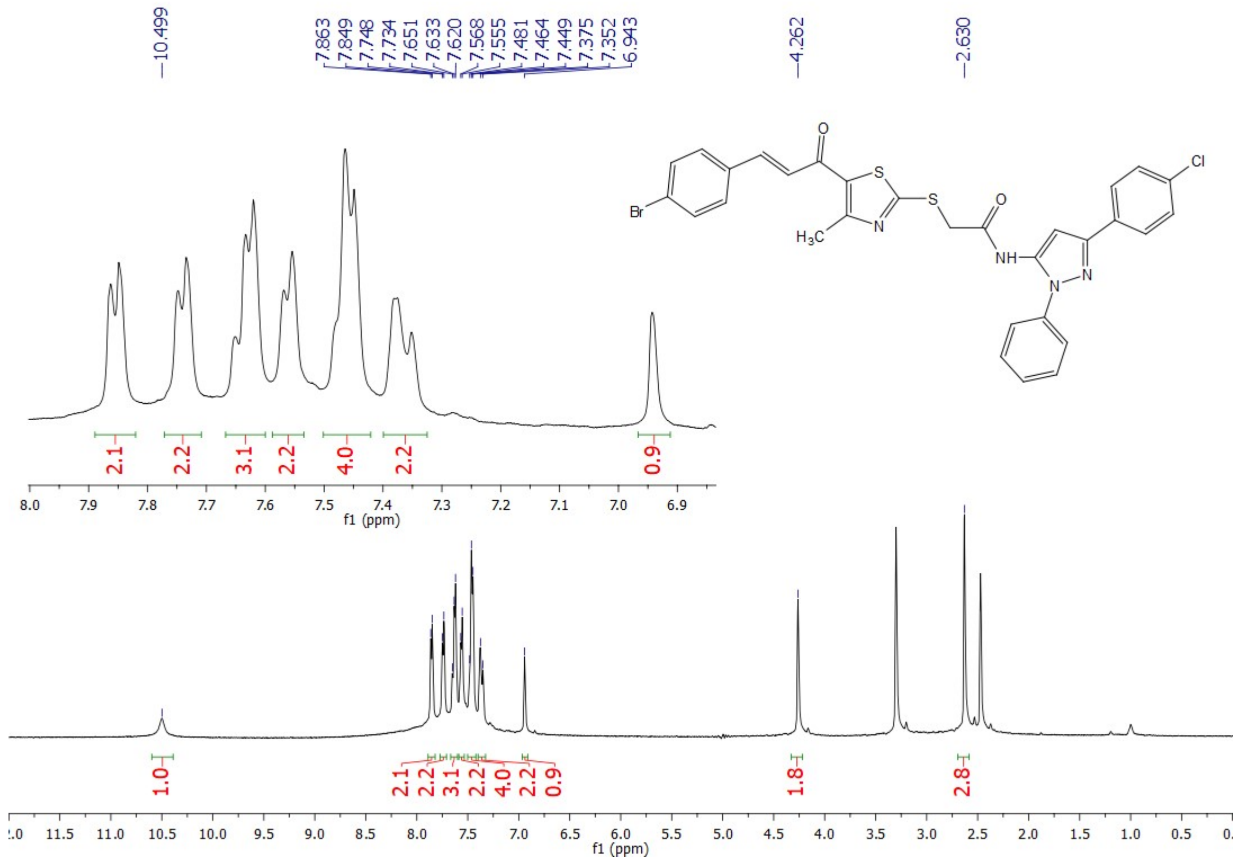

Figure S22. ^{1}H NMR spectrum of compound **9h**

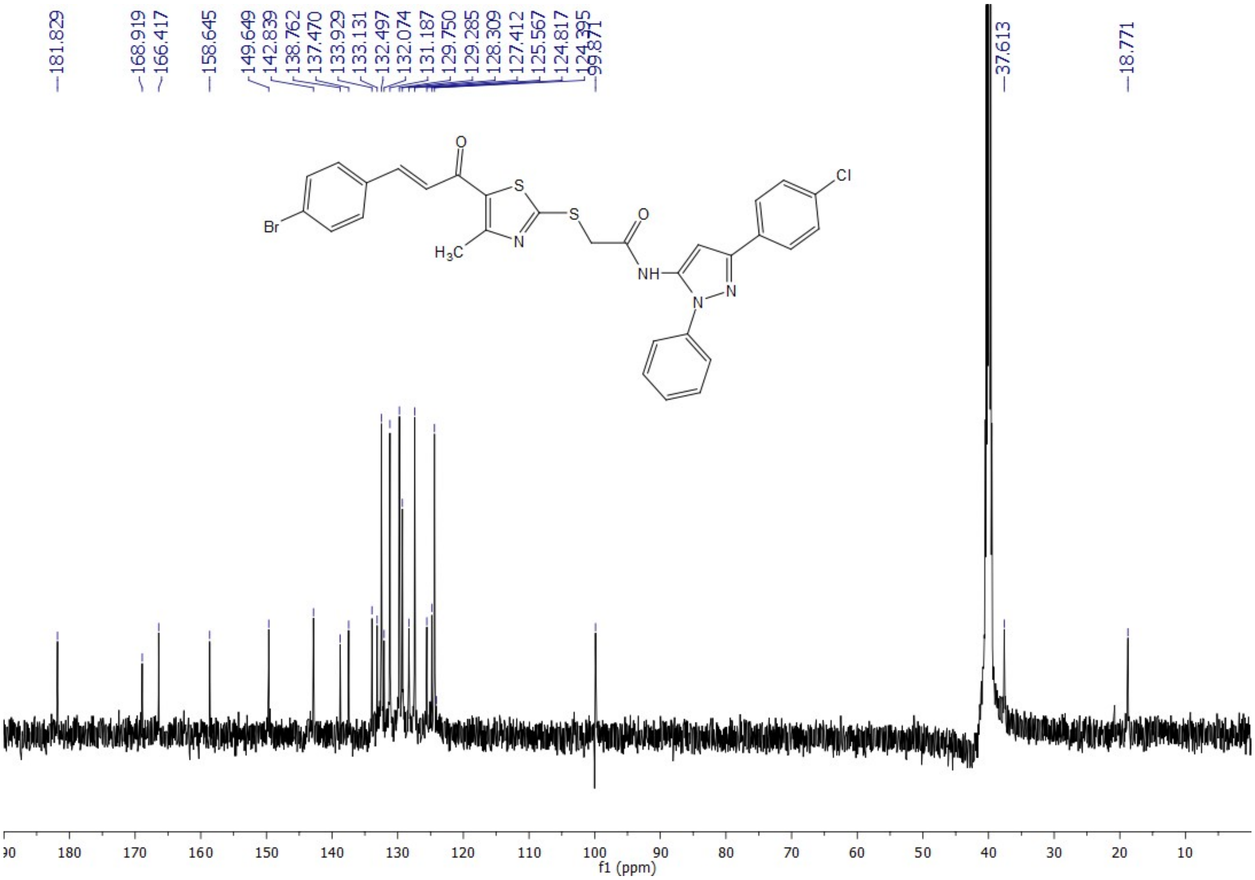

Figure S23. ^{13}C NMR spectrum of compound **9h**

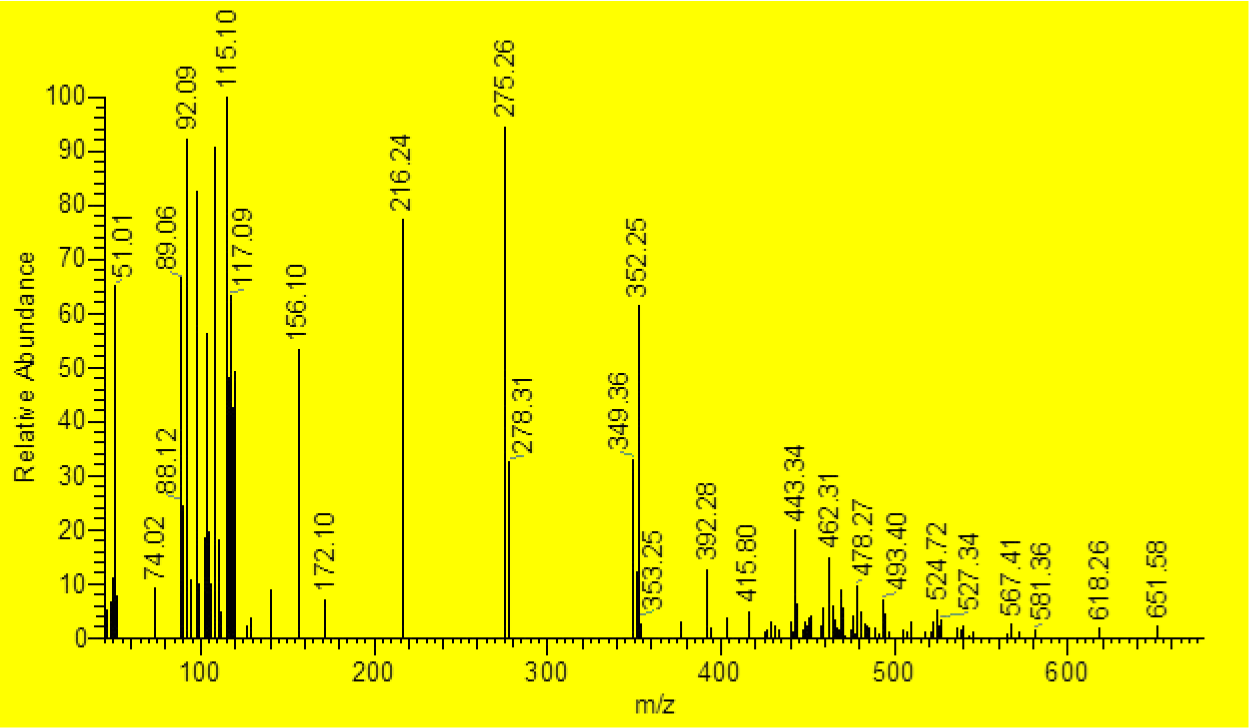

Figure S24. ESI-MS spectrum of compound **9h**

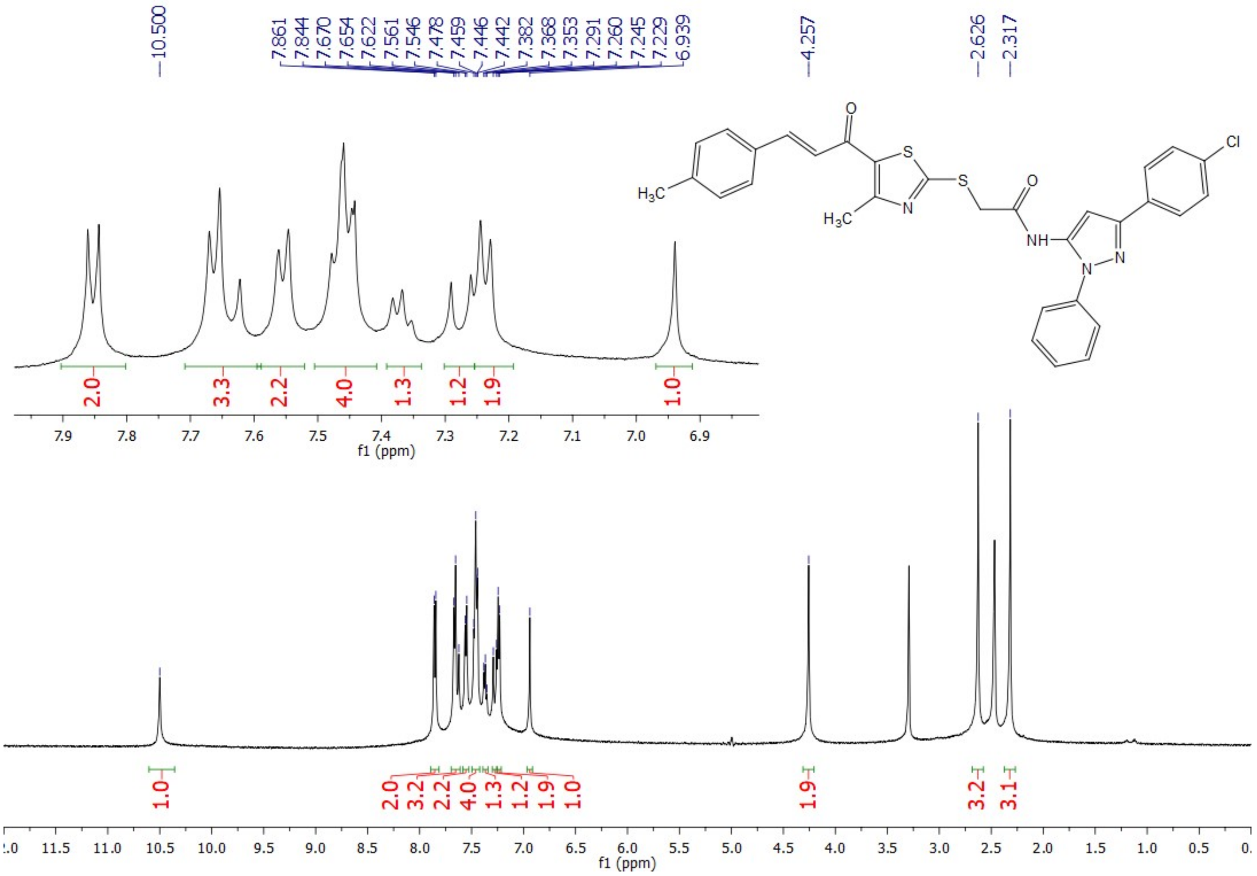

Figure S25. ^{1}H NMR spectrum of compound **9i**

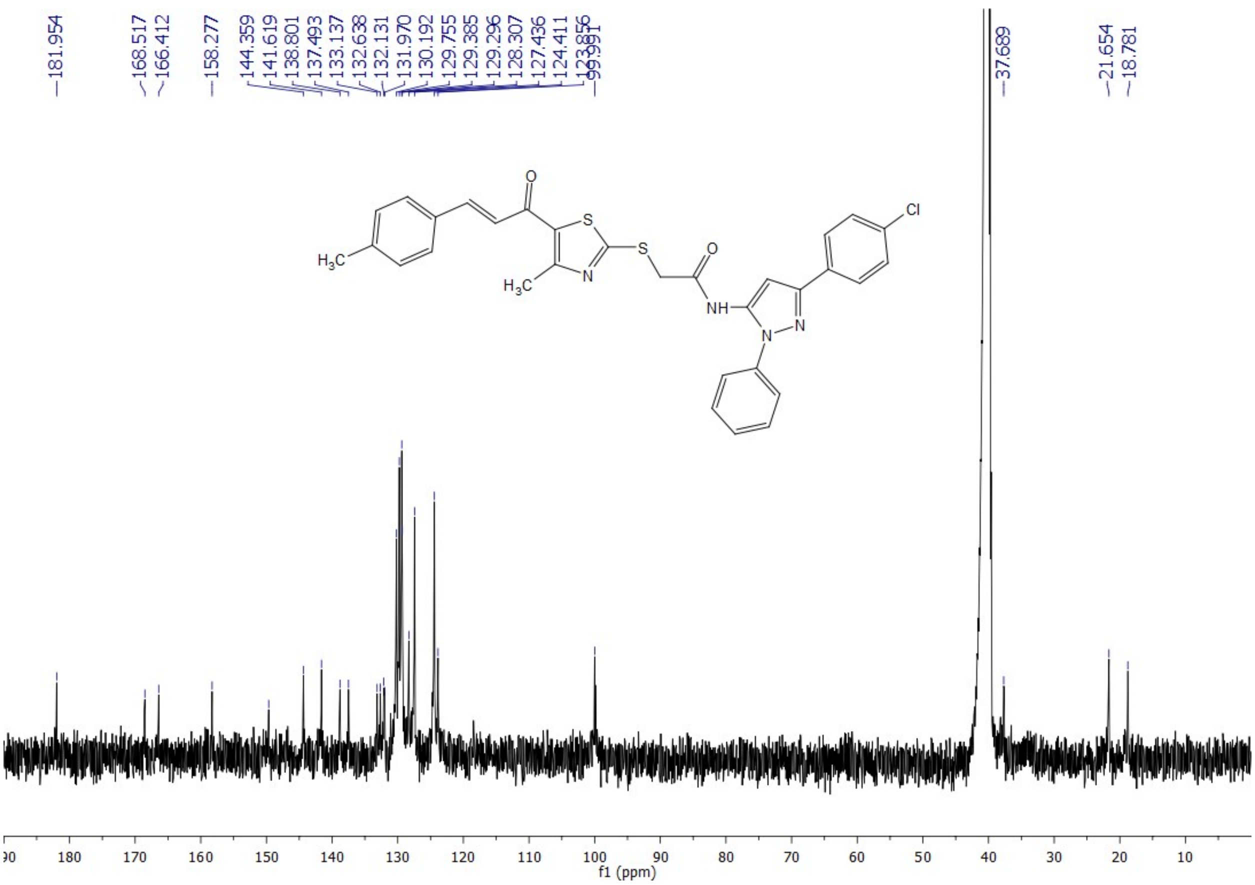

Figure S26. ^{13}C NMR spectrum of compound **9i**

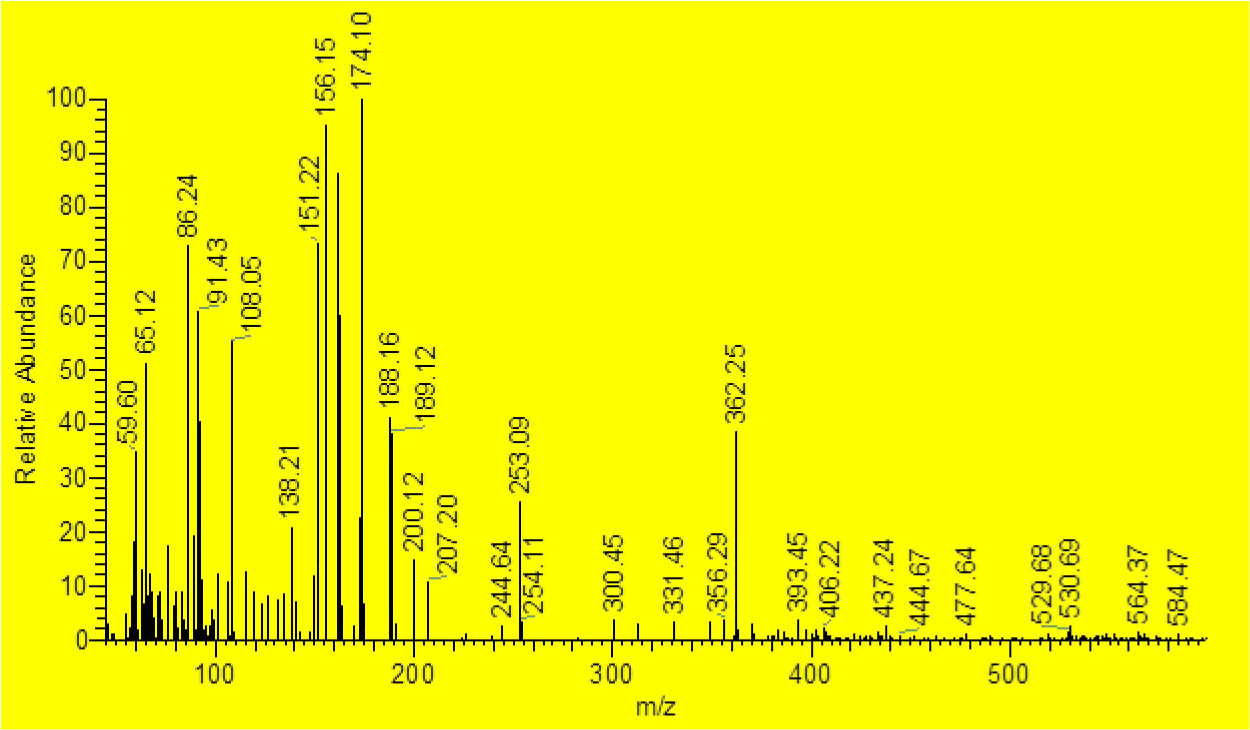

Figure S27. ESI-MS spectrum of compound **9i**

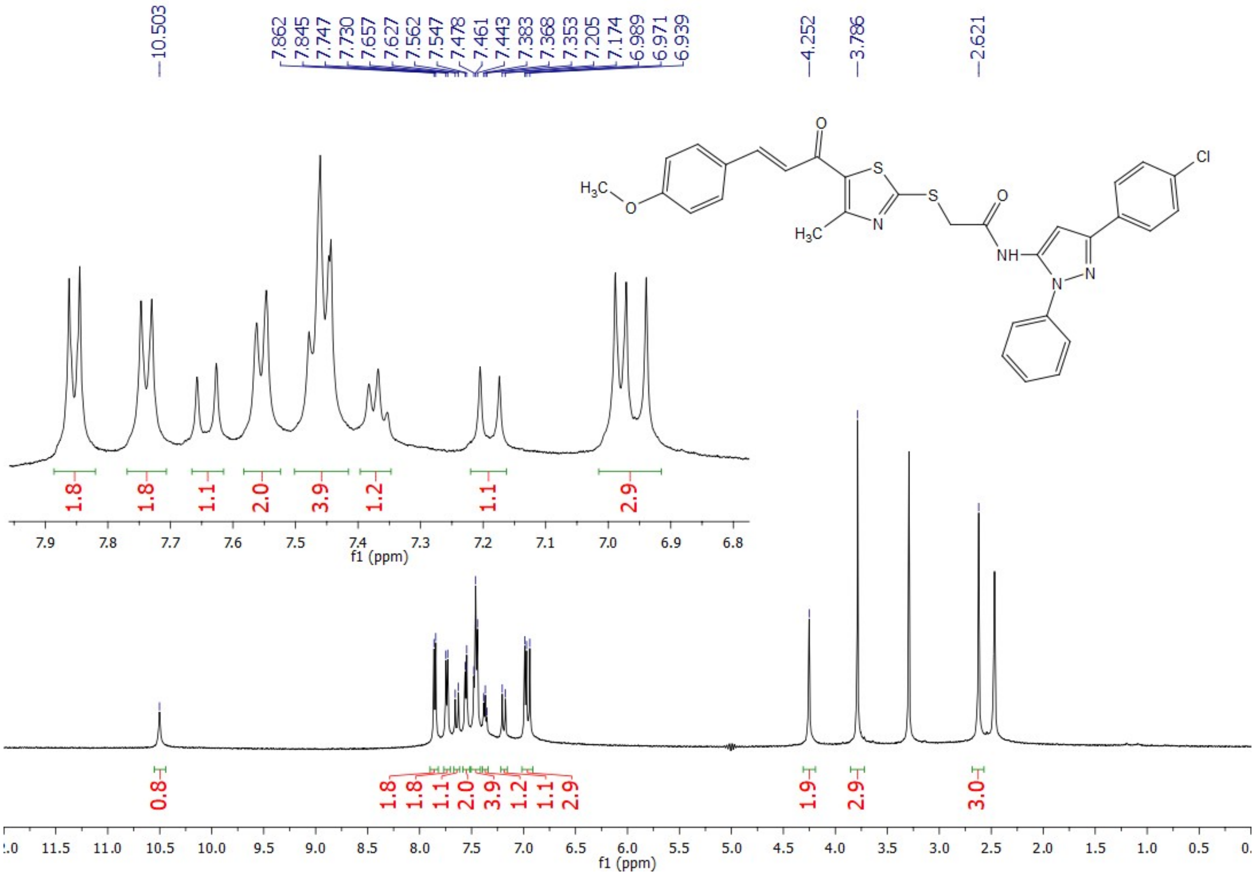

Figure S28. ^{1}H NMR spectrum of compound **9j**

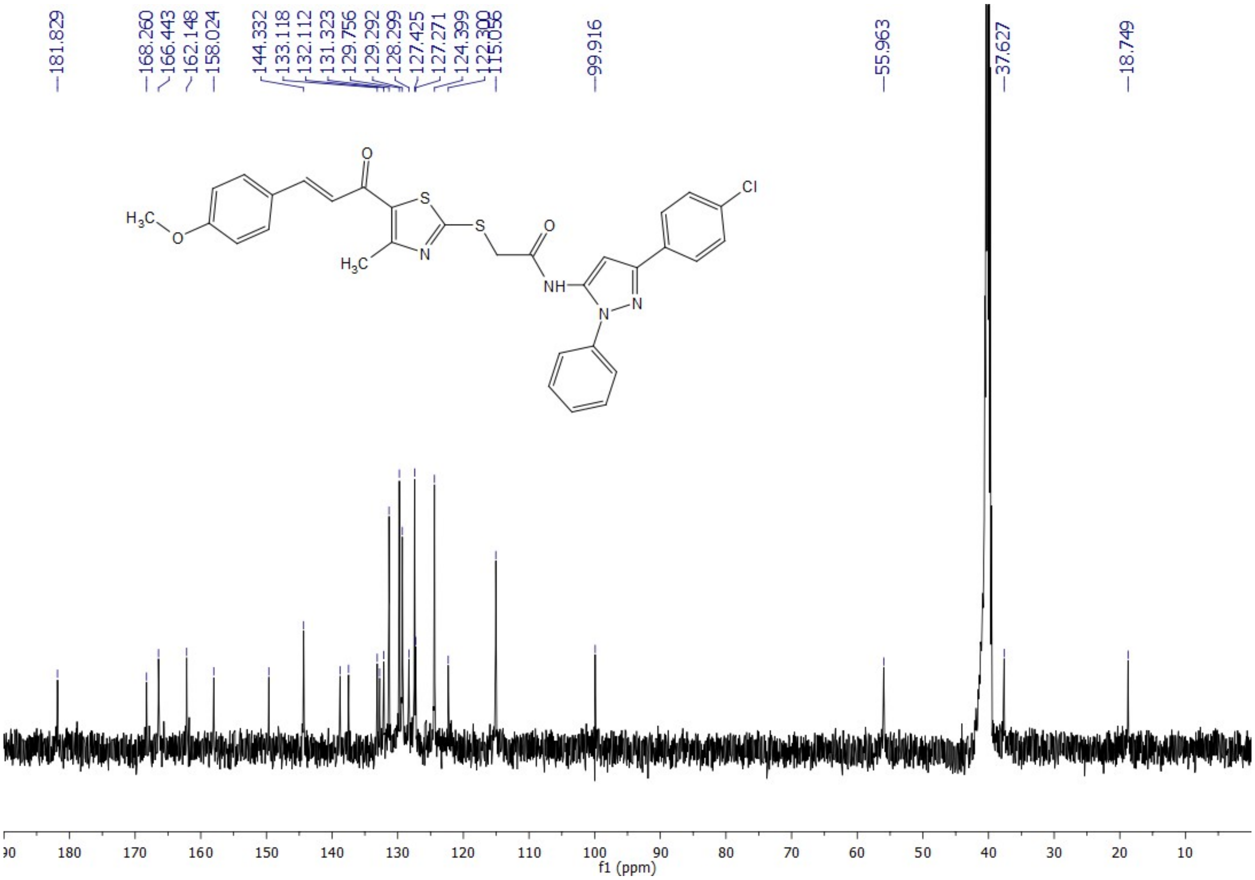

Figure S29. ^{13}C NMR spectrum of compound **9j**

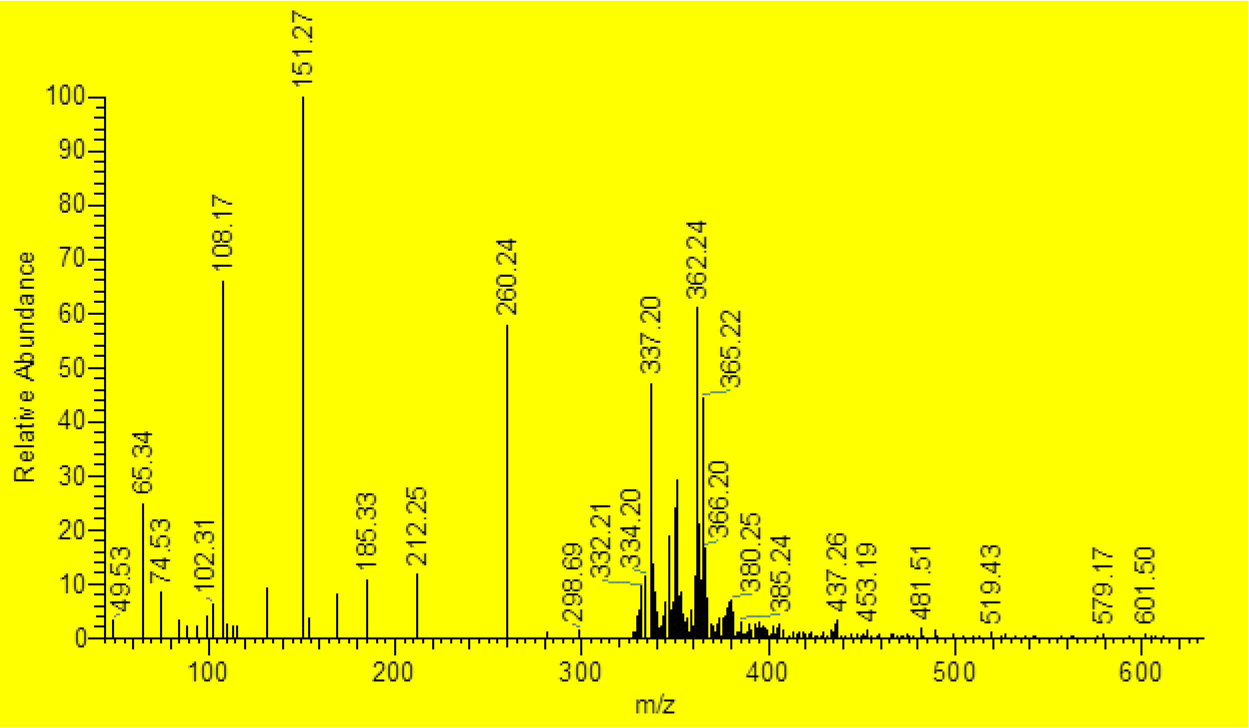

Figure S30. ESI-MS spectrum of compound **9j**

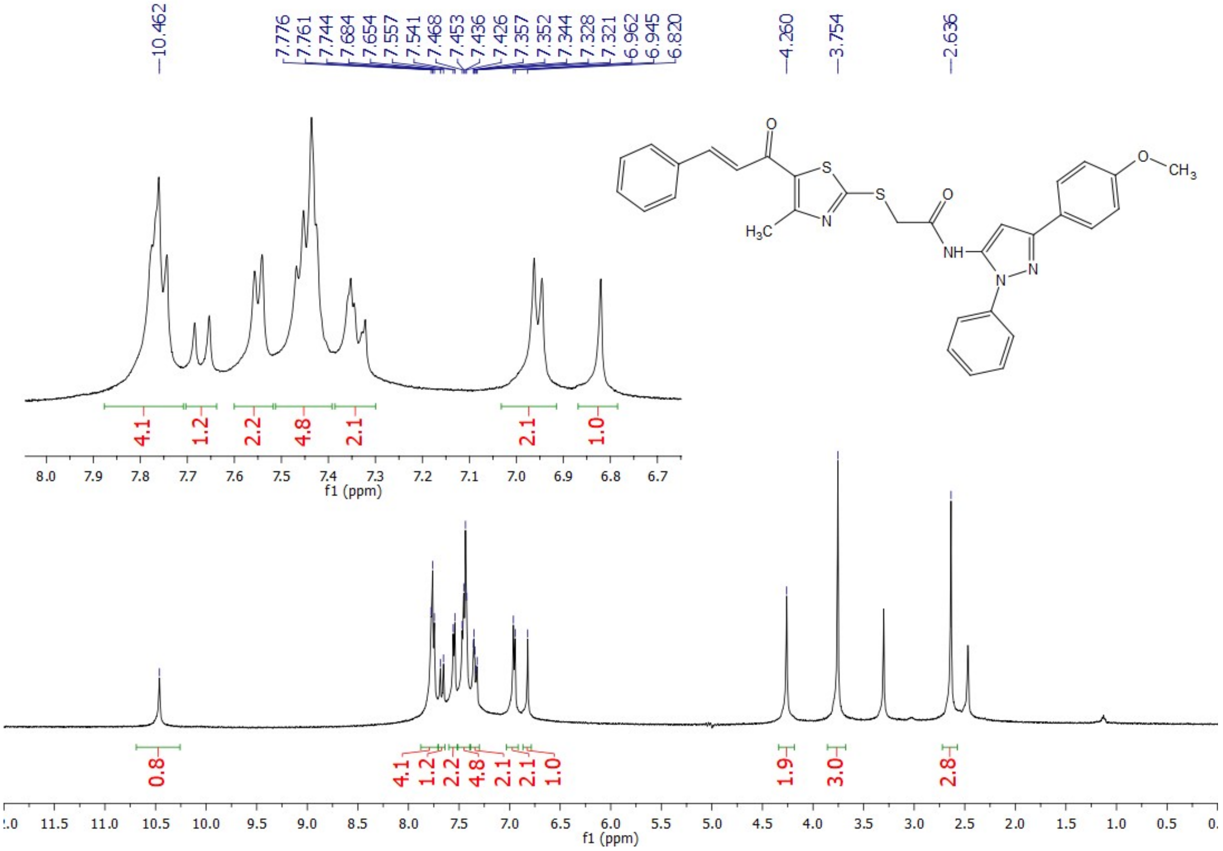

Figure S31. ^{1}H NMR spectrum of compound **9k**

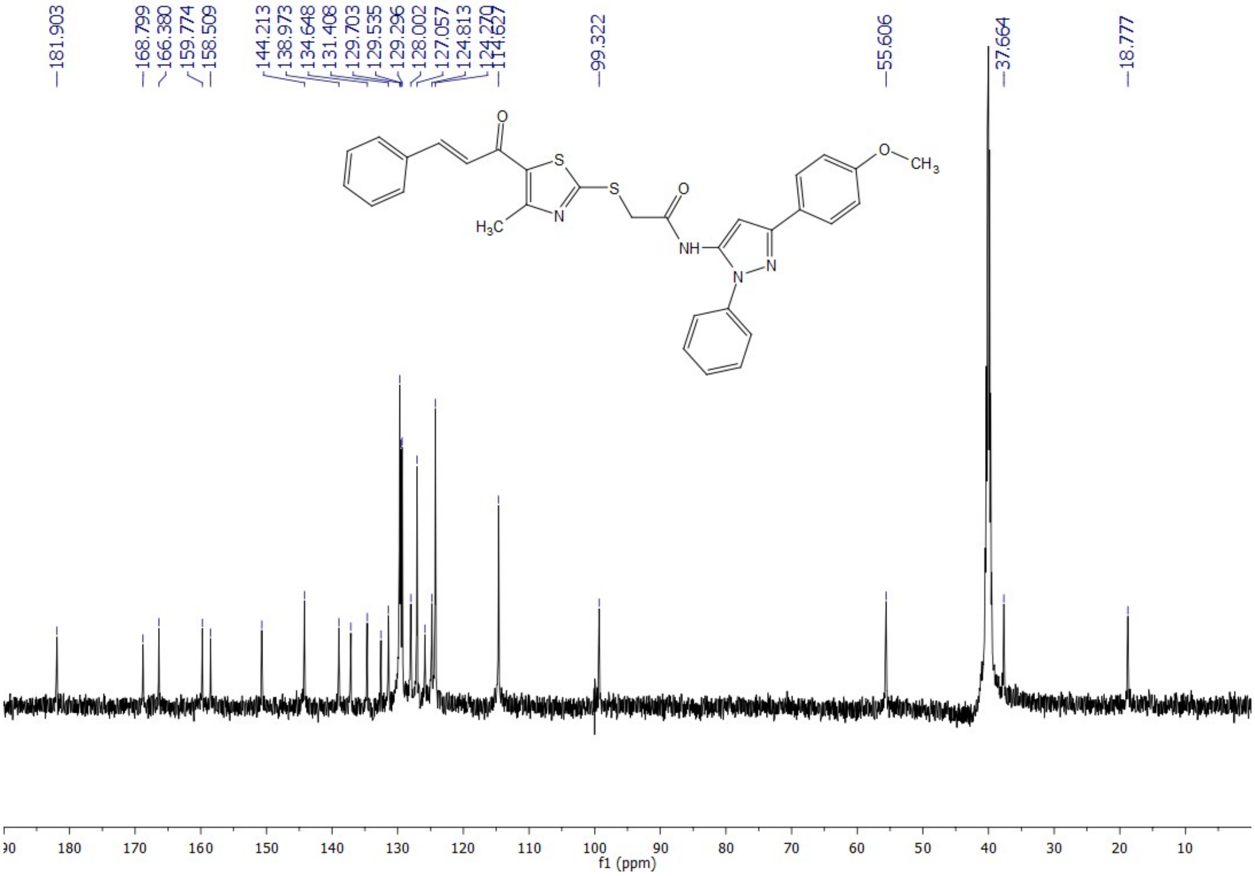

Figure S32. ^{13}C NMR spectrum of compound **9k**

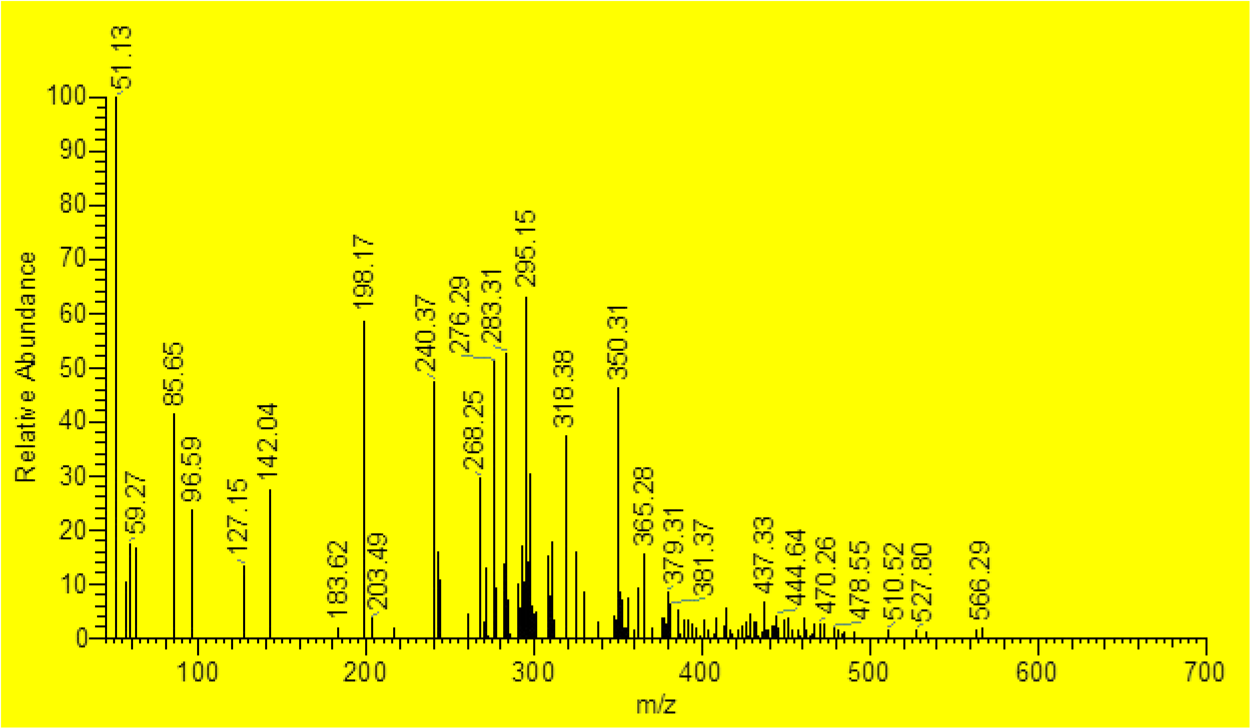

Figure S33. ESI-MS spectrum of compound **9k**

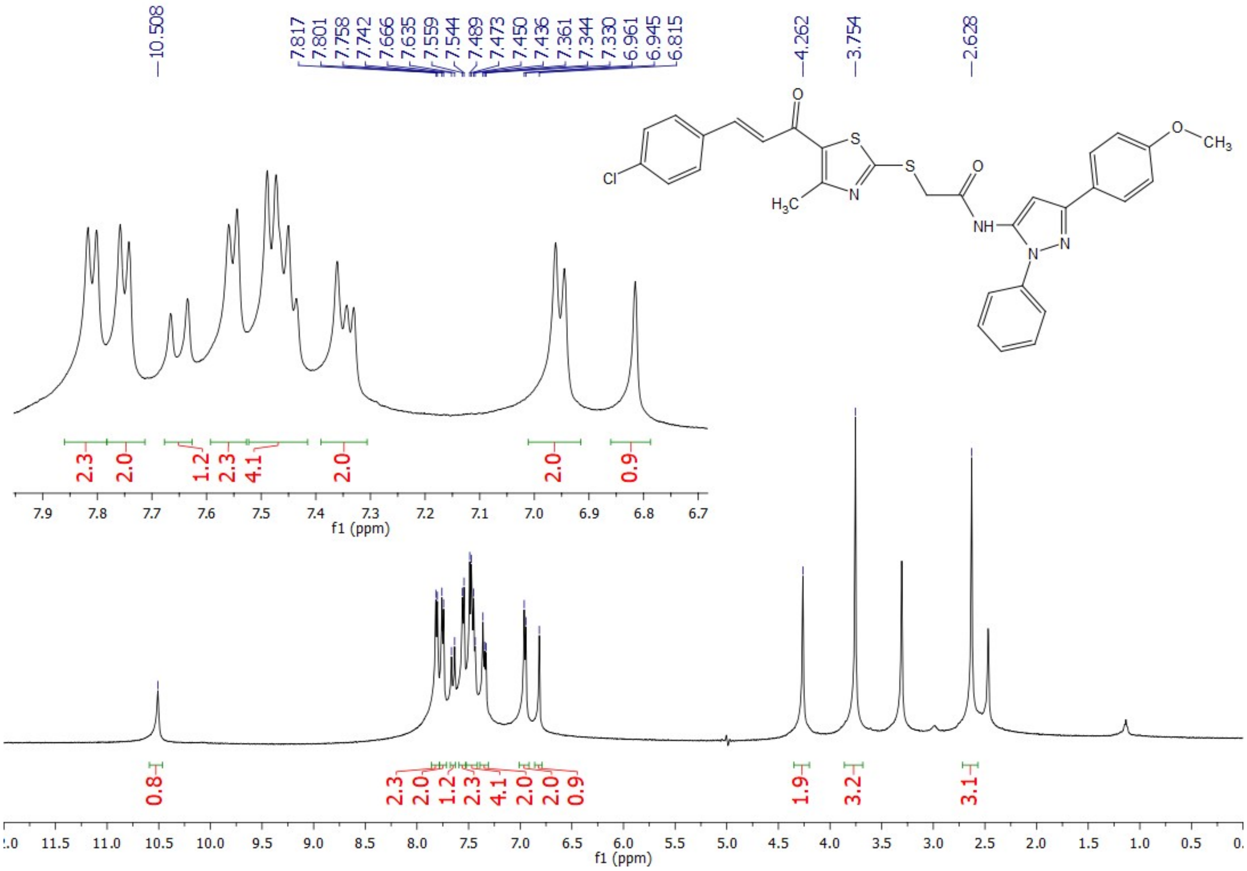

Figure S34. ^{1}H NMR spectrum of compound **9l**

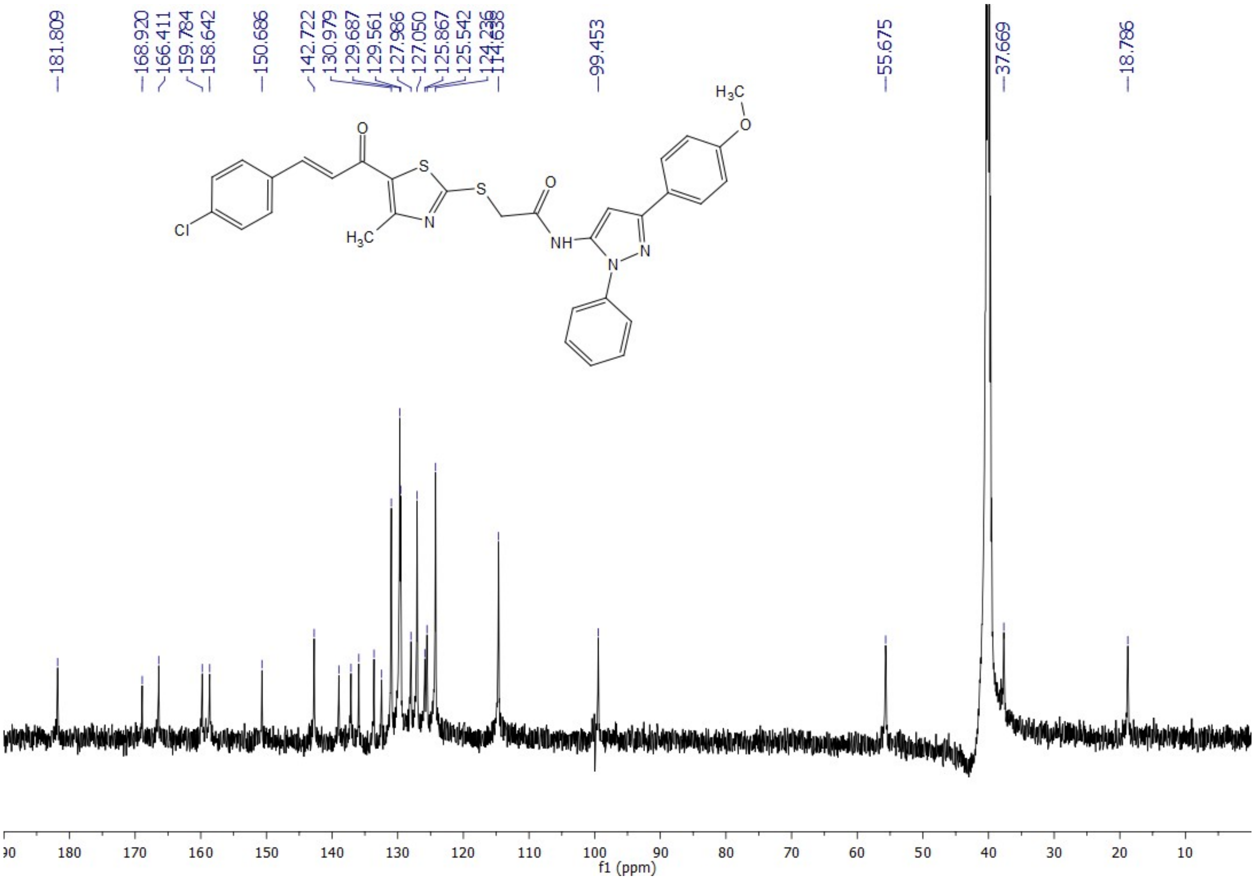

Figure S35. ^{13}C NMR spectrum of compound **9l**

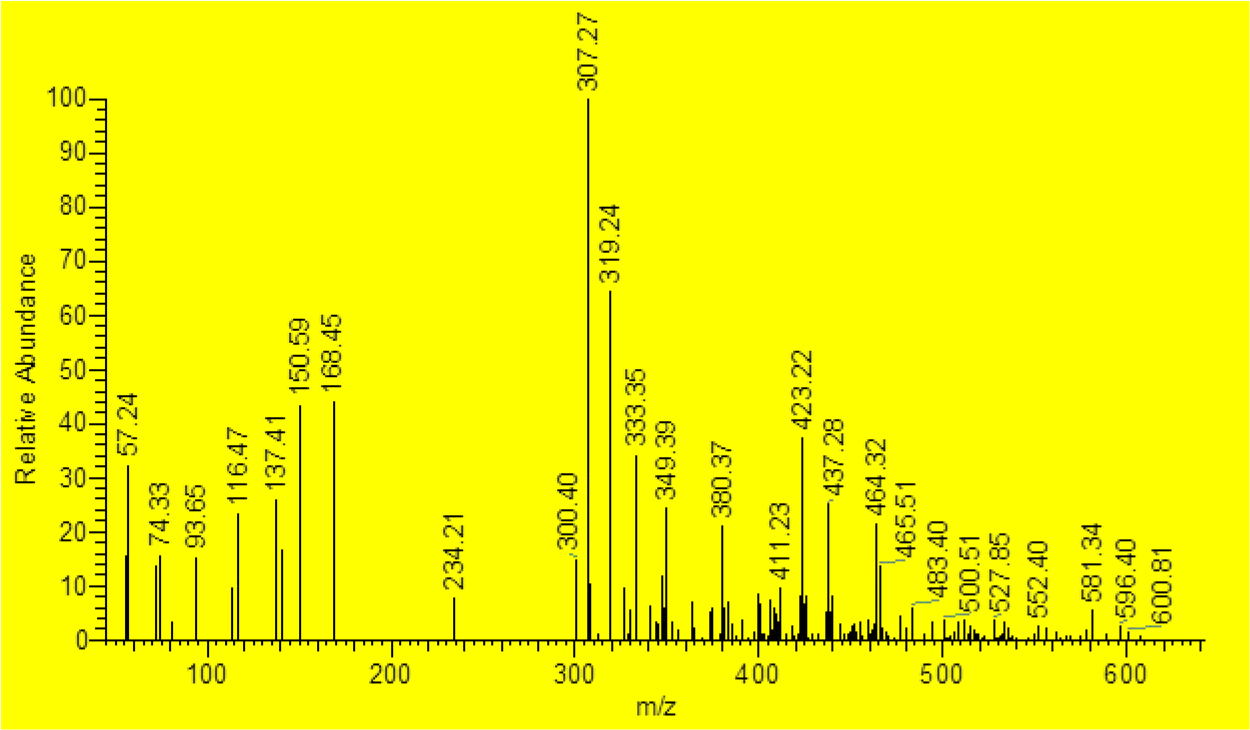

Figure S36. ESI-MS spectrum of compound **9l**

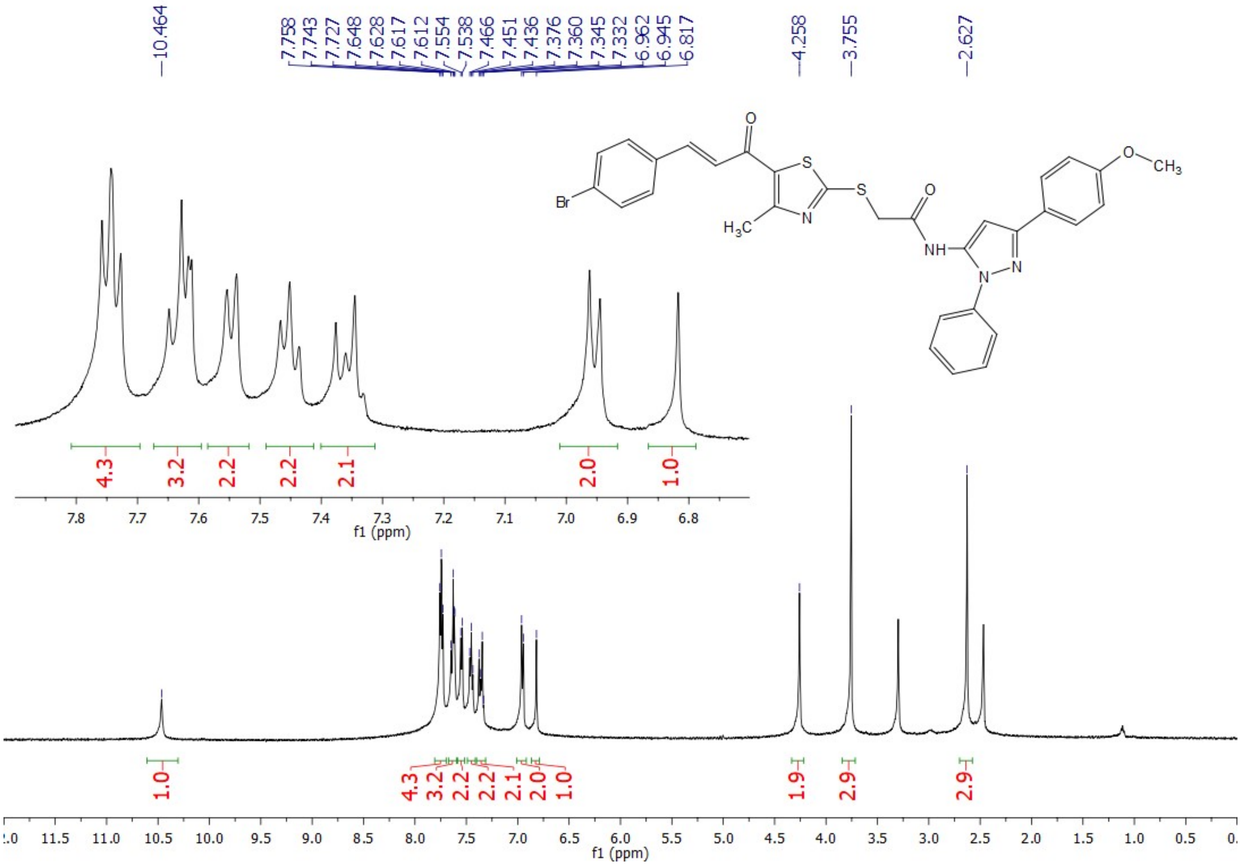

Figure S37. ^{1}H NMR spectrum of compound **9m**

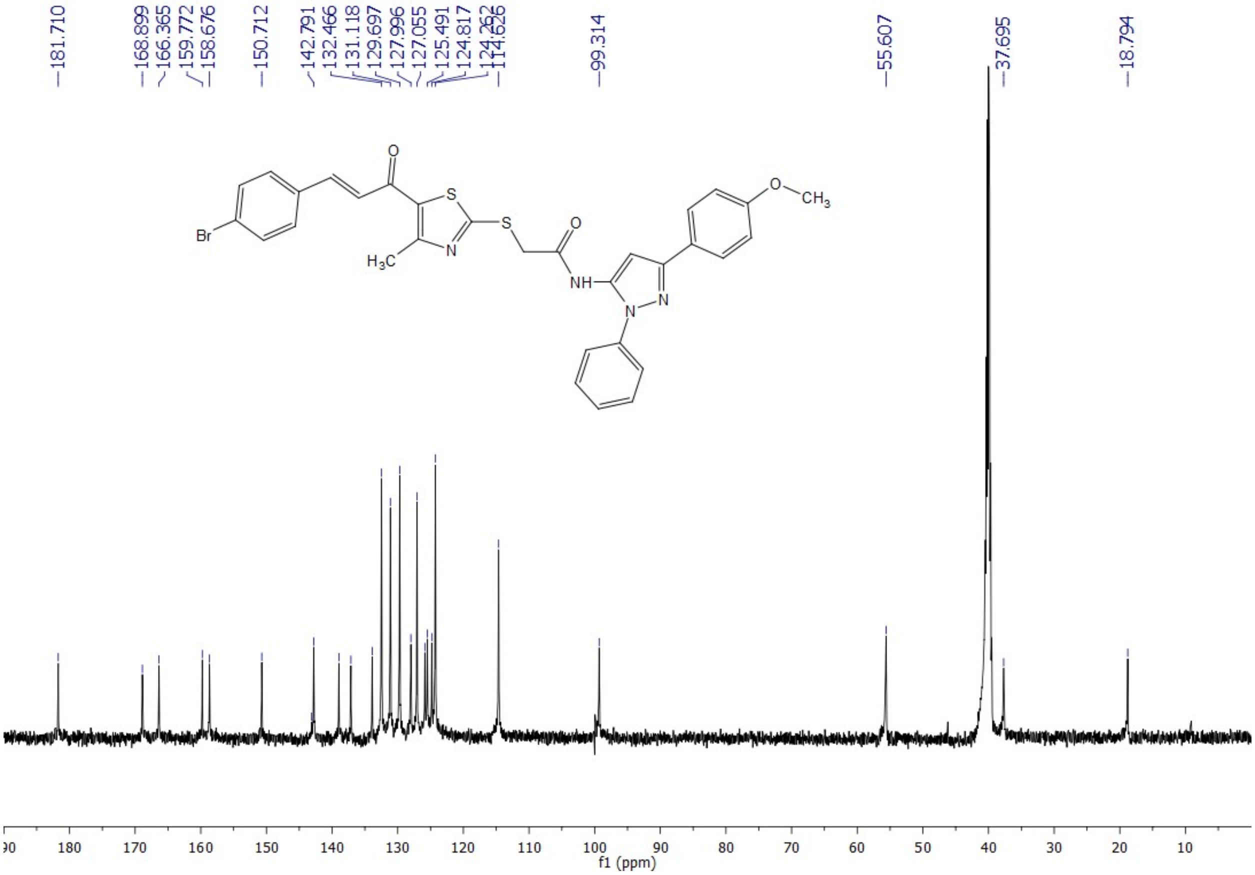

Figure S38. ^{13}C NMR spectrum of compound **9m**

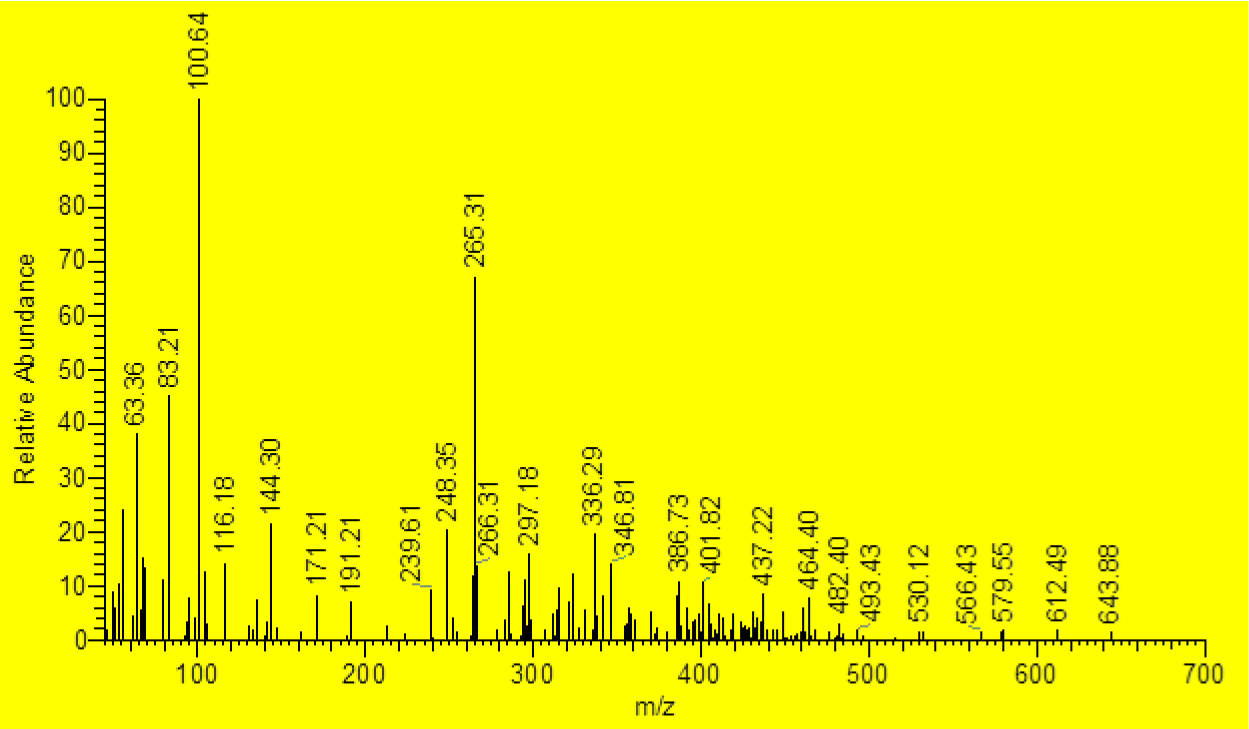

Figure S39. ESI-MS spectrum of compound **9m**

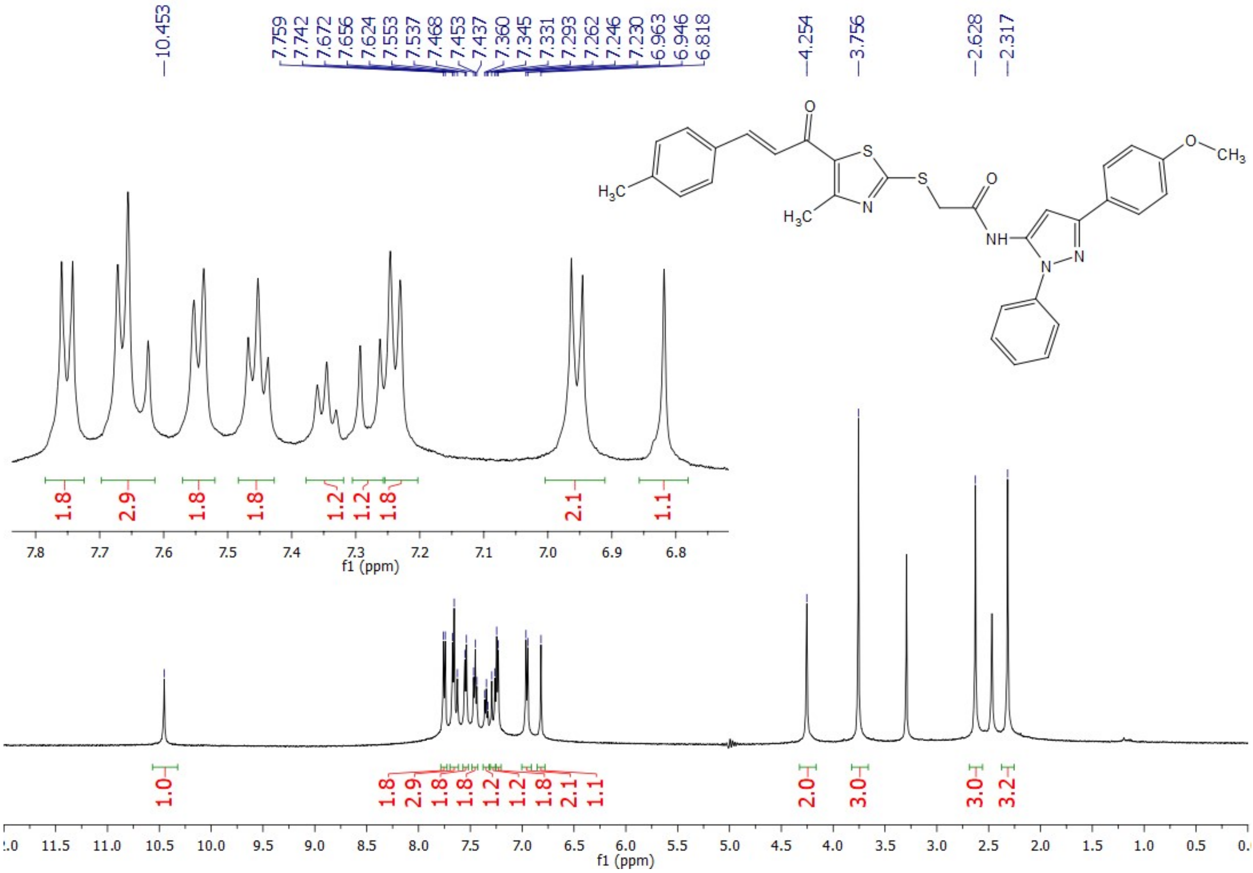

Figure S40. ^{1}H NMR spectrum of compound **9n**

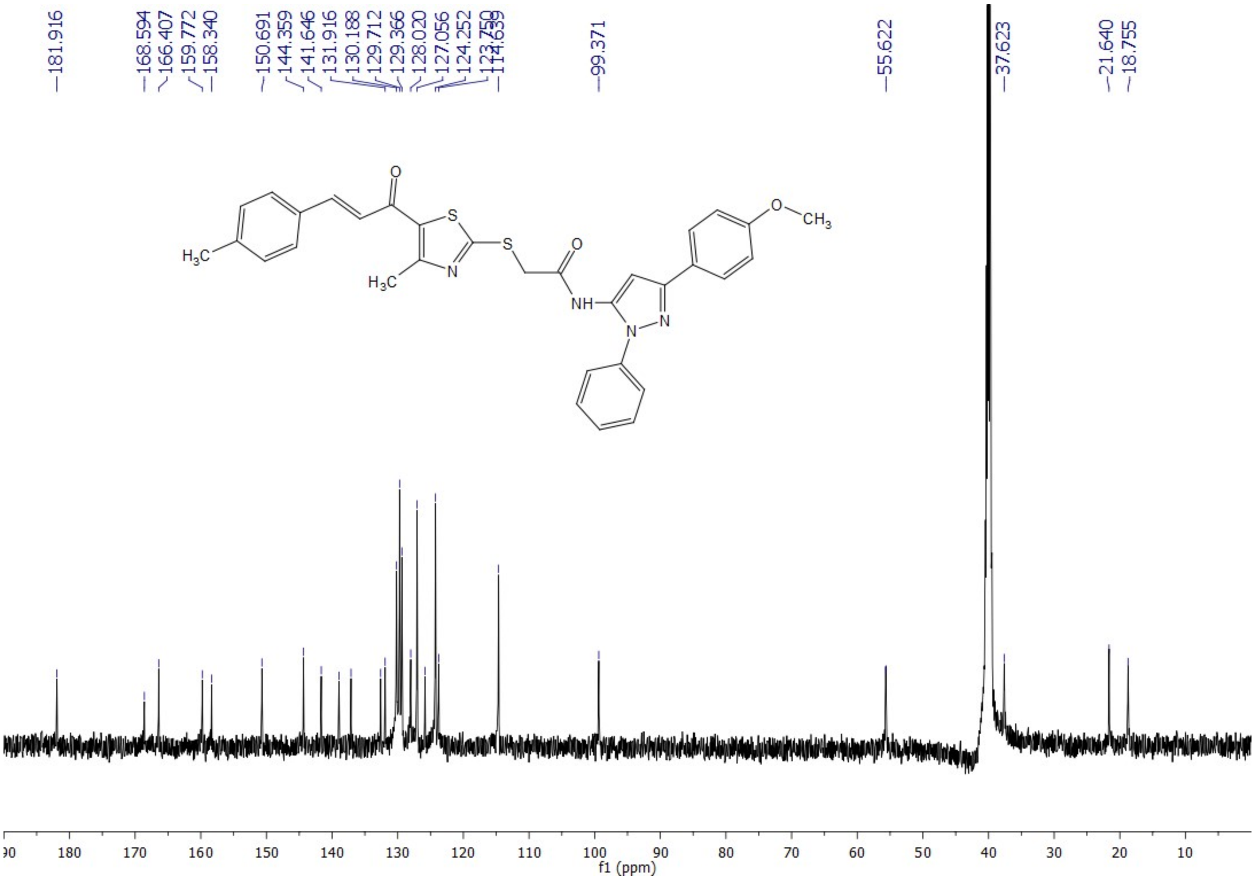

Figure S42. ^{13}C NMR spectrum of compound **9n**

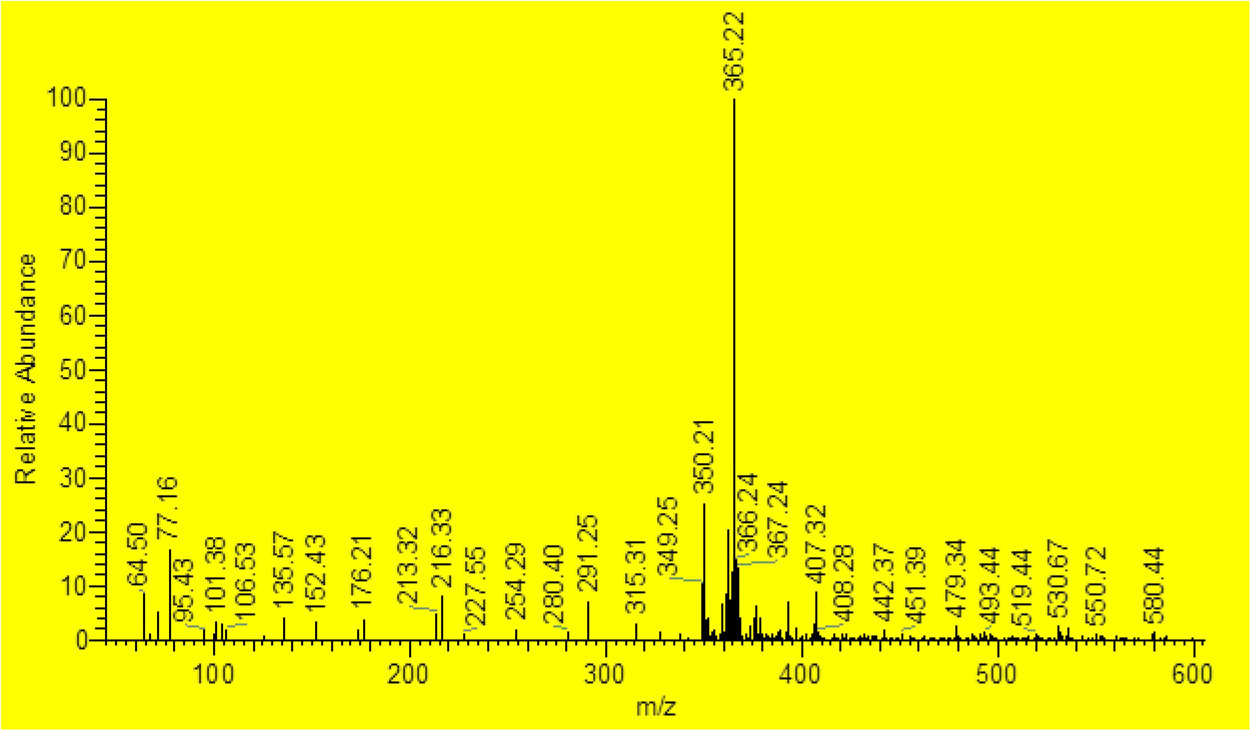

Figure S42. ESI-MS spectrum of compound **9n**

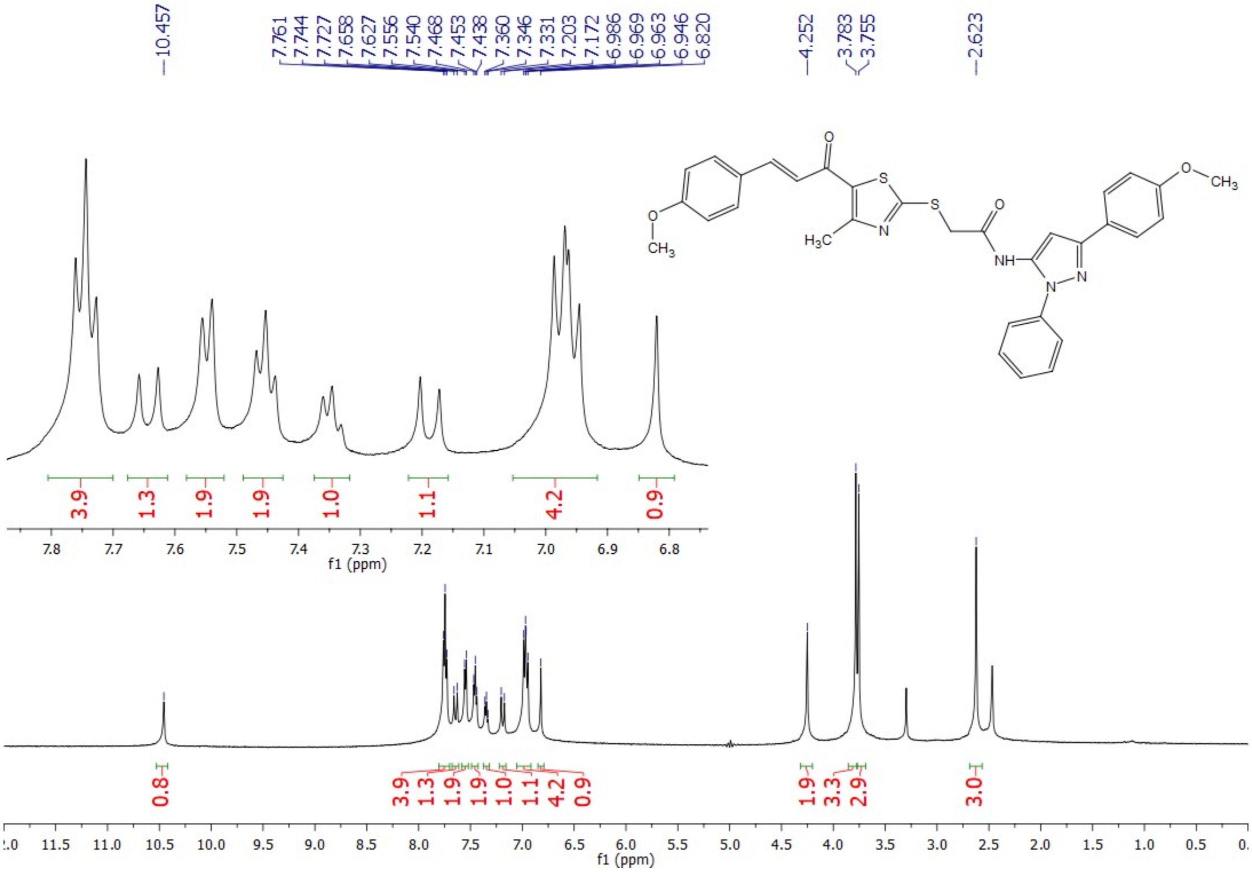

Figure S43. ^{1}H NMR spectrum of compound **9o**

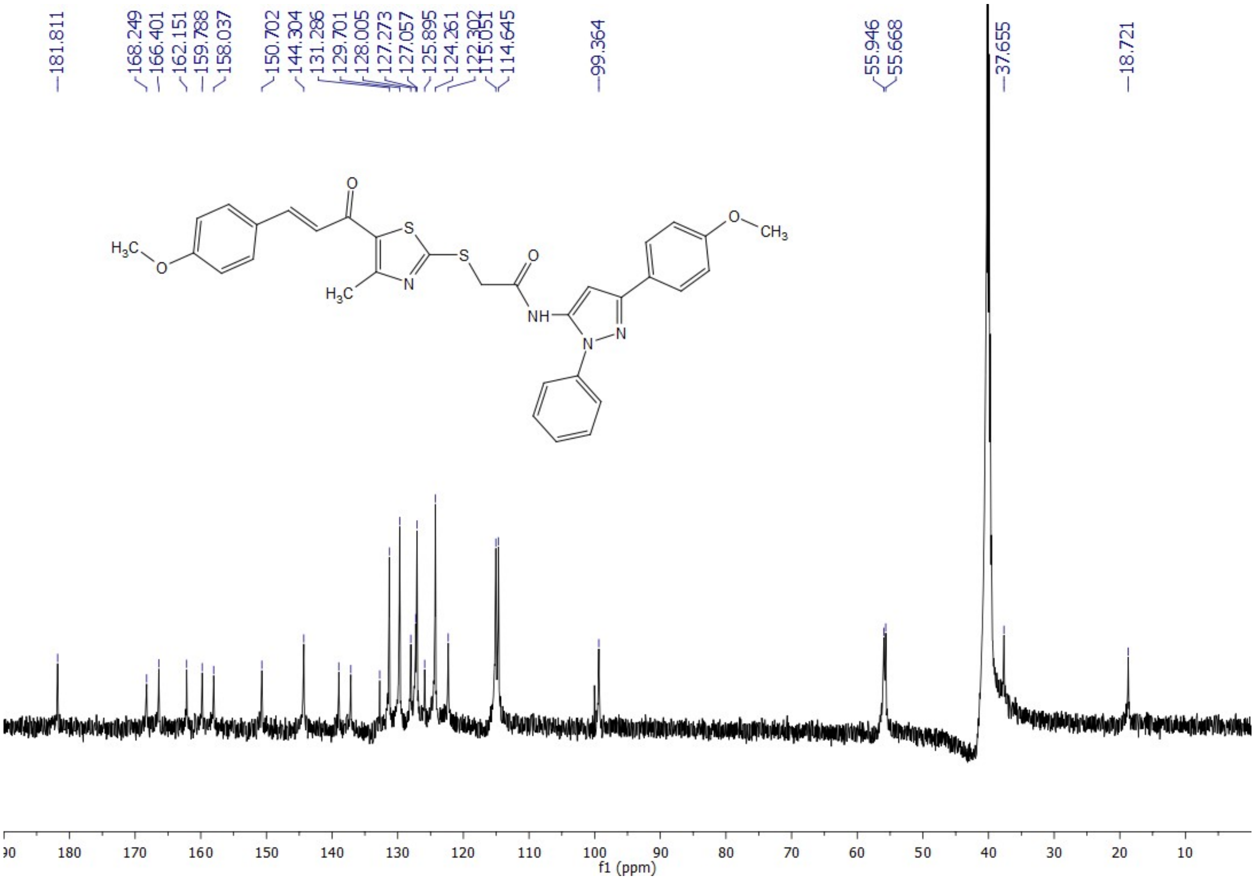

Figure S44. ^{13}C NMR spectrum of compound **9o**

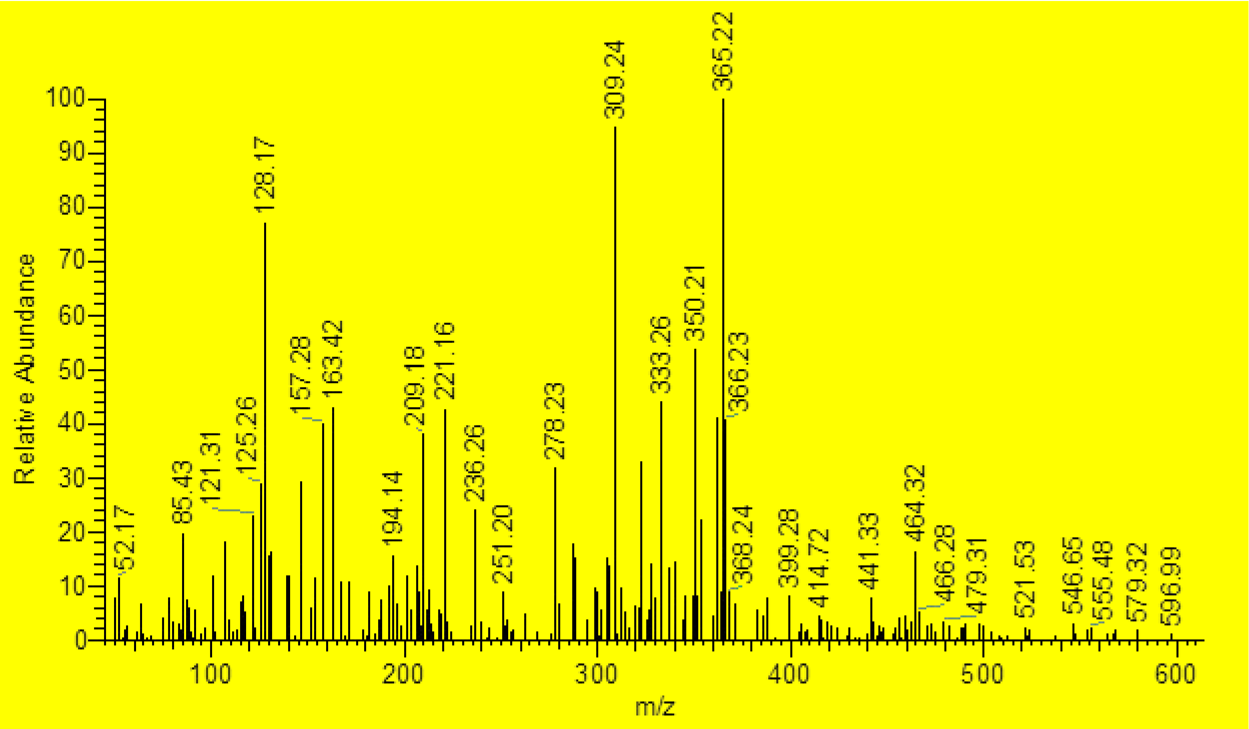

Figure S45. ESI-MS spectrum of compound **9o**

Table S1. Docking interactions of compound **9l**

Compound	Target Protein	Binding Affinity (kcal/mol)	Hydrogen Bonding	Hydrophobic Interactions
**9l**	Tubulin	-9.1	Val238 (classical H-bond); Tyr52 and Gln136 (carbon-hydrogen bonds)	eu255, Leu242, Val238, Ala250, Cys241, Thr239, Leu252, Ile4, Ala316, Ala354, Lys352, Met259, Val181, Phe404, Ile347
	COX-2		Tyr355 (classical H-bond); Arg120 (π-donor non-classical H-bond)	Val349, Ala527, Val523, Val116, Leu359, Leu531, Gly526, Leu384, Tyr385, Phe381, Trp387, Ala516, Ile517, Phe518, His90, Pro514, His95

Table S2. Physicochemical Properties of compound **9l**

Property	9l	Comment
Molecular Weight	599.11	Contain hydrogen atoms. Optimal:100~600
Volume	588.652	Van der Waals volume
Density	1.018	Density = MW / Volume
nHA	6.0	Number of hydrogen bond acceptors. Optimal:0~12
nHD	0.0	Number of hydrogen bond donors. Optimal:0~7
nRot	11.0	Number of rotatable bonds. Optimal:0~11
nRing	5.0	Number of rings. Optimal:0~6
MaxRing	6.0	Number of atoms in the biggest ring. Optimal:0~18
nHet	9.0	Number of heteroatoms. Optimal:1~15
fChar	0.0	Formal charge. Optimal:-4 ~4
nRig	31.0	Number of rigid bonds. Optimal:0~30
Flexibility	0.355	Flexibility = nRot /nRig
Stereo Centers	0.0	Stereo Centers. Optimal: □ 2
TPSA	74.08	Topological Polar Surface Area. Optimal:0~140
logS	-7.382	The logarithm of aqueous solubility value.
logP	6.031	The logarithm of the n-octanol/water distribution coefficients at pH=7.4.
logD	4.262	The logarithm of the n-octanol/water distribution coefficient.
pka (Acid)	11.398	Acid-base dissociation constant (pKa) value represents the strength of a drug molecule's acidity or basicity.
pka (Base)	2.57	Acid-base dissociation constant (pKa) value represents the strength of a drug molecule's acidity or basicity.
Melting point	148.095	The predicted melting point of a compound is expressed in degrees Celsius (°C).<br>Melting points below 25°C are classified as liquids, while melting points above 25°C are classified as solids.
Boiling point	447.131	The predicted melting point of a compound is expressed in degrees Celsius (°C).<br>A normal boiling point below 25°C is categorized as a gas.

Table S3. Medicinal Chemistry of compound **9l**

Property	9l	Comment
QED	0.088	■ A measure of drug-likeness based on the concept of desirability;<br>■ Attractive: > 0.67;<br>■ unattractive: 0.49~0.67;<br>■ too complex: < 0.34
GASA	0.0	■ ES: Easy to synthesize; HS: Hard to synthesize;<br>■ The output value represents the probability of being difficult to synthesize, ranging from 0 to 1.
Synth	2.0	■ Synthetic accessibility score is designed to estimate ease of synthesis of drug-like molecules.<br>■ SAscore □ 6, difficult to synthesize; SAscore <6, easy to synthesize
Fsp3	0.125	■ The number of sp3 hybridized carbons / total carbon count, correlating with melting point and solubility.<br>■ Fsp^3 □0.42 is considered a suitable value.
MCE-18	28.0	■ MCE-18 stands for medicinal chemistry evolution.<br>■ MCE-18□45 is considered a suitable value.
NPscore	-1.228	■ Natural product-likeness score.<br>■ This score is typically in the range from -5 to 5.<br>■ The higher the score is, the higher the probability is that the molecule is a NP.
Lipinski Rule	1.0	■ MW □ 500; logP □ 5; Hacc □ 10; Hdon □ 5<br>■ If two properties are out of range, a poor absorption or permeability is possible, one is acceptable.
Pfizer Rule	1.0	■ logP > 3; TPSA < 75<br>■ Compounds with a high log P (>3) and low TPSA (<75) are likely to be toxic.
GSK Rule	1.0	■ MW □ 400; logP □ 4<br>■ Compounds satisfying the GSK rule may

		have a more favorable ADMET profile
Golden Triangle	1.0	■ 200 □ MW □ 500; -2 □ logD □ 5<br>■ Compounds satisfying the Golden Triangle rule may have a more favorable ADMET profile.
PAINS	0 alerts	frequent hitters, Alpha-screen artifacts and reactive compound 480 substructures (J Med Chem 201053:2719-40)
ALARM NMR	6 alerts	Thiol reactive compounds.
BMS	0 alerts	undesirable, reactive compounds 176 substructures (J Chem Inf Model 200646:1060-8)
Chelator Rule	0 alerts	Chelating compounds.
Colloidal aggregators	1.0	■ Category 0: non-colloidal aggregators;<br>■ Category 1: colloidal aggregators.<br>■ The output value is the probability of being colloidal aggregators, within the range of 0 to 1.
FLuc inhibitors	1.0	■ Category 0: non-fLuc inhibitors;<br>■ Category 1: fLuc inhibitors.<br>The output value is the probability of being fLuc inhibitors, within the range of 0 to 1.
Blue fluorescence	0.582	■ Category 0: non-blue fluorescence;<br>■ Category 1: blue fluorescence.<br>The output value is the probability of being blue fluorescence, within the range of 0 to 1.
Green fluorescence	1.0	■ Category 0: non-green fluorescence;<br>■ Category 1: green fluorescence.<br>The output value is the probability of being green fluorescence, within the range of 0 to 1.
Reactive compounds	0.02	■ Category 0: non-reactive compound;<br>■ Category 1: reactive compound.<br>The output value is the probability of being reactive compound, within the range of 0 to 1.
Promiscuous compounds	0.006	■ Category 0: non-promiscuous compound;<br>■ Category 1: promiscuous compound.<br>The output value is the probability of being promiscuous compound, within the range of 0 to 1.

Table S4. Absorbtion of compound **9l**

Property	9l	Comment
Caco-2	-5.161	Optimal: higher than -5.15 Log unit

Permeability		
MDCK Permeability	-4.641	■ low permeability: < 2 × 10-6 cm/s<br>■ medium permeability: 2-20 × 10^{-6} cm/s<br>■ high passive permeability: > 20 × 10^{-6} cm/s
PAMPA	0.009	■ The experimental data for Peff was logarithmically transformed (logPeff).<br>■ Molecules with log Peff values below 2.0 were classified as low-permeability (Category 0), while those with log Peff values exceeding 2.5 were classified as high-permeability (Category 1).
Pgp-inhibitor	1.0	■ Category 1: Inhibitor;<br>■ Category 0: Non-inhibitor;<br>■ The output value is the probability of being Pgp-inhibitor
Pgp-substrate	0.0	■ Category 1: substrate;<br>■ Category 0: Non-substrate;<br>■ The output value is the probability of being Pgp-substrate
HIA	0.0	■ Human Intestinal Absorption<br>■ Category 1: HIA+(HIA < 30%);<br>■ Category 0: HIA-(HIA >= 30%);<br>■ The output value is the probability of being HIA+
$F_{20\%}$	0.0	■ 20% Bioavailability<br>■ Category 1: F 20% + (bioavailability < 20%);<br>■ Category 0: F 20% - (bioavailability □ 20%);<br>■ The output value is the probability of being F 20%<br>+
$F_{30\%}$	0.037	■ 30% Bioavailability<br>■ Category 1: F 30% + (bioavailability < 30%);<br>■ Category 0: F 30% - (bioavailability □ 30%);<br>■ The output value is the probability of being F 30%<br>+
$F_{50\%}$	0.064	■ 50% Bioavailability<br>■ Category 1: F 50% + (bioavailability < 50%);<br>■ Category 0: F 50% - (bioavailability □ 50%);<br>■ The output value is the probability of being F 50%<br>+

Table S5. Distribution of compound **9l**

**Property**	**9l**	**Comment**

PPB	99.447	■ Plasma Protein Binding Optimal: < 90%.<br>■ Drugs with high protein-bound may have a low therapeutic index.
VDss	0.174	■ Volume Distribution<br>■ Optimal: 0.04-20L/kg
BBB	0.067	■ Blood-Brain Barrier Penetration<br>■ Category 1: BBB+; Category 0: BBB-;<br>■ The output value is the probability of being BBB+
Fu	0.438	■ The fraction unbound in plasms<br>■ Low: <5%; Middle: 5~20%; High: > 20%
OATP1B1 inhibitor	1.0	■ Category 0: Non-inhibitor; Category 1: inhibitor.<br>■ The output value is the probability of being inhibitor, within the range of 0 to 1.
OATP1B3 inhibitor	1.0	■ Category 0: Non-inhibitor; Category 1: inhibitor.<br>■ The output value is the probability of being inhibitor, within the range of 0 to 1.
BCRP inhibitor	0.0	■ Category 0: Non-inhibitor; Category 1: inhibitor.<br>■ The output value is the probability of being inhibitor, within the range of 0 to 1.
MRP1 inhibitor	0.797	■ Category 0: Non-inhibitor; Category 1: inhibitor.<br>■ The output value is the probability of being inhibitor, within the range of 0 to 1.

Table S6. Metabolism of compound **9l**

Property	9l	Comment
CYP1A2 inhibitor	1.0	■ Category 1: Inhibitor; Category 0: Non-inhibitor;<br>■ The output value is the probability of being inhibitor.
CYP1A2 substrate	0.052	■ Category 1: Substrate; Category 0: Non-substrate;<br>The output value is the probability of being substrate.
CYP2C19 inhibitor	1.0	■ Category 1: Inhibitor; Category 0: Non-inhibitor;<br>The output value is the probability of being inhibitor.
CYP2C19	0.0	■ Category 1: Substrate; Category 0: Non-

substrate		substrate;<br>The output value is the probability of being substrate.
CYP2C9 inhibitor	1.0	■ Category 1: Inhibitor; Category 0: Non-inhibitor;<br>The output value is the probability of being inhibitor.
CYP2C9 substrate	0.188	■ Category 1: Substrate; Category 0: Non-substrate;<br>The output value is the probability of being substrate.
CYP2D6 inhibitor	0.965	■ Category 1: Inhibitor; Category 0: Non-inhibitor;<br>The output value is the probability of being inhibitor.
CYP2D6 substrate	0.57	■ Category 1: Substrate; Category 0: Non-substrate;<br>The output value is the probability of being substrate.
CYP3A4 inhibitor	0.708	■ Category 1: Inhibitor; Category 0: Non-inhibitor;<br>The output value is the probability of being inhibitor.
CYP3A4 substrate	0.0	■ Category 1: Substrate; Category 0: Non-substrate;<br>The output value is the probability of being substrate.
CYP2B6 inhibitor	1.0	■ Category 1: Inhibitor; Category 0: Non-inhibitor;<br>The output value is the probability of being inhibitor.
CYP2B6 substrate	0.0	■ Category 1: Substrate; Category 0: Non-substrate;<br>The output value is the probability of being substrate.
CYP2C8 inhibitor	1.0	■ Category 1: Inhibitor; Category 0: Non-inhibitor;<br>The output value is the probability of being inhibitor.
HLM Stability	0.256	■ human liver microsomal (HLM) stability<br>Category 0: stable+ (HLM > 30 min); Category 1: unstable- (HLM □ 30 min). The output value is the probability of human liver microsomal instability, where a value closer to 1 indicates a higher likelihood of instability. The range is between 0 and 1.

Table S7. Excretion of compound **9l**

**Property**	**9l**	**Comment**

CL_{plasma}	2.571	■ The unit of predicted CLplasma penetration is ml/min/kg. >15 ml/min/kg: high clearance; 5-15 ml/min/kg: moderate clearance; < 5 ml/min/kg: low clearance.
$T_{1/2}$	0.914	■ The unit of predicted T1/2 is hours.<br>■ ultra-short half-life drugs: 1/2 < 1 hour; short half-life drugs: T1/2 between 1-4 hours; intermediate short half-life drugs: T1/2 between 4-8 hours; long half-life drugs: T1/2 > 8 hours.

Table S8. Toxicity of compound **9l**

Property	9l	Comment
hERG Blockers	0.861	■ Molecules with IC50 □10□M or □50% inhibition at 10 □M were classified as hERG+ (Category 1),<br>■ while molecules with IC50 >10□M or < 50% inhibition at 10□M were classified as hERG - (Category 0).<br>■ The output value is the probability of being hERG+, within the range of 0 to 1.
hERG Blockers (10um)	0.84	■ Molecules with IC50 □10 □M are classified as hERG+ (Category 1),<br>■ and molecules with IC50 > 10□M are classified as hERG- (Category 0).<br>■ The output value is the probability of being hERG+, within the range of 0 to 1.
DILI	0.997	■ Drug Induced Liver Injury.<br>■ Category 1: drugs with a high risk of DILI;<br>■ Category 0: drugs with no risk of DILI.<br>■ The output value is the probability of being toxic.
AMES Mutagenicity	0.215	■ AMES Toxicity<br>■ Category 1: Ames positive(+);<br>■ Category 0: Ames negative(-);<br>■ The output value is the probability of being toxic.
Rat Oral Acute Toxicity	0.163	■ Rat Oral Acute Toxicity.<br>■ Category 0: low-toxicity, > 500 mg/kg;<br>■ Category 1: high-toxicity; < 500 mg/kg.<br>■ The output value is the probability of being toxic, within the range of 0 to 1.
FDAMDD	0.363	■ FDA Maximum (Recommended) Daily Dose.<br>■ Category 1: FDAMDD (+);<br>■ Category 0: FDAMDD (-);<br>The output value is the probability of being

		positive.
Skin Sensitization	0.801	■ Category 1: Sensitizer;<br>■ Category 0: Non-sensitizer.<br>■ The output value is the probability of being toxic, within the range of 0 to 1.
Carcinogenicity	0.369	■ Category 1: carcinogens;<br>■ Category 0: non-carcinogens;<br>■ The output value is the probability of being toxic.
Eye Corrosion	0.0	■ Eye Corrosion<br>■ Category 1: corrosives; Category 0: noncorrosives;<br>The output value is the probability of being corrosives.
Eye Irritation	0.031	■ Eye Irritation<br>■ Category 1: irritants; Category 0: nonirritants;<br>The output value is the probability of being irritants.
Respiratory	0.441	■ Category 1: respiratory toxicants;<br>■ Category 0: non-respiratory toxicants.<br>The output value is the probability of being toxic, within the range of 0 to 1.
Human Hepatotoxicity	0.701	■ Human Hepatotoxicity<br>■ Category 1: H-HT positive(+);<br>■ Category 0: H-HT negative(-);<br>The output value is the probability of being toxic.
Drug-induced Nephrotoxicity	0.871	■ Category 0: non-nephrotoxic (-);<br>■ Category 1: nephrotoxic (+).<br>The output value is the probability of being nephrotoxic (+), within the range of 0 to 1.
Ototoxicity	0.61	■ Category 0: non-ototoxicity (-);<br>■ Category 1: ototoxicity (+).<br>The output value is the probability of being ototoxicity (+), within the range of 0 to 1.
Hematotoxicity	0.187	■ Category 0: non-hematotoxicity (-);<br>■ Category 1: hematotoxicity (+).<br>The output value is the probability of being hematotoxicity (+), within the range of 0 to 1.
Genotoxicity	0.995	■ Category 0: non-Genotoxicity (-);<br>■ Category 1: Genotoxicity (+).<br>The output value is the probability of being ototoxicity (+), within the range of 0 to 1.
RPMI-8226 Immunitoxicity	0.029	■ Category 0: non-cytotoxicity (-);<br>■ Category 1: cytotoxicity (+).

		The output value is the probability of being ototoxicity (+), within the range of 0 to 1.
A549 Cytotoxicity	0.154	■ Category 0: non-cytotoxicity (-);<br>■ Category 1: cytotoxicity (+).<br>The output value is the probability of being ototoxicity (+), within the range of 0 to 1.
Hek293 Cytotoxicity	0.902	■ Category 0: non-cytotoxicity (-);<br>■ Category 1: cytotoxicity (+).<br>The output value is the probability of being ototoxicity (+), within the range of 0 to 1.
Drug-induced Neurotoxicity	0.953	■ Category 0: non-neurotoxic (-);<br>■ Category 1: neurotoxic (+).<br>The output value is the probability of being neurotoxic (+), within the range of 0 to 1.

Table S9. Environmental toxicity of compound **9l**

**Property**	**9l**	**Comment**
Bioconcentration Factors	1.261	■ Bioconcentration factors are used for considering secondary poisoning potential and assessing risks to human health via the food chain.<br>The unit is □log10[(mg/L)/(1000*MW)]
IGC50	5.37	■ Tetrahymena pyriformis 50 percent growth inhibition concentration.<br>The unit is □log10[(mg/L)/(1000*MW)]
LC50FM	7.376	■ 96-hour fathead minnow 50 percent lethal concentration.<br>The unit is □log10[(mg/L)/(1000*MW)]
LC50DM	7.381	■ 48-hour daphnia magna 50 percent lethal concentration.<br>The unit is □log10[(mg/L)/(1000*MW)]

Table S10. Tox21 pathway of compound **9l**

**Property**	**9l**	**Comment**
NR-AhR	0.817	■ Aryl hydrocarbon receptor<br>■ Category 1: actives ;<br>■ Category 0: inactives;<br>■ The output value is the probability of being active.
NR-AR	0.0	■ Androgen receptor<br>■ Category 1: actives ;<br>■ Category 0: inactives;

		■ The output value is the probability of being active.
NR-AR-LBD	0.203	■ Androgen receptor ligand-binding domain<br>■ Category 1: actives ;<br>■ Category 0: inactives;<br>■ The output value is the probability of being active.
NR-Aromatase	0.095	■ Category 1: actives ;<br>■ Category 0: inactives;<br>■ The output value is the probability of being active.
NR-ER	0.893	■ Estrogen receptor<br>■ Category 1: actives ;<br>■ Category 0: inactives;<br>■ The output value is the probability of being active.
NR-ER-LBD	0.001	■ Estrogen receptor ligand-binding domain<br>■ Category 1: actives ;<br>■ Category 0: inactives;<br>■ The output value is the probability of being active.
NR-PPAR-gamma	0.258	■ Peroxisome proliferator-activated receptor gamma<br>■ Category 1: actives ;<br>■ Category 0: inactives;<br>■ The output value is the probability of being active.
SR-ARE	0.985	■ Antioxidant response element<br>■ Category 1: actives ;<br>■ Category 0: inactives;<br>■ The output value is the probability of being active.
SR-ATAD5	0.302	■ ATPase family AAA domain-containing protein 5<br>■ Category 1: actives ;<br>■ Category 0: inactives;<br>The output value is the probability of being active.
SR-HSE	0.866	■ Heat shock factor response element<br>■ Category 1: actives ;<br>■ Category 0: inactives;<br>The output value is the probability of being active.
SR-MMP	0.994	■ Mitochondrial membrane potential<br>■ Category 1: actives ;<br>■ Category 0: inactives;<br>The output value is the probability of being active.
SR-p53	0.898	■ p53, a tumor suppressor protein<br>■ Category 1: actives ;<br>■ Category 0: inactives;<br>The output value is the probability of being active.

Table S11. Toxicophore Rules of compound **9l**

Property	9l	Comment
Acute Toxicity Rule	0	■ 20 substructures;<br>acute toxicity during oral administration
Genotoxic Carcinogenicity Rule	1 alerts	■ 117 substructures;<br>carcinogenicity or mutagenicity
NonGenotoxic Carcinogenicity Rule	2 alerts	■ 23 substructures;<br>carcinogenicity through nongenotoxic mechanisms
Skin Sensitization Rule	3 alerts	■ 155 substructures;<br>skin irritation
Aquatic Toxicity Rule	3 alerts	■ 99 substructures;<br>toxicity to liquid(water)
NonBiodegradale Rule	2 alerts	■ 19 substructures;<br>non-biodegradable
SureChEMBL Rule	0	■ 164 substructures;<br>MedChem unfriendly status
FAF-Drugs4 Rule	5 alerts	154 toxic substructures from FAF-Drug4

2. Experimental

2.1. Chemistry

General Information

All reagents and solvents were of general purpose or analytical grade and purchased from Sigma Aldrich Ltd, Fisher Scientific, Fluka and Acros. ^{1}H- and ^{13}C-NMR spectra were recorded with a Bruker Avance III spectrometer operating at 500, 120 MHz respectively, with Me_4Si as internal standard and DMSO-d_6 as a solvent. Elemental analysis was performed by the regional center for mycology and biotechnology (Cairo, Egypt). TLC was carried out on precoated silica plates (Keisel gel 60 F254, BDH) using Hexane: Ethyl acetate, 1 : 2, v/v. Compounds were visualized by illumination under UV light (254 nm). Melting points were determined on an electrothermal instrument and are uncorrected. All solvents were dried prior to use and stored over 4 Å molecular sieves, under nitrogen.

2.1. Antiproliferative Assay

The cytotoxic activity of compounds 9a–o was evaluated in comparison with the reference drugs combretastatin A-4 (CA-4) and doxorubicin against three human cancer cell lines: MDA-MB-231 (triple-negative breast carcinoma), HCA-7 (colon adenocarcinoma), and A549 (lung carcinoma), as well as the non-tumorigenic human mammary epithelial cell line MCF-10A. Cells were cultured in their respective recommended media supplemented with 10% fetal bovine serum and 1% penicillin–streptomycin and maintained at 37 °C in a humidified atmosphere containing 5% CO_2.

Cell viability was assessed using the MTT assay following the protocol provided with the In Vitro Toxicology Assay Kit, MTT-based (Sigma-Aldrich, TOX-1). Briefly, cells were seeded in 96-well plates at a density of 5×10^3 cells/well and allowed to adhere overnight. The following day, the cells were treated with increasing concentrations of compounds 9a–o, CA-4, or doxorubicin and

incubated for 48 h. After treatment, 10 μL of reconstituted MTT solution (5 mg/mL in serum-free, phenol red-free medium) was added to each well, and the plates were incubated for an additional 3 h at 37 °C. The resulting formazan crystals were then dissolved by adding 100 μL of MTT solubilization solution, and the plates were gently shaken to ensure complete solubilization. Absorbance was measured at 570 nm with a reference wavelength of 690 nm using a microplate reader. Cell viability was expressed as a percentage relative to untreated control cells. All experiments were performed in triplicate ($n = 3$), and IC_{50} values were calculated by nonlinear regression analysis.

2.2. Tubulin Polymerization Inhibitory Assay

The effects of compounds 9a–o on tubulin polymerization were evaluated in comparison with the reference microtubule-destabilizing agent combretastatin A-4 (CA-4) using the Tubulin Polymerization Assay Kit (fluorescence-based, Cytoskeleton, Inc., Cat. No. BK011P), according to the manufacturer's protocol. This fluorescence-based assay monitors the polymerization of purified porcine brain tubulin by detecting the enhanced fluorescence signal resulting from incorporation of a fluorescent reporter into assembling microtubules.

Compounds 9a–o and CA-4 were prepared as 10× stock solutions in DMSO and diluted to the desired concentrations in assay buffer. Each well of a pre-warmed 96-well black flat-bottom plate received 5 μL of compound solution or vehicle control. The polymerization reaction was initiated by adding 50 μL of the tubulin reaction mixture (2 mg/mL tubulin in 80 mM PIPES, pH 6.9, 2.0 mM $MgCl_2$, 0.5 mM EGTA, 1.0 mM GTP, and 15% glycerol). The plate was immediately transferred to a temperature-controlled microplate reader pre-equilibrated at 37 °C. Tubulin polymerization was monitored kinetically by recording fluorescence at 360 nm excitation and 420 nm emission every minute for 60 min. Control wells included vehicle (DMSO), paclitaxel as a

polymerization enhancer, and vinblastine as a polymerization inhibitor. The inhibitory effects of compounds 9a–o and CA-4 were determined by comparing fluorescence signals with the vehicle control. The assay was performed in triplicate (n = 3), and IC_{50} values were calculated by linear regression analysis.

2.3. COX inhibition assay

The inhibitory activities of compounds 9h, 9j, 9l, 9m, and 9o against cyclooxygenase isoforms were evaluated using the Cayman COX (ovine/human) Inhibitor Screening Assay Kit (Item No. 560131; Cayman Chemical, Ann Arbor, MI, USA). The assay directly measures $PGF_{2}\alpha$ generated after $SnCl_2$ reduction of COX-derived PGH_2, followed by ELISA-based quantification, and employs ovine COX-1 together with human recombinant COX-2. Briefly, reaction mixtures containing 1× reaction buffer, heme, and the corresponding enzyme were preincubated with the tested compounds or vehicle control for 10 min at 37 °C. The reactions were initiated by addition of arachidonic acid and allowed to proceed for 2 min at 37 °C, then terminated with saturated stannous chloride solution and further incubated for 15 min at 37 °C. The resulting $PGF_{2}\alpha$ was quantified by ELISA using the supplied $PGF_{2}\alpha$ standard, $PGF_{2}\alpha$ AChE tracer, and $PGF_{2}\alpha$ antiserum, and absorbance was recorded at 412 nm. Vehicle control wells (0.1% DMSO) and blank wells lacking active enzyme were included to account for non-specific effects and background signal. Each concentration was tested in triplicate, and the experiments were independently repeated three times. The inhibitory activities toward COX-1 and COX-2 were expressed as IC_{50} values, and the selectivity index (SI) was calculated as IC_{50}(COX-1)/IC_{50}(COX-2).

2.4. Cell cycle analysis

HCA-7 cells were seeded and treated with compound 9l at its IC_{50} concentration against HCA-7 cells (2.18 μM) for 48 h. After treatment, both adherent and floating cells were collected to avoid loss of mitotic and apoptotic populations, washed with PBS, and pelleted by centrifugation (500 × g, 5 min). The cells were then fixed in 66% ice-cold ethanol added dropwise while vortexing and stored at 4 °C for at least 2 h to ensure permeabilization and stabilization of DNA content. Prior to analysis, the fixed cells were washed with PBS and resuspended in propidium iodide/RNase staining solution, followed by incubation for 20–30 min at 37 °C in the dark. Samples were analyzed using a flow cytometer equipped with a 488 nm laser, and PI fluorescence was collected in the FL2 channel. Debris and cell aggregates were excluded by forward and side scatter gating. DNA histograms were generated, and the percentages of cells in G0/G1 (2N), S (2N–4N), and G2/M (4N) phases were quantified using dedicated flow cytometry analysis software. The presented cell-cycle histogram and phase-distribution percentages correspond to a single representative flow-cytometric experiment (n = 1).

2.5. Apoptosis assay

Apoptosis induced by compound 9l in HCA-7 cells was evaluated using Annexin V-FITC/propidium iodide (PI) dual staining followed by flow cytometric analysis. Briefly, HCA-7 cells were seeded and treated with compound 9l at its IC_{50} concentration against HCA-7 cells (2.18 μM) for 48 h. After treatment, both floating and adherent cells were collected to avoid underestimation of apoptotic populations, washed twice with cold phosphate-buffered saline (PBS), and centrifuged to obtain a single-cell suspension. Cells (1–5 × 10^5) were resuspended in 500 μL of binding buffer and incubated with Annexin V-FITC (5 μL) and PI (5 μL) for 5 min at room temperature in the dark to allow detection of phosphatidylserine externalization and loss of membrane integrity. Samples were subsequently analyzed by flow cytometry using 488 nm

excitation, with FITC fluorescence detected in the FL1 channel and PI fluorescence detected in the FL2 channel. Appropriate forward- and side-scatter gating was applied to exclude debris and cell aggregates, and quadrant analysis was performed to discriminate viable (Annexin V^-/PI^-), early apoptotic (Annexin V^+/PI^-), late apoptotic (Annexin V^+/PI^+), and necrotic (Annexin V^-/PI^+) cell populations. The percentages of cells in each quadrant were quantified using dedicated flow cytometry analysis software. The presented Annexin V-FITC/PI dot plot and apoptotic-population percentages correspond to a single representative flow-cytometric experiment (n = 1).

2.6. Effect on *BAX* expression levels

The effect of compound 9l on BAX protein expression in HCA-7 cells was determined using the Human BAX ELISA Kit (DRG International, Inc., Cat. No. EIA-4487), according to the manufacturer's instructions. This sandwich-based immunoassay employs a monoclonal antibody specific for human BAX-α to capture and quantify BAX in cell lysates. HCA-7 cells were treated with compound 9l at its IC_{50} concentration for 48 h. After treatment, the cells were lysed using the provided Cell Lysis Buffer, freshly supplemented with phenylmethylsulfonyl fluoride (PMSF, 1 mM) and protease inhibitor cocktail (PIC, 0.5 µL/mL). The lysates were incubated on ice for 15 min, briefly vortexed, and centrifuged at 16,000 rpm for 15 min. The resulting supernatants were collected and diluted with the kit Assay Buffer before analysis. A standard curve was generated using recombinant human BAX-α standards at concentrations of 62.5, 125, 250, 500, 1000, and 2000 pg/mL. Standards, controls, and samples were added at 100 µL/well and run in duplicate according to the kit protocol. The plate was incubated at room temperature for 1 h on a plate shaker at approximately 500 rpm, washed five times, and then incubated with 100 µL of biotinylated anti-BAX antibody for 1 h at room temperature. After another five washes, 100 µL of streptavidin-HRP conjugate was added and incubated for 30 min at room temperature. The wells were washed again

five times, followed by the addition of 100 μL TMB substrate and incubation for 30 min at room temperature. The reaction was stopped by adding 100 μL stop solution, and absorbance was measured at 450 nm, with correction at 570–590 nm. Net absorbance values were obtained by subtracting the blank OD from each standard and sample reading. BAX concentrations in the treated samples were calculated from the standard curve using linear regression analysis and expressed as mean ± SEM.

2.7. Effect on *Bcl-2* expression levels

The effect of compound 9l on Bcl-2 protein expression in HCA-7 cells was quantified using the Zymed® Bcl-2 ELISA Kit (Cat. No. 99-0042), according to the manufacturer's instructions. This assay employs a sandwich ELISA format in which human Bcl-2 is captured by a monoclonal antibody immobilized on a microwell plate and subsequently detected using a biotin-conjugated anti-Bcl-2 antibody, followed by streptavidin-HRP and TMB substrate for colorimetric detection. HCA-7 cells were treated with compound 9l at its IC_{50} concentration for 48 h. After treatment, the cells were lysed in the provided Lysis Buffer at a density of 5×10^6 cells/mL and incubated at room temperature for 1 h with gentle shaking. The lysates were clarified by centrifugation at 1000 × g for 15 min, and the resulting supernatants were collected and used immediately or stored at −80 °C until analysis. For the assay, standards, blanks, and samples were run in duplicate. Sample wells received 80 μL of Sample Diluent and 20 μL of sample, followed by the addition of 50 μL of diluted biotin-conjugated anti-Bcl-2 antibody. After incubation for 2 h at room temperature, the plate was washed three times, and 100 μL of diluted streptavidin-HRP was added to each well. The plate was then incubated for 1 h at room temperature, washed again three times, and 100 μL of mixed TMB substrate solution was added. Color development was allowed to proceed for 15 min at room temperature, protected from intense light, before the reaction was terminated with 100 μL of stop

solution. Absorbance was measured at 450 nm using a microplate reader, with optional correction at 620 nm or within the acceptable reference range of 610–650 nm. A standard curve was generated using Bcl-2 standards prepared by serial dilution over the range of 0.5–32 ng/mL. Net absorbance values were obtained by subtracting the blank reading, and Bcl-2 concentrations in the test samples were calculated from the standard curve using linear regression analysis.

2.8. Effect of *caspase-3* activity

The level of active caspase-3 following treatment with compound 9l was quantified in HCA-7 cells using the Human Active Caspase-3 ELISA Kit (Invitrogen, Cat. No. KHO1091), according to the manufacturer's instructions. This assay is based on a solid-phase sandwich ELISA principle, in which an anti-human caspase-3 monoclonal antibody is pre-coated onto a microplate. Cell lysates or standards are captured by the immobilized antibody, followed by the addition of a rabbit polyclonal detection antibody specific for active caspase-3 cleaved at Asp175/Ser176. Detection is subsequently achieved using HRP-conjugated anti-rabbit IgG and TMB substrate. HCA-7 cells were treated with compound 9l at its IC_{50} concentration for 48 h. After treatment, the cells were lysed using cell extraction buffer freshly supplemented with PMSF and protease inhibitor cocktail. The lysates were incubated on ice for 30 min with vortexing at 10 min intervals, then clarified by centrifugation at 13,000 rpm for 10 min at 4 °C. The clear lysates were collected and diluted in Standard Diluent Buffer before analysis. Standards, controls, and samples were run in duplicate according to the kit recommendation. A standard curve was generated using human active caspase-3 standards at concentrations of 39, 78, 156, 313, 625, 1250, and 2500 pg/mL. Aliquots of 100 µL of standards, controls, or diluted samples were added to the appropriate wells and incubated for 2 h at room temperature. The wells were then washed four times, followed by addition of 100 µL of active caspase-3 detection antibody and incubation for 1 h at room temperature. After another four washes,

100 μL of anti-rabbit IgG-HRP working solution was added and incubated for 30 min at room temperature. The wells were washed again four times, followed by addition of 100 μL of stabilized TMB chromogen and incubation for 30 min at room temperature in the dark. The reaction was stopped by adding 100 μL stop solution, and absorbance was measured at 450 nm within the recommended reading time. Net absorbance values were obtained after subtraction of the blank reading, and active caspase-3 concentrations in the treated samples were calculated from the standard curve using linear regression analysis and corrected for the applied dilution factor where applicable.

2.9. Effect of *caspase-9* activity

The concentration of caspase-9 following treatment with compound 9l was quantified in HCA-7 cells using the Human Caspase-9 ELISA Kit (Thermo Fisher Scientific, Cat. No. BMS2025), according to the manufacturer's protocol. This solid-phase sandwich ELISA employs a monoclonal antibody pre-coated onto a 96-well microplate to capture caspase-9 from biological samples. A rabbit polyclonal detection antibody specific for human caspase-9 is then added, followed by HRP-conjugated anti-rabbit IgG and TMB substrate for colorimetric detection. The intensity of the yellow reaction product formed after termination with phosphoric acid is proportional to the amount of caspase-9 present and was measured at 450 nm. HCA-7 cells were treated with compound 9l at its IC_{50} concentration for 48 h. After treatment, HCA-7 cells were lysed in the supplied 1× lysis buffer at a density of approximately 5×10^6 cells/mL and incubated for 60 min at room temperature with gentle shaking. The lysates were then centrifuged at 1000 × g for 15 min, and the clarified supernatants were collected and either analyzed immediately or stored at −80 °C until use. For the assay, standards, blanks, and samples were run in duplicate. Standard dilutions were prepared by 1:2 serial dilution of the reconstituted human caspase-9 standard to generate concentrations of 100,

50, 25, 12.5, 6.3, 3.1, and 1.6 ng/mL. For sample wells, 50 µL of Sample Diluent and 50 µL of sample were added, corresponding to a 1:2 sample dilution. Then, 50 µL of detection antibody was added to all wells, and the plate was incubated for 2 h at room temperature (18–25 °C) on a microplate shaker. The wells were washed three times, followed by addition of 100 µL of diluted anti-rabbit-IgG-HRP and incubation for 1 h at room temperature. After another three washes, 100 µL of TMB substrate solution was added and incubated for approximately 10 min at room temperature, protected from intense light. The reaction was stopped by adding 100 µL of stop solution, and absorbance was measured at 450 nm, with optional reference correction at 620 nm or within the acceptable range of 610–650 nm. Net OD values were obtained by subtracting the blank reading, and caspase-9 concentrations in the treated samples were calculated from the standard curve using linear regression analysis. The final concentrations were corrected for the 1:2 dilution factor.

2.10. Effect on PGE-2 Production

The effect of compound 9l on prostaglandin E2 (PGE-2) production was evaluated in HCA-7 human colon cancer cells using a commercially available competitive ELISA kit (Parameter™ PGE-2 Immunoassay, R&D Systems, Catalog No. KGE004B). HCA-7 cells were treated with compound 9l (1.0 µM) for 24 h, after which the culture supernatants were collected, centrifuged to remove debris, and either analyzed immediately or stored at −20 °C until use. Samples were diluted 3-fold with Calibrator Diluent RD5-56 and assayed according to the manufacturer's instructions. Briefly, 150 µL of each diluted sample or standard was added to the antibody-coated plate, followed by 50 µL of primary antibody solution. After incubation for 1 h at room temperature on a shaker, 50 µL of PGE-2 conjugate was added and the plate was incubated for an additional 2 h. The wells were then washed four times, incubated with substrate solution for 30 min in the dark, and the

reaction was terminated by addition of stop solution. Absorbance was measured at 450 nm with correction at 540 or 570 nm. Standards, controls, and samples were assayed in duplicate. PGE-2 concentrations were calculated from the standard curve by linear regression.

2.11. Wound Healing Assay

The effect of compound 9l on the migratory capacity of HCA-7 cells was evaluated using the CytoSelect™ 24-Well Wound Healing Assay (Cell Biolabs, Inc., Cat. No. CBA-120), according to the manufacturer's instructions. This insert-based assay generates a defined 0.9 mm cell-free wound field after removal of the insert, allowing assessment of cell migration and wound closure. Under sterile conditions, the plate was equilibrated to room temperature for 10 min, and the inserts were positioned in the wells using sterile forceps. HCA-7 cells were prepared as a suspension at 0.5–1.0 × 10^6 cells/mL in medium containing 10% FBS, and 500 µL was added to each well. The cells were incubated overnight at 37 °C in a humidified 5% CO_2 atmosphere or until a monolayer formed around the inserts. To start the assay, the inserts were carefully removed, the wells were gently washed to remove detached cells and debris, and fresh medium containing compound 9l was added. Control wells received the corresponding vehicle. Images were captured immediately after insert removal at 0 h to document the initial wound field, and wound closure was monitored using a light microscope and imaging software. The final wound-closure assessment was performed after 72 h of incubation, because this time point allowed near-complete wound closure in the vehicle-treated control and provided a suitable dynamic range for evaluating the effect of compound 9l on wound closure. Representative images were acquired for the vehicle control and compound 9l-treated condition, and wound closure was quantified from the analyzed wound fields by comparing the wound area at 72 h with the initial wound area at 0 h.

2.12. Metabolic Stability Studies

The metabolic stability of compound 9l was evaluated using pooled human liver microsomes (HLM) under NADPH-supported oxidative conditions. Incubations were carried out in 100 mM potassium phosphate buffer (pH 7.4) containing HLM at a final microsomal protein concentration of 0.5 mg/mL. Compound 9l was added from a DMSO stock to final concentrations of 0.8, 4, and 20 µM, while keeping the final DMSO content at ≤0.1% (v/v). After pre-equilibration at 37 °C, reactions were initiated by addition of NADPH (final concentration ~1 mM), and this point was defined as t = 0. At predetermined time points over 0–60 min, aliquots were withdrawn and immediately quenched with ice-cold acetonitrile containing diazepam as an internal standard to terminate enzymatic activity and precipitate proteins. Samples were vortexed, kept on ice, and centrifuged, and the resulting supernatants were subjected to LC–MS/MS analysis. Parent compound depletion was determined from the peak-area ratio of compound 9l to the internal standard at each time point. For each incubation, $\log_{10}$-transformed peak-area ratios were plotted against time (h) and analyzed by linear regression to obtain the slope (m). The apparent first-order depletion rate constant was calculated as $k\ (h^{-1}) = -2.303 \times m$, and the microsomal half-life was calculated as $t_{1/2}\ (h) = 0.693/k$. Intrinsic clearance was calculated as $CL_{int,in\ vitro}$ (µL/min/mg) = $1000 \times [k/60]/0.5$. All incubations were performed in duplicate for each concentration (n = 2), and the results were expressed as mean ± SD for $t_{1/2}$ and $CL_{int,in\ vitro}$.

3. Molecular Modeling

3.1. Molecular docking

The crystal structures of tubulin-colchicine complex (PDB code: 4O2B) and cyclooxygenase 2 (PDB code: 1CX2) were downloaded from the Protein Data Bank. For tubulin docking, only chains A and B of 4O2B were retained because they contain the colchicine-binding site at their interface. Structure of compound 9l was drawn and optimized using MarvinSketch and Avogadro molecular

editors. The proteins were prepared using autodock tools where the co-crystallized water molecules and colchicine were removed then kollman charges and polar hydrogens were added. The docking grid was centered on the native colchicine-binding pocket located between chains A and B, with grid coordinates for tubulin set to 15.951 × 66.804 × 43.33 for the x, y, and z axes, respectively, and grid dimensions of 80 × 80 × 80. Autodock vina was used for molecular docking and the best docking poses were visualized using Discovery Studio Visualizer.

3.2. ADMET predictions

The ADMET profile of compound 9l was predicted in silico using the ADMETlab 3.0 web server. The chemical structure of the compound was converted into its corresponding SMILES notation and submitted to the platform under the default settings. The generated output was used to obtain predicted physicochemical descriptors, including molecular weight, lipophilicity, topological polar surface area, hydrogen-bond donor and acceptor counts, rotatable bond number, and aqueous solubility, together with medicinal chemistry and drug-likeness parameters. In addition, predicted pharmacokinetic properties related to absorption, distribution, metabolism, and excretion were collected, including parameters associated with intestinal absorption, permeability, plasma protein binding, blood-brain barrier penetration, cytochrome P450-related metabolism, and clearance behavior. Toxicity-related outputs, including hepatotoxicity, cardiotoxicity, mutagenicity, and other safety-associated alerts, were also recorded. The predicted data were subsequently analyzed to provide a preliminary assessment of the drug-likeness, pharmacokinetic behavior, metabolic liability, and safety profile of compound 9l.
